# A geographical distribution database of the genus *Dysdera* in the Canary Islands (Araneae, Dysderidae)

**DOI:** 10.3897/zookeys.625.9847

**Published:** 2016-10-19

**Authors:** Nuria Macías-Hernández, Salvador de la Cruz López, Marcos Roca-Cusachs, Pedro Oromí, Miquel A. Arnedo

**Affiliations:** 1 Departamento de Biología Animal, Edafología y Geología, Universidad de La Laguna, 38206 La Laguna, Tenerife, Canary Islands, Spain; 2 ULPGC-ULL, CEI Canarias: Campus Atlántico Tricontinental; 3 Biodiversity Research Institute and Department of Evolutionary Biology, Ecology and Environmental Sciences, Universitat de Barcelona, Av. Diagonal 645, 08028, Barcelona, Spain

**Keywords:** Canary Islands, dataset, distribution maps, species richness, spiders

## Abstract

The ground-dweller spider genus *Dysdera* shows very high species richness on the oceanic archipelago of the Canary Islands, providing one of the most outstanding examples of island radiation among spiders, only paralleled by *Tetragnatha* spiders on the Hawaiian archipelago. A georeferenced database of the 48 *Dysdera* species occurring in the Canary Islands was assembled to facilitate ongoing and future research on this remarkable lineage. All species are endemic to the archipelago except for the cosmopolitan *Dysdera crocata*. The dataset consists of 794 distributional records documented from 1971 to 2015, each locality being represented only once per species. Distribution maps are provided for each species, along with basic diversity and distribution information. The database and geographical maps included in this article stand for the most updated, accurate and complete information on the distribution of the spider genus *Dysdera* in the Canary Islands.

## Introduction

**Figure 1.**
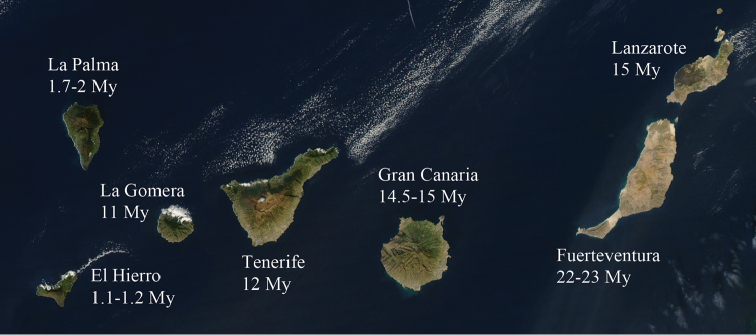
Map of the Canary Islands showing their geological age according to Carracedo et al. (1998) and van den Bogaard (2013).

Because of their high level of endemism and conservation challenges, the Macaronesian archipelagos, located in the eastern Mid-Atlantic ocean, are listed among Earth’s biodiversity hot spots (Myers et al. 2000). The Canary Islands are one of the most diverse and better studied archipelagos in Macaronesia. The islands are of volcanic origin and were never connected to the continent, which lays merely 100 km off the northwest coast of Africa (Fig. 1). The islands are roughly ordered in a straight line. Fuerteventura is the oldest (22–23 My) and closest island to the North African coast. Lanzarote (15 My) is the second oldest and geologically related to Fuerteventura. The rest of the islands become progressively younger towards the west: Gran Canaria (14.5–15 My), Tenerife (12 My), La Gomera (11 My), La Palma (1.7–2 My) and El Hierro (1.1–1.2 My) (Carracedo et al. 1998; van den Bogaard 2013). The major climatic, geological, and ecological differences between Fuerteventura and Lanzarote and the remaining islands are due to the higher erosion and aridification of the former, which have reduced habitat diversity in comparison to the younger central-western islands. Geological and geographic features of the islands have influenced biodiversity patterns in the archipelago. Along the altitudinal gradient, different bioclimatic and vegetation communities characterize the northern and southern slopes of each island. Six main ecological zones can be observed according to altitude, although their limits can differ substantially between windward and leeward slopes, and some zones may even be absent from the leeward slope: (1) the zone from the seashore up to 250 m (700 m on leeward) is characterised by xerophytic shrub communities (lowland xerophytic shrub); (2) from 250 to 600 m the vegetation is dominated by thermo-sclerophyllous woodlands, almost absent on the lee side; (3) from 600 to 1000 m by subtropical mesic to humid laurel forest influenced by the cloud belt, very scarce on leeward slopes, where it is replaced by native pine forest; (4) from 1000 to 2000 m (700 to 2200 on the leeward slope) by a mesic to dry endemic *Pinus canariensis* forest; (5) from 2000–2200 m to 3250 by dry subalpine scrub, and (6) from 3000–3250 m to the top (3718 m on Tenerife), an extremely scarce vegetation is found, with a limited permanent invertebrate community.

**Figure 2.**
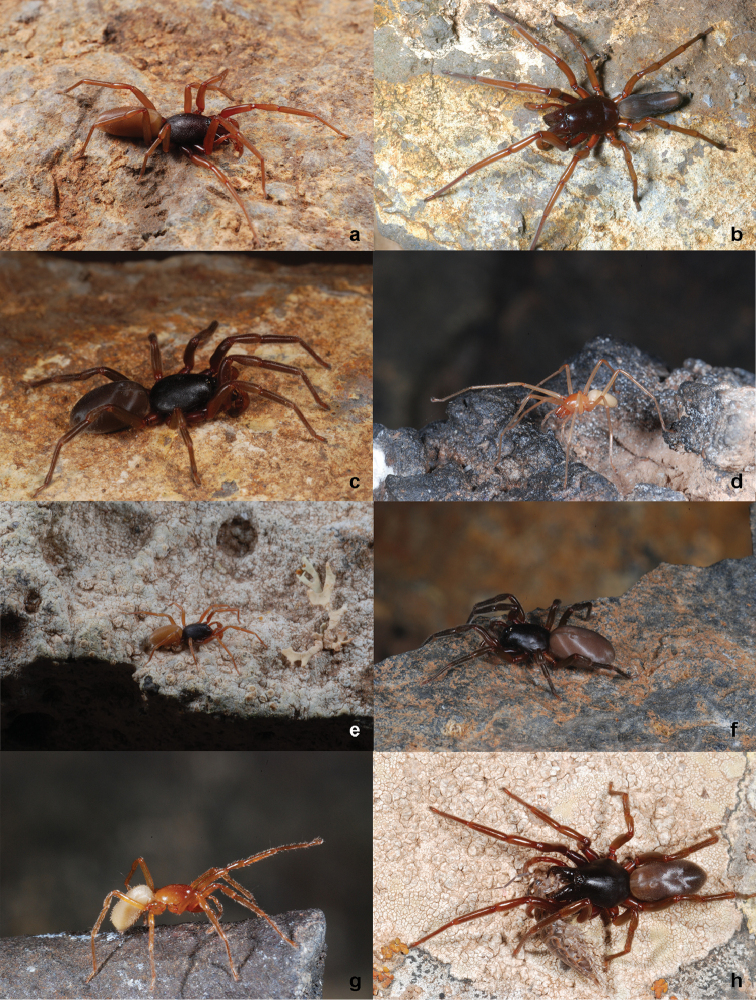
Habitus of some *Dysdera* species: **a** *Dysdera calderensis* **b** *Dysdera longa* **c** *Dysdera verneaui* **d** *Dysdera unguimmanis* **e** *Dysdera minutissima* **f** *Dysdera arabisenen* **g** *Dysdera sibyllina* **h** *Dysdera silvatica*. Photographs by P. Oromí.

Arthropods are by far the most diverse assemblage of terrestrial Canarian organisms, with nearly 7000 species, 465 of which are spiders, most of them endemisms (64%) (Macías-Hernández 2010). The genus *Dysdera* is the richest spider genus in the Canaries. It includes medium size, nocturnal wandering hunters that mostly inhabit humid places, although some species are adapted to mesic and even dry habitats (Fig. 2). During daytime they find shelter in silk cocoons under rocks, trunks and tree barks (Roberts 1995). The genus is distributed along the circum-Mediterranean region, including the Macaronesian archipelagoes (Deeleman-Reinhold and Deeleman 1988), and presently includes approximately 250 species (World Spider Catalog 2016). To date, 47 endemic species have been documented in the Canary Islands (Arnedo et al. 1996, Arnedo and Ribera 1997, Arnedo and Ribera 1999b, Arnedo et al. 2000a, Arnedo et al. 2007, Macías-Hernández et al. 2010). Most of the species are single island endemics, but a few are present in two or three islands. The synanthropic *Dysdera crocata* Koch, 1839 has also been reported on most of the islands.

During the last 20 years several systematic studies conducted on Canarian *Dysdera* have resulted in a large amount of specimen records and information on geographic distribution that is not always easily accessible. Part of this information is scattered among several publications, and many records remain unpublished. Moreover, many records of Canarian *Dysdera* only included information on locality names, sometimes difficult to identify on a map. The conversion of the locality names into georeferenced points facilitates the correct assignment and visualization of species distribution (Chapman and Wieczorek 2006). The creation of an up to date, fully georeferenced distribution database of *Dysdera* in the Canary Islands based on all available records, is a necessary step to summarize and further improve our current knowledge on this remarkable genus, and will offer an invaluable tool for future systematic, evolutionary, biogeographic, and ecological studies.

## Methods

### Sampling

Specimens referred in this database were collected during the last 45 years, mainly by members of the **GIET** (Grupo de Investigaciones Entomológicas de Tenerife) from the University of La Laguna (henceforth **ULL**), and by members of the University of Barcelona (**UB**), as well as by some external collaborators. The material used to assemble the database comes from different sources, most of the records corresponding to collection trips conducted during PhD and master thesis fieldwork, and by other research projects (see details below):

1. Specimens from PhD and master dissertations carried out at the University of La Laguna and University of Barcelona (A.L. Medina 1991, J.L. Martín 1992, M.A. Arnedo 1996, N. Macías-Hernández 2010, M. Roca-Cusachs 2016) (70%, approx. 3,200 records).
2. Specimens collected during research projects and other unpublished studies conducted by i) GIET at the University of La Laguna: LIFE-Nature Project in Teide National Park (Oromí et al. 2002), Archipiélago Chinijo (Macías et al. 2004), NetBiome Project (unpublished data); ii) National Museum of Natural History at Madrid: 1999 and 2000 in Caldera de Taburiente National Park (Domingo-Quero et al. 2003) (30%, approx. 1,380 records).

Most of the material used in the dataset is deposited in the collections of the Department of Animal Biology, Edaphology and Geology, University of La Laguna (**DZUL**), and the *Centre de Recerca de Biodiversitat Animal*, University of Barcelona (**CRBA: UB**).

Although the sampling procedure depended on each research project, it was mainly conducted by direct searching under stones and logs, beneath tree bark, scraping soil and rocky embankments, or in volcanic caves. A large amount of specimens were also collected by using pitfall traps both on the surface and in the mesovoid shallow substratum (MSS) (López and Oromí 2010). The specimens collected were later preserved in individual vials either in 70% or in 95% ethanol. Labels for each specimen included information on locality, date, and collectors, as well as the taxonomic identification. This information was digitized following the criteria and standards of the Global Biodiversity Information Facility (GBIF http://www.gbif.org), and the specimens stored at the ULL are kept in the DZUL collection, which includes type material of 29 Macaronesian species of *Dysdera* (Reboleira et al. 2012).

### Geographic distribution of *Dysdera*

A complete raw dataset of specimen-based records identified to species level by the authors was firstly assembled. All available information (i.e. number of specimens, species identification, identifier’s name, sex, locality, geographic coordinates, altitude, type of habitat, date, collector and observations) was digitized in a Microsoft Excel 2011 spreadsheet (data not shown). A second species distribution table (Suppl. material 1) was constructed using the previous raw dataset, including all localities where each species was found, the type of habitat and the altitude of each location.

GPS coordinates were converted to decimal degrees with the online coordinate converter available at http://www.asturnatura.com/sinflac/calculadora-conversiones-coordenadas.php. Old locality records without available geographic coordinates were identified and assigned by the IDECanarias online platform *Sistema de Información Territorial de Canarias* of the Canary Islands Government (http://visor.grafcan.es/visorweb/). Information about doubtful localities was requested from the collectors when possible. Records that were not georeferenced (1% of the total) were excluded from the database.

Following data entry, a data checking procedure to minimize likely data-entry errors was conducted. This included an assessment of records with the same localities for spelling errors, double-checking uncertain records (species identification, geographical coordinates, etc). Accurate spelling of scientific names and taxonomic synonyms was revised according to Arnedo (2003).

A georeferenced distribution map of each *Dysdera* species was generated using the free open source Geographic Information System program QGIS 2.12.3 (QGIS 2016).

## Results

### Database summary

The original raw database, from which all locality records were extracted, consisted of 4,595 individual records identified to species level (data not shown). All georeferenced localities where each species was collected are shown in Suppl. material 1.

### Species distribution

The distribution of each *Dysdera* species per island is summarized in Table 1. This table includes the corrected and updated species distribution regarding the last published checklist of terrestrial species from the Canary Islands (Macías-Hernández, 2010), as well as many new citations not included in the public database *Banco de Datos de Biodiversidad de Canarias* (http://www.biodiversidadcanarias.es/atlantis/common/index.jsf).

**Table 1.** Presence of each *Dysdera* species per island, indicating the corresponding number of endemic species. The troglobitic species are marked on grey. H: El Hierro, P: La Palma, G: La Gomera, T: Tenerife, C: Gran Canaria, F: Fuerteventura, L: Lanzarote.

Species	H	P	G	T	C	F	L
***Dysdera alegranzaensis*** Wunderlich, 1992							**X**
***Dysdera ambulotenta*** Ribera, Ferrández & Blasco, 1985				**X**			
***Dysdera andamanae*** Arnedo & Ribera, 1997					**X**		
***Dysdera arabisenen*** Arnedo & Ribera, 1997					**X**		
***Dysdera bandamae*** Schmidt, 1973					**X**		
***Dysdera brevisetae*** Wunderlich, 1992				**X**			
***Dysdera brevispina*** Wunderlich, 1992				**X**			
***Dysdera calderensis*** Wunderlich, 1987		**X**	**X**				
***Dysdera chioensis*** Wunderlich, 1992				**X**			
***Dysdera cribellata*** Simon, 1883				**X**			
***Dysdera crocata*** Koch, 1838	**X**	**X**	**X**	**X**	**X**		
***Dysdera curvisetae*** Wunderlich, 1992				**X**			
***Dysdera enghoffi*** Arnedo, Oromí & Ribera, 1997			**X**				
***Dysdera esquiveli*** Ribera & Blasco, 1986				**X**			
***Dysdera gibbifera*** Wunderlich, 1992				**X**			
***Dysdera gollumi*** Ribera & Arnedo, 1994				**X**			
***Dysdera gomerensis*** Strand, 1911	**X**		**X**				
***Dysdera guayota*** Arnedo & Ribera, 1999			**X**	**X**			
***Dysdera hernandezi*** Arnedo & Ribera, 1999				**X**			
***Dysdera hirguan*** Arnedo, Oromí & Ribera, 1997			**X**				
***Dysdera iguanensis*** Wunderlich, 1987				**X**	**X**		
***Dysdera insulana*** Simon, 1883				**X**	**X**		
***Dysdera labradaensis*** Wunderlich, 1992				**X**			
***Dysdera lancerotensis*** Simon, 1907						**X**	**X**
***Dysdera levipes*** Wunderlich, 1987			**X**	**X**	**X**		
***Dysdera liostethus*** Simon, 1907					**X**		
***Dysdera longa*** Wunderlich, 1992						**X**	
***Dysdera macra*** Simon, 1883				**X**			
***Dysdera madai*** Arnedo, 2007				**X**			
***Dysdera mahan*** Macías-Hernández & Arnedo, 2010						**X**	**X**
***Dysdera minutissima*** Wunderlich, 1992				**X**			
***Dysdera montanetensis*** Wunderlich, 1992				**X**			
***Dysdera nesiotes*** Simon, 1907							**X**
***Dysdera orahan*** Arnedo, Oromí & Ribera, 1997	**X**		**X**				
***Dysdera paucispinosa*** Wunderlich, 1992					**X**		
***Dysdera ramblae*** Arnedo, Oromí & Ribera, 1997			**X**				
***Dysdera ratonensis*** Wunderlich, 1992		**X**					
***Dysdera rugichelis*** Simon, 1907					**X**		
***Dysdera sanborondon*** Arnedo, Oromí & Ribera, 2000						**X**	
***Dysdera sibyllina*** Arnedo, 2007				**X**			
***Dysdera silvatica*** Schmidt, 1981	**X**	**X**	**X**				
***Dysdera simbeque*** Macías-Hernández & Arnedo, 2010							**X**
***Dysdera spinidorsum*** Wunderlich, 1992						**X**	
***Dysdera tilosensis*** Wunderlich, 1992					**X**		
***Dysdera unguimmanis*** Ribera, Ferrández & Blasco, 1985				**X**			
***Dysdera verneaui*** Simon, 1883				**X**			
***Dysdera volcania*** Ribera, Ferrández & Blasco, 1985				**X**			
***Dysdera yguanirae*** Arnedo & Ribera, 1997					**X**		
**Total single-island endemic species**	-	**1**	**3**	**19**	**8**	**3**	**3**
**Total Canarian endemic species**	**3**	**3**	**9**	**23**	**11**	**5**	**5**

**Figure 3.**
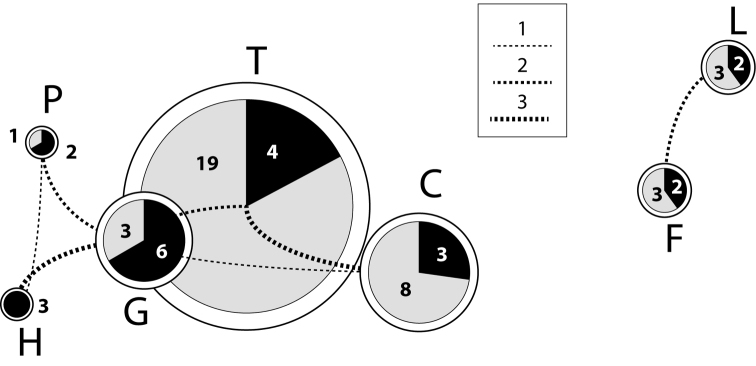
Graphical representation of the island endemisms and the species shared between islands. Pie sizes are proportional to the number of species on each island. Black sectors: number of species shared with other islands; grey sectors: proportion of local endemisms. Lines connecting pies: number of shared species between the corresponding islands, the width of the lines being proportional to the number of shared species. The disposition of pies reflects the geographical arrangement of the islands: P: La Palma, H: El Hierro, G: La Gomera, T: Tenerife, C: Gran Canaria, F: Fuerteventura, L: Lanzarote.

The proportion of endemic *Dysdera* species per island and the species shared between islands is shown graphically in Fig. 3. All distributional maps of the species are presented in the Suppl. material 2.

## Discussion

The species composition of oceanic islands is the joint result of colonization from nearby continental regions, and local speciation and extinction processes (MacArthur and Wilson 1967). Molecular phylogenetic analyses of Canarian *Dysdera* suggest that the present day diversity of the genus on the island traces back to two or three colonization events (Arnedo et al. 2001, Arnedo et al. 2007). The species on the western Canaries and the species on the eastern Canaries form distinct clades, the relationships of which remain unresolved. The last colonization event corresponds to the species *Dysdera lancerotensis*, which has its closest relatives in Morocco and colonized the eastern Canaries more recently.

Species richness in Canarian *Dysdera* is positively correlated with the area, the elevation and the habitat diversity of the islands (Arnedo and Ribera 1999a, Arnedo et al. 2000a, Cardoso et al. 2010). It shows the typical humpback relationship with age also described for other organisms inhabiting the Canary Islands (Fig. 3) (Emerson and Oromí 2005, Cardoso et al. 2010): the central islands, of intermediate age (La Gomera, Tenerife and Gran Canaria) harbour the highest number of both species (9, 23, and 11 respectively) and single island endemics. Both the youngest (La Palma and El Hierro) and the oldest (Fuerteventura and Lanzarote) islands have less species, but the proportion of island endemics is higher in the oldest islands. This pattern is interpreted as the result of high local diversification and low extinction in the central islands, high extinction rates on the oldest ones due to the habitat lost driven by erosion (Arnedo et al. 2000b, Macías-Hernández et al. 2008) and the dominant role of immigration on the youngest islands.

*Dysdera* spiders have colonized all types of terrestrial habitats in the Canaries, from the intertidal (Macías-Hernández et al. 2010) to the highest altitudes (Macías-Hernández et al. 2013). *Dysdera* seems to be especially diverse in the laurel forest. The laurel forest of Anaga and Teno in Tenerife harbour nine species each. Similarly, the laurel forest of La Gomera has seven species, and the small remnants of such forest in Gran Canaria, five species. *Dysdera* have also colonized the underground environment. The Canary Islands harbour the highest number of cave-dwelling species in the whole genus (10 out of the 16 species reported so far in the whole genus). Furthermore, *Dysdera* is the spider genus with the highest number of troglobitic species in the archipelago (Oromí et al. 2001, Oromí 2004). To date, nine troglobitic species have been found in Tenerife and one in La Palma (highlighted in grey in Table 1) (Arnedo et al. 2007). Cave-dwelling species are not restricted to lava tubes and recent prospection of the mesovoid shallow environment (MSS) in El Hierro, Tenerife and Gran Canaria have shown evidence of taxa with morphological adaptations to subterranean environments.

A striking pattern of *Dysdera* in the Canaries is the frequent co-occurrence of species in the same locality. With very few, if any, exceptions, all the species overlap distributions with at least one other species. This observation raises the question of what are the factors that promote species coexistence in Canarian *Dysdera*. Co-occurring species tend to differ in size and cheliceral shape, which in *Dysdera* has been shown to be a proxy for diet specialization: some species seem to be generalist predators while other are oniscophagous specialists (i.e. they feed preferably on woodlice) (Řezáč et al. 2008). A study combining phylogenetic information with distribution ranges in cave-dwelling *Dysdera* suggested that character displacement in phenotypic characters related to prey capture following secondary contact may explain the high levels of species range overlapping (Arnedo et al. 2007).

The conservation status of the genus *Dysdera* in the Canary Islands has not been adequately addressed yet. Some of the richest localities in terms of number of endemic *Dysdera* are protected areas included in the *Red Canaria de Espacios Naturales Protegidos* and the Natura 2000 Network. One of the main threats on the species survival is the loss or degradation of suitable habitats. This is especially true for species with very restricted distributions, or low abundances, and the very specialized species inhabiting the fragile underground environment. Previous studies conducted in several caves of the Canarian archipelago (Oromí et al. 2001), revealed high levels of severe soil pollution, sometimes favoured by the illegal use of lava tubes as house sewages. The introduction of exotic species represents an additional thread, due to the competition for resources with the endemic species. In the Macaronesian archipelagos the cosmopolitan *Dysdera crocata* has been catalogued as an invasive species (Cardoso et al. 2008, Boletín Oficial del Estado 2013) due to its negative impact on the ecosystems, and in the Azores it has been considered as the main cause of extinction for native *Dysdera* (Cardoso et al. 2010). In the Canary Islands *Dysdera crocata* is widespread, and can also be found in natural habitats (Macías-Hernández and de la Cruz in prep.), but further studies are required to determine the real impact on the native fauna. Several Canarian *Dysdera* species are currently being assessed for the IUCN (Macías-Hernández in prep.) for eventual redlisting and to guarantee the protection of declining or endangered species. Future studies focused on the conservation of the Canarian biota are needed to ensure the survival of these fragile island ecosystems.

The database and geographical maps presented in this article stand for the most updated and extensive information on the distribution of the spider genus *Dysdera* in the Canary Islands. One of the most powerful applications of this database is its use as a data-exploration tool for further ecological, biogeographical, taxonomic and conservation studies. It will facilitate the visualization of widespread and narrowly distributed species, the patterns of species coexistence, and the distribution gaps. Furthermore, the combination of georeferenced distribution data with environmental information (habitat use, vegetation, projected climate, etc.) can be used for ecological niche modelling studies (Graham et al. 2004).

## Data resources

Data package title: A geographical distribution database of the genus *Dysdera* in the Canary Islands (Araneae, Dysderidae)

Provisional doi: 10.5061/dryad.t63mn

Data files: Macías-Hernandez et al. (2016), Zookeys, Species Distribution Table

Macías-Hernandez et al. (2016), Zookeys, Species Distribution Maps

## Supplementary materials

Supplementary material 1 Supplementary Table 1 Data type: distribution data Explanation note: Distribution of each *Dysdera* species, indicating species name, locality, district, island, type of habitat, geographic coordinates, and altitude (m). This dataset is made available under the Open Database License (http://opendatacommons.org/licenses/odbl/1.0/). The Open Database License (ODbL) is a license agreement intended to allow users to freely share, modify, and use this Dataset while maintaining this same freedom for others, provided that the original source and author(s) are credited.Nuria Macías-Hernández, Salvador de la Cruz López, Marcos Roca-Cusachs, Pedro Oromí, Miquel A. Arnedo

Supplementary material 2 Supplementary Figure 1 Data type: distribution data Explanation note: Distributional map of each species of *Dysdera* (alphabetically ordered). This dataset is made available under the Open Database License (http://opendatacommons.org/licenses/odbl/1.0/). The Open Database License (ODbL) is a license agreement intended to allow users to freely share, modify, and use this Dataset while maintaining this same freedom for others, provided that the original source and author(s) are credited.Nuria Macías-Hernández, Salvador de la Cruz López, Marcos Roca-Cusachs, Pedro Oromí, Miquel A. Arnedo
