# Supplementary material for: A geographical distribution database of the genus Dysdera in the Canary Islands (Araneae, Dysderidae)
Source: Zookeys. 2016 Oct 19;(625):11–23. doi: 10.3897/zookeys.625.9847 (PMC5096360; doi:10.3897/zookeys.625.9847)
Supplement: Supplementary material 1 — Supplementary Table 1 [file zookeys-625-011-s001.pdf]

Species	Locality	District	Island	Habitat	Y	X	Altitude (m)
Dysdera alegranzaensis Wunderlich, 1992	Atalaya de Femés. Los Ajaches	Yaiza	Lanzarote	Lowland xerophytic shrub	28.919520	-13.763700	380
Dysdera alegranzaensis Wunderlich, 1992	Barranco Elvira Sánchez	Haría	Lanzarote	Thermo-sclerophyllous woodlands	29.152247	-13.502376	281
Dysdera alegranzaensis Wunderlich, 1992	Barranco Hondo del Valle	Haría	Lanzarote	Thermo-sclerophyllous woodlands	29.140800	-13.483200	125
Dysdera alegranzaensis Wunderlich, 1992	Barranco Teguereste. El Higuaral. Guatiza	Teguise	Lanzarote	Lowland xerophytic shrub	29.053280	-13.493328	111
Dysdera alegranzaensis Wunderlich, 1992	Borde de La Caldera	Alegranza	Lanzarote	Lowland xerophytic shrub	29.399593	-13.526570	242
Dysdera alegranzaensis Wunderlich, 1992	Caldera de Pedro Barba	La Graciosa	Lanzarote	Lowland xerophytic shrub	29.262813	-13.483270	24
Dysdera alegranzaensis Wunderlich, 1992	Caldera. Montaña Clara	Montaña Clara	Lanzarote	Lowland xerophytic shrub	29.298860	-13.535300	241
Dysdera alegranzaensis Wunderlich, 1992	El Faro	Alegranza	Lanzarote	Lowland xerophytic shrub	29.403350	-13.488700	5
Dysdera alegranzaensis Wunderlich, 1992	Famara	Haría	Lanzarote	Thermo-sclerophyllous woodlands	29.184360	-13.501310	347
Dysdera alegranzaensis Wunderlich, 1992	Los Ajaches	Yaiza	Lanzarote	Lowland xerophytic shrub	28.881746	-13.776582	294
Dysdera alegranzaensis Wunderlich, 1992	Máguez	Yaiza	Lanzarote	Thermo-sclerophyllous woodlands	29.154410	-13.519440	268
Dysdera alegranzaensis Wunderlich, 1992	Mirador del Río	Haría	Lanzarote	Lowland xerophytic shrub	29.211204	-13.484156	439
Dysdera alegranzaensis Wunderlich, 1992	Montaña Bermeja	La Graciosa	Lanzarote	Lowland xerophytic shrub	29.278200	-13.507390	32
Dysdera alegranzaensis Wunderlich, 1992	Montaña Blanca	Tías	Lanzarote	Lowland xerophytic shrub	28.980257	-13.638351	513
Dysdera alegranzaensis Wunderlich, 1992	Montaña Clara Caldera (ridge)	Montaña Clara	Lanzarote	Lowland xerophytic shrub	29.300601	-13.538081	189
Dysdera alegranzaensis Wunderlich, 1992	Montaña del Mojón	La Graciosa	Lanzarote	Lowland xerophytic shrub	29.242060	-13.516290	135
Dysdera alegranzaensis Wunderlich, 1992	Montaña Las Agujas	La Graciosa	Lanzarote	Lowland xerophytic shrub	29.253780	-13.504026	226
Dysdera alegranzaensis Wunderlich, 1992	Montaña Lobos. Caldera	Alegranza	Lanzarote	Lowland xerophytic shrub	29.393920	-13.501940	74
Dysdera alegranzaensis Wunderlich, 1992	Montaña Tinache	Tinajo	Lanzarote	Lowland xerophytic shrub	29.051850	-13.669850	3214
Dysdera alegranzaensis Wunderlich, 1992	Morro del Cernícalo	Alegranza	Lanzarote	Lowland xerophytic shrub	29.409820	-13.513269	25
Dysdera alegranzaensis Wunderlich, 1992	Valle de Fenauco. Los Lomos	Yaiza	Lanzarote	Lowland xerophytic shrub	28.932870	-13.773660	282
Dysdera alegranzaensis Wunderlich, 1992	Zonzamas	San Bartolomé	Lanzarote	Lowland xerophytic shrub	29.012830	-13.569950	185
Dysdera ambulotenta Ribera, Ferrández & Blasco, 1985	Cueva de Felipe Reventón	Icod de los Vinos	Tenerife	Lava tube	28.350180	-16.704638	612
Dysdera ambulotenta Ribera, Ferrández & Blasco, 1985	Cueva de Las Mechas	El Sauzal	Tenerife	Lava tube	28.438652	-16.412747	1046
Dysdera ambulotenta Ribera, Ferrández & Blasco, 1985	Cueva de los Roques. Teide National Park	La Orotava	Tenerife	Lava tube	28.236393	-16.642253	2266
Dysdera ambulotenta Ribera, Ferrández & Blasco, 1985	Cueva del Bucio. Aguamansa	La Orotava	Tenerife	Lava tube	28.360891	-16.498084	1078
Dysdera ambulotenta Ribera, Ferrández & Blasco, 1985	Cueva del Viento. Sobrado	Icod de los Vinos	Tenerife	Lava tube	28.345283	-16.698562	730
Dysdera ambulotenta Ribera, Ferrández & Blasco, 1985	Cueva Labrada	El Sauzal	Tenerife	Lava tube	28.437553	-16.413396	1048
Dysdera andamanae Arnedo & Ribera, 1997	Barranco Oscuro	Valleseco	Gran Canaria	Laurel forest	28.067250	-15.589010	767
Dysdera andamanae Arnedo & Ribera, 1997	Brezal del Palmital	Santa María de Guía	Gran Canaria	Laurel forest	28.111450	-15.601970	495
Dysdera arabisenen Arnedo & Ribera, 1997	Barranco del Andén	Valleseco	Gran Canaria	Pine forest	28.025130	-15.606750	1535

Dysdera arabisenen Arnedo & Ribera, 1997	Barranco El Draguillo	Ingenio	Gran Canaria	Lowland xerophytic shrub	27.941568	-15.429992	200
Dysdera arabisenen Arnedo & Ribera, 1997	Caldera de los Marteles	Telde	Gran Canaria	Thermo-sclerophyllous woodlands	27.952243	-15.527475	1536
Dysdera arabisenen Arnedo & Ribera, 1997	Cañada de los Alvarados (Degollada de Becerra)	Tejeda	Gran Canaria	Pine forest	27.994244	-15.594384	1553
Dysdera arabisenen Arnedo & Ribera, 1997	Cruz de Tejeda	Tejeda	Gran Canaria	Pine forest	28.000616	-15.600653	1451
Dysdera arabisenen Arnedo & Ribera, 1997	Cuevas Blancas	Valsequillo	Gran Canaria	Pine forest	27.964562	-15.546639	1699
Dysdera arabisenen Arnedo & Ribera, 1997	Cumbre de Pajonales	Tejeda	Gran Canaria	Pine forest	27.943295	-15.678000	1280
Dysdera arabisenen Arnedo & Ribera, 1997	Degollada de Becerra	Tejeda	Gran Canaria	Pine forest	27.989730	-15.592920	1565
Dysdera arabisenen Arnedo & Ribera, 1997	El Sao	Agaete	Gran Canaria	Pine forest	28.068764	-15.656990	480
Dysdera arabisenen Arnedo & Ribera, 1997	Llanos de la Pez	Tejeda	Gran Canaria	Pine forest	27.964312	-15.585547	1662
Dysdera arabisenen Arnedo & Ribera, 1997	Mirador de Moriscos	Tejeda	Gran Canaria	Pine forest	28.020976	-15.612128	1687
Dysdera arabisenen Arnedo & Ribera, 1997	Pico de las Nieves (camping place)	Vega de San Mateo	Gran Canaria	Pine forest	27.968829	-15.569527	1839
Dysdera arabisenen Arnedo & Ribera, 1997	Pinar de Tamadaba	Agaete	Gran Canaria	Pine forest	28.031840	-15.677020	1134
Dysdera arabisenen Arnedo & Ribera, 1997	Presa de Cuevas Blancas	Valsequillo	Gran Canaria	Pine forest	27.964830	-15.545050	1661
Dysdera bandamae Schmidt, 1973	Alto de Fataga	San Bartolomé de Tirajana	Gran Canaria	Pine forest	27.915237	-15.575730	941
Dysdera bandamae Schmidt, 1973	Andén Verde	Artenara	Gran Canaria	Lowland xerophytic shrub	28.035189	-15.746346	677
Dysdera bandamae Schmidt, 1973	Barranco de Guayadeque	Agüimes	Gran Canaria	Lowland xerophytic shrub	27.932980	-15.480070	550
Dysdera bandamae Schmidt, 1973	Barranco El Draguillo	Ingenio	Gran Canaria	Lowland xerophytic shrub	27.941568	-15.429992	200
Dysdera bandamae Schmidt, 1973	Barranco Oscuro	Valleseco	Gran Canaria	Laurel forest	28.067250	-15.589010	767
Dysdera bandamae Schmidt, 1973	Brezal del Palmital	Santa María de Guía	Gran Canaria	Laurel forest	28.111450	-15.601970	495
Dysdera bandamae Schmidt, 1973	Caldera de los Marteles	Telde	Gran Canaria	Thermo-sclerophyllous woodlands	27.952243	-15.527475	1536
Dysdera bandamae Schmidt, 1973	Cortijo San Gregorio. Tamaraceite	Las Palmas de GC	Gran Canaria	Thermo-sclerophyllous woodlands	28.085788	-15.491280	320
Dysdera bandamae Schmidt, 1973	Cuevas Blancas	Valsequillo	Gran Canaria	Pine forest	27.964562	-15.546639	1699
Dysdera bandamae Schmidt, 1973	Degollada de Becerra	Tejeda	Gran Canaria	Pine forest	27.989730	-15.592920	1565
Dysdera bandamae Schmidt, 1973	Degollada de Las Brujas	Mogán	Gran Canaria	Pine forest	27.940540	-15.731890	1220
Dysdera bandamae Schmidt, 1973	Degollada de Tasartico	San Nicolás de Tolentino	Gran Canaria	Lowland xerophytic shrub	27.930860	-15.791130	315
Dysdera bandamae Schmidt, 1973	El Sao	Agaete	Gran Canaria	Pine forest	28.068764	-15.656990	480
Dysdera bandamae Schmidt, 1973	Forest road to Tirma	Artenara	Gran Canaria	Lowland xerophytic shrub	28.032133	-15.755400	610
Dysdera bandamae Schmidt, 1973	Inagua	Mogán	Gran Canaria	Pine forest	27.938683	-15.708818	1161
Dysdera bandamae Schmidt, 1973	La Calderilla	Vega de San Mateo	Gran Canaria	Pine forest	27.963466	-15.553679	1771
Dysdera bandamae Schmidt, 1973	Llanos de la Pez	Tejeda	Gran Canaria	Pine forest	27.964312	-15.585547	1662
Dysdera bandamae Schmidt, 1973	Lomo de la Retamilla. Lanzarote	Valleseco	Gran Canaria	Thermo-sclerophyllous woodlands	28.028234	-15.605913	1500
Dysdera bandamae Schmidt, 1973	Los Berrazales	Agaete	Gran Canaria	Pine forest	28.068779	-15.658864	435

Dysdera bandamae Schmidt, 1973	Los Majaletes-Cazadores	Ingenio	Gran Canaria	Thermo-sclerophyllous woodlands	27.940161	-15.499200	900
Dysdera bandamae Schmidt, 1973	Montaña de Sándara	Tejeda	Gran Canaria	Pine forest	27.949398	-15.692692	1567
Dysdera bandamae Schmidt, 1973	near Cruz de San Antonio	Mogán	Gran Canaria	Pine forest	27.916234	-15.691220	900
Dysdera bandamae Schmidt, 1973	Pico de las Nieves (camping place)	Vega de San Mateo	Gran Canaria	Pine forest	27.968829	-15.569527	1839
Dysdera bandamae Schmidt, 1973	Pinar de Tamadaba	Agaete	Gran Canaria	Pine forest	28.031840	-15.677020	1134
Dysdera bandamae Schmidt, 1973	Presa de Cuevas Blancas	Valsequillo	Gran Canaria	Pine forest	27.964830	-15.545050	1661
Dysdera bandamae Schmidt, 1973	Presa de Las Niñas	Mogán	Gran Canaria	Pine forest	27.928312	-15.666048	881
Dysdera brevisetae Wunderlich, 1992	Aguas Negras	Santa Cruz de Tenerife	Tenerife	Laurel forest	28.539995	-16.225119	865
Dysdera brevisetae Wunderlich, 1992	Barranco de los Cochinos. Monte del Agua	Los Silos	Tenerife	Laurel forest	28.324550	-16.819440	950
Dysdera brevisetae Wunderlich, 1992	Cabezo del Tejo	Santa Cruz de Tenerife	Tenerife	Laurel forest	28.565090	-16.165982	800
Dysdera brevisetae Wunderlich, 1992	Camino a Ichires. Anaga	La Laguna	Tenerife	Laurel forest	28.540023	-16.231988	830
Dysdera brevisetae Wunderlich, 1992	Camino frente al Pijaral. Anaga	Santa Cruz	Tenerife	Laurel forest	28.550563	-16.185540	800
Dysdera brevisetae Wunderlich, 1992	Camino La Ensillada - Chamorga	Anaga. La Laguna	Tenerife	Laurel forest	28.556209	-16.179828	800
Dysdera brevisetae Wunderlich, 1992	Chinobre	Santa Cruz de Tenerife	Tenerife	Laurel forest	28.559298	-16.173228	888
Dysdera brevisetae Wunderlich, 1992	Cruz del Carmen (path to the restaurant)	La Laguna	Tenerife	Laurel forest	28.531925	-16.279999	945
Dysdera brevisetae Wunderlich, 1992	El Bailadero. Anaga	Santa Cruz de Tenerife	Tenerife	Laurel forest	28.550411	-16.203986	665
Dysdera brevisetae Wunderlich, 1992	El Coromoto	La Laguna	Tenerife	Urban area	28.483629	-16.328269	580
Dysdera brevisetae Wunderlich, 1992	El Moquinal	La Laguna	Tenerife	Laurel forest	28.537349	-16.309386	769
Dysdera brevisetae Wunderlich, 1992	Forest road El Batán - Cruz del Carmen	La Laguna	Tenerife	Laurel forest	28.535320	-16.296800	880
Dysdera brevisetae Wunderlich, 1992	Forest road Las Hiedras - Las Carboneras	La Laguna	Tenerife	Laurel forest	28.535600	-16.298810	950
Dysdera brevisetae Wunderlich, 1992	Ijuana forest road	Santa Cruz de Tenerife	Tenerife	Laurel forest	28.560191	-16.169190	752
Dysdera brevisetae Wunderlich, 1992	La Caldera. Aguamansa	La Orotava	Tenerife	Pine forest	28.356576	-16.499748	1199
Dysdera brevisetae Wunderlich, 1992	Monte Aguirre	Santa Cruz de Tenerife	Tenerife	Laurel forest	28.529535	-16.268309	692
Dysdera brevisetae Wunderlich, 1992	Monte de Las Mercedes	La Laguna	Tenerife	Laurel forest	28.525678	-16.287059	776
Dysdera brevisetae Wunderlich, 1992	Monte del Agua. Teno (summit)	Los Silos	Tenerife	Laurel forest	28.314265	-16.823745	880
Dysdera brevisetae Wunderlich, 1992	Near Casas de La Cumbre	Santa Cruz de Tenerife	Tenerife	Laurel forest	28.533392	-16.248577	766
Dysdera brevisetae Wunderlich, 1992	Pedro Álvarez	Tegueste	Tenerife	Laurel forest	28.521249	-16.315105	545
Dysdera brevisetae Wunderlich, 1992	Santiago del Teide	Santiago del Teide	Tenerife	Thermo-sclerophyllous woodlands	28.302480	-16.816150	943
Dysdera brevisetae Wunderlich, 1992	Vueltas de Taganana	Santa Cruz de Tenerife	Tenerife	Laurel forest	28.542439	-16.228316	855
Dysdera brevisetae Wunderlich, 1992	Zapata	La Laguna	Tenerife	Laurel forest	28.535499	-16.296200	889

Dysdera brevispina Wunderlich, 1992	Barranco de los Cochinos. Monte del Agua	Los Silos	Tenerife	Laurel forest	28.324550	-16.819440	950
Dysdera brevispina Wunderlich, 1992	Barranco de Nieto	Santa Cruz de Tenerife	Tenerife	Laurel forest	28.534070	-16.316255	793
Dysdera brevispina Wunderlich, 1992	Barranco del Chorrillo	Vilaflor	Tenerife	Pine forest	28.167211	-16.642570	1670
Dysdera brevispina Wunderlich, 1992	Barranco del Pino	Santa Úrsula	Tenerife	Pine forest	28.384151	-16.475028	1375
Dysdera brevispina Wunderlich, 1992	Cabezo del Tejo	Santa Cruz de Tenerife	Tenerife	Laurel forest	28.565090	-16.165982	805
Dysdera brevispina Wunderlich, 1992	Chinobre	Santa Cruz de Tenerife	Tenerife	Laurel forest	28.559298	-16.173228	888
Dysdera brevispina Wunderlich, 1992	Cruz del Carmen (path to the restaurant)	La Laguna	Tenerife	Laurel forest	28.531925	-16.279999	945
Dysdera brevispina Wunderlich, 1992	Cueva de Felipe Reventón	Icod de los Vinos	Tenerife	Lava tube	28.350180	-16.704638	612
Dysdera brevispina Wunderlich, 1992	Cueva del Viento. Sobrado	Icod de los Vinos	Tenerife	Lava tube	28.345283	-16.698562	730
Dysdera brevispina Wunderlich, 1992	Cueva Grande de Chío	Guía de Isora	Tenerife	Lava tube	28.250570	-16.776300	1120
Dysdera brevispina Wunderlich, 1992	El Bailadero. Anaga	Santa Cruz de Tenerife	Tenerife	Laurel forest	28.550411	-16.203986	665
Dysdera brevispina Wunderlich, 1992	El Moquinal	La Laguna	Tenerife	Laurel forest	28.537349	-16.309386	769
Dysdera brevispina Wunderlich, 1992	Forest road El Batán - Cruz del Carmen	La Laguna	Tenerife	Laurel forest	28.535320	-16.296800	880
Dysdera brevispina Wunderlich, 1992	Forest road Las Hiedras - Las Carboneras	La Laguna	Tenerife	Laurel forest	28.535600	-16.298810	950
Dysdera brevispina Wunderlich, 1992	Monte Aguirre	Santa Cruz de Tenerife	Tenerife	Laurel forest	28.529535	-16.268309	692
Dysdera brevispina Wunderlich, 1992	Pinar de Ifonche	Adeje	Tenerife	Pine forest	28.141800	-16.691400	1037
Dysdera brevispina Wunderlich, 1992	Vilaflor	Vilaflor	Tenerife	Pine forest	28.176580	-16.643480	1719
Dysdera brevispina Wunderlich, 1992	Zapata	La Laguna	Tenerife	Laurel forest	28.535499	-16.296200	889
Dysdera calderensis Wunderlich, 1987	Agua de Los Llanos	Agulo	La Gomera	Laurel forest	28.155715	-17.246339	775
Dysdera calderensis Wunderlich, 1987	Barranco Aramaqué. Near Los Aceviños	Hermigua	La Gomera	Laurel forest	28.149151	-17.220779	855
Dysdera calderensis Wunderlich, 1987	Barranco de Juel	San Sebastián	La Gomera	Laurel forest	28.151547	-17.162962	640
Dysdera calderensis Wunderlich, 1987	Barranco de los Castradores	San Sebastián	La Gomera	Midland xerophytic shrub	28.096180	-17.201420	855
Dysdera calderensis Wunderlich, 1987	Chorros de Epina	Vallehermoso	La Gomera	Laurel forest	28.167036	-17.305892	640
Dysdera calderensis Wunderlich, 1987	Cueva de los Arreboles	Fuencaliente	La Palma	Lava tube	28.493785	-17.829030	430
Dysdera calderensis Wunderlich, 1987	El Castillo	Garafía	La Palma	Pine forest	28.796360	-17.971169	501
Dysdera calderensis Wunderlich, 1987	Epina	Vallehermoso	La Gomera	Laurel forest	28.095920	-17.182060	820
Dysdera calderensis Wunderlich, 1987	Espigón Atravesado. Los Tilos	San Andrés y Sauces	La Palma	Laurel forest	28.782012	-17.816332	1111
Dysdera calderensis Wunderlich, 1987	Forest path between Barranco Higuera and Barranco San Juan	Vallehermoso	La Gomera	Laurel forest	28.193320	-17.292940	695
Dysdera calderensis Wunderlich, 1987	Forest road to Machín	Garafía	La Palma	Pine forest	28.789710	-17.896884	1267
Dysdera calderensis Wunderlich, 1987	Juan Adalid	Garafía	La Palma	Thermo-sclerophyllous woodlands	28.843639	-17.906350	296
Dysdera calderensis Wunderlich, 1987	Llano de los Caños	Mazo	La Palma	Laurel forest (degraded)	28.580523	-17.799465	955

Dysdera calderensis Wunderlich, 1987	Lomo María	El Paso	La Palma	Pine forest	28.554734	-17.861913	921
Dysdera calderensis Wunderlich, 1987	Los Tilos	San Andrés y Sauces	La Palma	Laurel forest	28.783702	-17.808408	694
Dysdera calderensis Wunderlich, 1987	Mendo	El Paso	La Palma	Pine forest	28.557572	-17.867541	781
Dysdera calderensis Wunderlich, 1987	Montaña de Las Pilas, La Mérica	Valle Gran Rey	La Gomera	Lowland xerophytic shrub	28.113296	-17.335703	801
Dysdera calderensis Wunderlich, 1987	Monte de Juan Tomé. La Laja	San Sebastián	La Gomera	Laurel forest	28.123022	-17.210137	1035
Dysdera calderensis Wunderlich, 1987	Monte del Cedro	Hermigua	La Gomera	Laurel forest	28.119283	-17.237290	1260
Dysdera calderensis Wunderlich, 1987	Pinar de Roque Faro	Garafía	La Palma	Pine forest	28.798556	-17.879149	1077
Dysdera calderensis Wunderlich, 1987	Playa de Taburiente (PNCT)	El Paso	La Palma	Lowland xerophytic shrub (Pebbled beaches)	28.709619	-17.876140	482
Dysdera calderensis Wunderlich, 1987	Puerto Santo Domingo de Garafía	Garafía	La Palma	Lowland xerophytic shrub	28.823215	-17.956515	326
Dysdera calderensis Wunderlich, 1987	Reventón Oscuro. Bosque del Cedro	Hermigua	La Gomera	Laurel forest	28.125809	-17.216616	1037
Dysdera calderensis Wunderlich, 1987	Riscos de Alojera	Vallehermoso	La Gomera	Laurel forest	28.162860	-17.318101	332
Dysdera calderensis Wunderlich, 1987	Roque Faro	Garafía	La Palma	Pine forest	28.798556	-17.879149	1076
Dysdera calderensis Wunderlich, 1987	Teselinde. Ermita de Santa Clara	Vallehermoso	La Gomera	Laurel forest	28.196300	-17.287540	727
Dysdera chioensis Wunderlich, 1992	Cueva de los Roques. Teide National Park	La Orotava	Tenerife	Lava tube	28.236393	-16.642253	2266
Dysdera chioensis Wunderlich, 1992	Cueva Grande de Chío	Guía de Isora	Tenerife	Lava tube	28.250570	-16.776300	1120
Dysdera chioensis Wunderlich, 1992	Cueva Honda de Güímar	Güímar	Tenerife	Lava tube	28.310830	-16.370410	95
Dysdera chioensis Wunderlich, 1992	Cuevas Negras, Teide National Park	La Orotava	Tenerife	Lava tube	28.258700	-16.696250	2230
Dysdera chioensis Wunderlich, 1992	Montaña Blanca. Teide National Park	La Orotava	Tenerife	Dry subalpine scrub	28.270218	-16.597251	2505
Dysdera cribellata Simon, 1883	Barranco de los Cochinos. Monte del Agua	Los Silos	Tenerife	Laurel forest	28.324550	-16.819440	950
Dysdera cribellata Simon, 1883	Cercado del Palomo. Los Majuelos	La Laguna	Tenerife	Urban area	28.459695	-16.301882	410
Dysdera cribellata Simon, 1883	Cruz del Carmen (path to the restaurant)	La Laguna	Tenerife	Laurel forest	28.531925	-16.279999	945
Dysdera cribellata Simon, 1883	Cueva de Felipe Reventón	Icod de los Vinos	Tenerife	Lava tube	28.350180	-16.704638	612
Dysdera cribellata Simon, 1883	El Moquinal	La Laguna	Tenerife	Laurel forest	28.537349	-16.309386	769
Dysdera cribellata Simon, 1883	El Pijaral. Anaga	Santa Cruz de Tenerife	Tenerife	Laurel forest	28.551966	-16.189225	790
Dysdera cribellata Simon, 1883	Forest road El Batán - Cruz del Carmen	La Laguna	Tenerife	Laurel forest	28.535320	-16.296800	880
Dysdera cribellata Simon, 1883	Forest road Las Hiedras - Las Carboneras	La Laguna	Tenerife	Laurel forest	28.535600	-16.298810	950
Dysdera cribellata Simon, 1883	Las Bodegas. Anaga	La Laguna	Tenerife	Laurel forest	28.563112	-16.159527	575
Dysdera cribellata Simon, 1883	Lomo Benijo. Anaga	Santa Cruz de Tenerife	Tenerife	Laurel forest	28.573489	-16.186380	119
Dysdera cribellata Simon, 1883	Los Barranquillos	Arafo	Tenerife	Lowland xerophytic shrub	28.352711	-16.413852	545
Dysdera cribellata Simon, 1883	Montaña Tafada. Anaga	Santa Cruz	Tenerife	Laurel forest	28.577387	-16.152415	598
Dysdera cribellata Simon, 1883	Monte Aguirre	Santa Cruz de Tenerife	Tenerife	Laurel forest	28.529535	-16.268309	692

Dysdera cribellata Simon, 1883	Monte de Las Mercedes	La Laguna	Tenerife	Laurel forest	28.525678	-16.287059	776
Dysdera cribellata Simon, 1883	Pinar de Chío	Guía de Isora	Tenerife	Pine forest	28.241833	-16.763415	1184
Dysdera cribellata Simon, 1883	Sima Robada	Santa Cruz	Tenerife	Lowland xerophytic shrub	28.529070	-16.148160	397
Dysdera cribellata Simon, 1883	Vueltas de Taganana	Santa Cruz de Tenerife	Tenerife	Laurel forest	28.542082	-16.228833	833
Dysdera crocata Koch, 1838	Aguamansa	La Orotava	Tenerife	Pine forest	28.356576	-16.499748	1200
Dysdera crocata Koch, 1838	Around Cueva Labrada	El Sauzal	Tenerife	Pine forest	28.437722	-16.411037	1058
Dysdera crocata Koch, 1838	Atalaya	Vega de San Mateo	Gran Canaria	Thermo-sclerophyllous woodlands	28.025180	-15.491259	682
Dysdera crocata Koch, 1838	Bajamar	La Laguna	Tenerife	Lowland xerophytic shrub	28.552515	-16.345881	25
Dysdera crocata Koch, 1838	Barranco de Azuaje	Firgas	Gran Canaria	Thermo-sclerophyllous woodlands	28.108020	-15.570963	270
Dysdera crocata Koch, 1838	Barranco de Bensa	Santa Úrsula	Tenerife	Pine forest	28.396940	-16.457810	1230
Dysdera crocata Koch, 1838	Barranco de Ruiz	Los Realejos	Tenerife	Thermo-sclerophyllous woodlands	28.386729	-16.621133	137
Dysdera crocata Koch, 1838	Barranco Oscuro	Valleseco	Gran Canaria	Laurel forest	28.067250	-15.589010	767
Dysdera crocata Koch, 1838	Barranquillo de Las Mimbreras	San Mateo	Gran Canaria	Thermo-sclerophyllous woodlands	28.001499	-15.574660	1257
Dysdera crocata Koch, 1838	Brezal del Palmital	Santa María de Guía	Gran Canaria	Laurel forest	28.111450	-15.601970	495
Dysdera crocata Koch, 1838	Camino Ermita del Pilar	El Paso	La Palma	Pine forest	28.638020	-17.830390	1069
Dysdera crocata Koch, 1838	Carretera La Sabina - Malpaís de Mazo	Mazo	La Palma	Midland xerophytic shrub	28.581224	-17.785256	596
Dysdera crocata Koch, 1838	Cortijo San Gregorio. Tamaraceite	Las Palmas de GC	Gran Canaria	Thermo-sclerophyllous woodlands	28.085788	-15.491280	320
Dysdera crocata Koch, 1838	Cruz del Carmen (path to the restaurant)	La Laguna	Tenerife	Laurel forest	28.531925	-16.279999	945
Dysdera crocata Koch, 1838	Cubo de la Galga	Puntallana	La Palma	Laurel forest	28.766653	-17.771190	349
Dysdera crocata Koch, 1838	Cueva de Felipe Reventón	Icod de los Vinos	Tenerife	Lava tube	28.350180	-16.704638	612
Dysdera crocata Koch, 1838	Cueva del Bucio. Aguamansa	La Orotava	Tenerife	Lava tube	28.360891	-16.498084	1078
Dysdera crocata Koch, 1838	Cueva del Salto de Tigalate	Mazo	La Palma	Lava tube	28.534501	-17.793949	72
Dysdera crocata Koch, 1838	Cueva del Sobrado (outside)	Icod de los Vinos	Tenerife	Pine forest	28.343586	-16.698580	759
Dysdera crocata Koch, 1838	Cumbre Bolico	Santiago del Teide	Tenerife	Laurel forest	28.314186	-16.826940	1191
Dysdera crocata Koch, 1838	Cumbre Nueva	El Paso	La Palma	Laurel forest	28.618402	-17.833660	1471
Dysdera crocata Koch, 1838	Cumbre Vieja	El Paso	La Palma	Pine forest	28.581239	-17.835590	1894
Dysdera crocata Koch, 1838	El Aderno, over Buenavista	Buenavista del Norte	Tenerife	Thermo-sclerophyllous woodlands	28.358258	-16.864456	200
Dysdera crocata Koch, 1838	El Coromoto	La Laguna	Tenerife	Urban area	28.483629	-16.328269	580
Dysdera crocata Koch, 1838	El Lagar - La Montañeta	Icod de los Vinos	Tenerife	Pine forest	28.319689	-16.725221	1343
Dysdera crocata Koch, 1838	El Lagar. Llano del Hospital forest road	La Guancha	Tenerife	Pine forest	28.335544	-16.657566	1065
Dysdera crocata Koch, 1838	El Montillo	La Matanza	Tenerife	Lowland xerophytic shrub	28.442525	-16.454611	500
Dysdera crocata Koch, 1838	El Moquinal	La Laguna	Tenerife	Laurel forest	28.537349	-16.309386	769

Dysdera crocata Koch, 1838	El Retamar	Los Llanos de Aridane	La Palma	Urban area	28.661615	-17.896299	494
Dysdera crocata Koch, 1838	El Rodeo (close to the airport)	La Laguna	Tenerife	Grassland	28.482258	-16.354571	639
Dysdera crocata Koch, 1838	El Roquillo. Valle Guerra	La Laguna	Tenerife	Lowland xerophytic shrub	28.534464	-16.396923	35
Dysdera crocata Koch, 1838	Erjos	El Tanque	Tenerife	Thermo-sclerophyllous woodlands	28.328956	-16.806871	973
Dysdera crocata Koch, 1838	Facultad de Biología	La Laguna	Tenerife	Urban area	28.480196	-16.320989	566
Dysdera crocata Koch, 1838	Fajana de Barlovento	Barlovento	La Palma	Lowland xerophytic shrub	28.842108	-17.788904	24
Dysdera crocata Koch, 1838	Finca en Aguamansa	La Orotava	Tenerife	Pine forest	28.364207	-16.496885	1024
Dysdera crocata Koch, 1838	Fontanales	Moya	Gran Canaria	Thermo-sclerophyllous woodlands	28.055530	-15.608730	1050
Dysdera crocata Koch, 1838	Forest road Las Hiedras - Las Carboneras	La Laguna	Tenerife	Laurel forest	28.535600	-16.298810	950
Dysdera crocata Koch, 1838	Forest road to Benijos	Los Realejos	Tenerife	Pine forest	28.338981	-16.547893	1292
Dysdera crocata Koch, 1838	Forest road to las Llanadas. Zona Recreativa Chanajigas	Los Realejos	Tenerife	Pine forest	28.343650	-16.584541	1295
Dysdera crocata Koch, 1838	Forest road to Los Órganos (beyond los Sanguinos Forest)	Santa Úrsula	Tenerife	Pine forest	28.362888	-16.483766	1295
Dysdera crocata Koch, 1838	Fuente Olén	Santa Cruz de La Palma	La Palma	Pine forest	28.730912	-17.815302	1760
Dysdera crocata Koch, 1838	Geneto	La Laguna	Tenerife	Urban area	28.460830	-16.316071	505
Dysdera crocata Koch, 1838	Interián	Los Silos	Tenerife	Thermo-sclerophyllous woodlands	28.360674	-16.802215	302
Dysdera crocata Koch, 1838	La Caldera, Aguamansa, Lomo Colorado forest road	La Orotava	Tenerife	Pine forest	28.359658	-16.501015	1141
Dysdera crocata Koch, 1838	La Caldera. Aguamansa	La Orotava	Tenerife	Pine forest	28.356576	-16.499748	1199
Dysdera crocata Koch, 1838	La Cruz Chica	La Laguna	Tenerife	Urban area	28.488724	-16.363194	630
Dysdera crocata Koch, 1838	La Cumbrecita	El Paso	La Palma	Pine forest	28.693749	-17.859306	1557
Dysdera crocata Koch, 1838	La Gallega	Santa Cruz	Tenerife	Urban area	28.426947	-16.321910	370
Dysdera crocata Koch, 1838	La Laguna. Valleseco	Valleseco	Gran Canaria	Thermo-sclerophyllous woodlands (degraded)	28.064452	-15.563843	870
Dysdera crocata Koch, 1838	La Lechucilla. Montañón Negro	Vega de San Mateo	Gran Canaria	Thermo-sclerophyllous woodlands (degraded)	27.995782	-15.545465	1025
Dysdera crocata Koch, 1838	La Matanza	La Matanza de Acentejo	Tenerife	Urban area	28.440823	-16.442310	675
Dysdera crocata Koch, 1838	La Montañeta	Garachico	Tenerife	Pine forest	28.332726	-16.757315	1037
Dysdera crocata Koch, 1838	La Quebrada. Timagada	Tejeda	Gran Canaria	Thermo-sclerophyllous woodlands	27.973311	-15.620715	1245
Dysdera crocata Koch, 1838	La Resbala	La Orotava	Tenerife	Laurel forest	28.401634	-16.496808	725
Dysdera crocata Koch, 1838	La Rosa	Mazo	La Palma	Midland xerophytic shrub	28.617873	-17.783733	543
Dysdera crocata Koch, 1838	La Rosa. La Corujera	Santa Úrsula	Tenerife	Thermo-sclerophyllous woodlands	28.396940	-16.457810	1226
Dysdera crocata Koch, 1838	Laguna de Barlovento	Barlovento	La Palma	Laurel forest	28.808894	-17.803548	734
Dysdera crocata Koch, 1838	Las Hayas picnic place	Icod de los Vinos	Tenerife	Pine forest	28.336323	-16.674864	973

Dysdera crocata Koch, 1838	Las Lagunetas	El Rosario	Tenerife	Pine forest	28.418535	-16.410090	1400
Dysdera crocata Koch, 1838	Las Mercedes	La Laguna	Tenerife	Laurel forest	28.525678	-16.287059	759
Dysdera crocata Koch, 1838	Las Palomas Nature Reserve	La Victoria de Acentejo	Tenerife	Thermo-sclerophyllous woodlands	28.401807	-16.457699	1074
Dysdera crocata Koch, 1838	Las Piedras	El Paso	La Palma	Dry subalpine scrub	28.650503	-17.863845	785
Dysdera crocata Koch, 1838	Llano de los Caños	Mazo	La Palma	Laurel forest (degraded)	28.580523	-17.799465	955
Dysdera crocata Koch, 1838	Lomo del Montijo	El Escobonal	Tenerife	Lowland xerophytic shrub	28.267749	-16.425705	561
Dysdera crocata Koch, 1838	Lomo Redondo (Tigaiga y Ruiz)	Los Realejos	Tenerife	Laurel forest	28.368928	-16.623679	725
Dysdera crocata Koch, 1838	Los Gigantes	Santiago del Teide	Tenerife	Urban area	28.239102	-16.836000	124
Dysdera crocata Koch, 1838	Los LLanos	Santiago del Teide	Tenerife	Thermo-sclerophyllous woodlands	28.302483	-16.816150	945
Dysdera crocata Koch, 1838	Los Rodeos	La Laguna	Tenerife	Grassland	28.482863	-16.349497	640
Dysdera crocata Koch, 1838	Los Rodeos Airport	La Laguna	Tenerife	Grassland	28.482863	-16.349497	620
Dysdera crocata Koch, 1838	Los Tilos	San Andrés y Sauces	La Palma	Laurel forest	28.783702	-17.808408	694
Dysdera crocata Koch, 1838	Mesa Mota	La Laguna	Tenerife	Thermo-sclerophyllous woodlands	28.508352	-16.318109	722
Dysdera crocata Koch, 1838	Montaña Cabreja	Vega de San Mateo	Gran Canaria	Thermo-sclerophyllous woodlands	28.010872	-15.539417	1005
Dysdera crocata Koch, 1838	Montaña de Firgas	Firgas	Gran Canaria	Thermo-sclerophyllous woodlands	28.096288	-15.563653	650
Dysdera crocata Koch, 1838	Montaña del Dinero	Agulo	La Gomera	Laurel forest	28.159433	-17.243576	1028
Dysdera crocata Koch, 1838	Montaña Hoya de la Vaca. Jinama	Valverde	El Hierro	Pasture	27.771712	-17.981270	1200
Dysdera crocata Koch, 1838	Monte de Santa Úrsula. La Corujera	Santa Úrsula	Tenerife	Laurel forest	28.403179	-16.486186	880
Dysdera crocata Koch, 1838	Monte Pájaro. Chinyero	Garachico	Tenerife	Pine forest	28.354725	-16.769307	678
Dysdera crocata Koch, 1838	Nogales	San Andrés y Sauces	La Palma	Lowland xerophytic shrub	28.761166	-17.778518	523
Dysdera crocata Koch, 1838	Ofra	Santa Cruz de Tenerife	Tenerife	Urban area	28.450664	-16.289170	274
Dysdera crocata Koch, 1838	Palo Blanco	Los Realejos	Tenerife	Laurel forest	28.357941	-16.586559	869
Dysdera crocata Koch, 1838	Pared Vieja	Breña Alta	La Palma	Laurel forest	28.618117	-17.823325	1211
Dysdera crocata Koch, 1838	Pedro Álvarez	Tegueste	Tenerife	Laurel forest	28.521249	-16.315105	545
Dysdera crocata Koch, 1838	Pine forest over Cueva del Sobrado	Icod de los Vinos	Tenerife	Pine forest	28.347052	-16.697110	727
Dysdera crocata Koch, 1838	Pinoleris	La Orotava	Tenerife	Pine forest	28.400377	-16.496257	761
Dysdera crocata Koch, 1838	Pinos de Gáldar	Moya	Gran Canaria	Pine forest	28.039485	-15.616292	1451
Dysdera crocata Koch, 1838	Playa de La Aldea	San Nicolás de Tolentino	Gran Canaria	Lowland xerophytic shrub (Pebbled beaches)	28.001468	-15.818420	2
Dysdera crocata Koch, 1838	Playa Paraíso (10–50 m)	Adeje	Tenerife	Lowland xerophytic shrub (Pebbled beaches)	28.122994	-16.775880	16
Dysdera crocata Koch, 1838	Presa de Las Niñas	Mogán	Gran Canaria	Pine forest	27.928312	-15.666048	881
Dysdera crocata Koch, 1838	Punta del Hidalgo	La Laguna	Tenerife	Lowland xerophytic shrub	28.568108	-16.317760	86

Dysdera crocata Koch, 1838	Rambla de Castro	Los Realejos	Tenerife	Thermo-sclerophyllous woodlands	28.395687	-16.589005	128
Dysdera crocata Koch, 1838	San Diego	La Laguna	Tenerife	Urban area	28.500670	-16.324268	563
Dysdera crocata Koch, 1838	San Isidro. Utiaca	Teror	Gran Canaria	Thermo-sclerophyllous woodlands	28.028207	-15.563110	1010
Dysdera crocata Koch, 1838	San Marcos (near the cave)	Icod de los Vinos	Tenerife	Thermo-sclerophyllous woodlands	28.376460	-16.723190	81
Dysdera crocata Koch, 1838	San Pedro	Breña Alta	La Palma	Urban area	28.618117	-17.823325	1212
Dysdera crocata Koch, 1838	Santa Lucía	Santa Lucía de Tirajana	Gran Canaria	Urban area	27.915213	-15.536957	735
Dysdera crocata Koch, 1838	Santa Úrsula	Santa Úrsula	Tenerife	Pine forest	28.366470	-16.482120	1315
Dysdera crocata Koch, 1838	Supra Valsendero	Valleseco	Gran Canaria	Laurel forest	28.036154	-15.590565	997
Dysdera crocata Koch, 1838	Tabaiba	El Rosario	Tenerife	Xerophytic scrubs	28.407689	-16.328955	230
Dysdera crocata Koch, 1838	Tajuya	El Paso	La Palma	Lowland xerophytic shrub	28.635843	-17.889912	519
Dysdera crocata Koch, 1838	Teno Alto	Buenavista	Tenerife	Laurel forest	28.335996	-16.865760	861
Dysdera crocata Koch, 1838	Tilos de Moya	Moya	Gran Canaria	Laurel forest	28.088752	-15.593203	529
Dysdera crocata Koch, 1838	Valle de Honduras. Bajamar	La Laguna	Tenerife	Lowland xerophytic shrub	28.549666	-16.336482	215
Dysdera crocata Koch, 1838	Valleseco-Tejeda Carretera	Tejeda	Gran Canaria	Pine forest	28.017043	-15.582465	1415
Dysdera crocata Koch, 1838	Veneguera	Mogán	Gran Canaria	Urban area	27.906034	-15.730806	285
Dysdera crocata Koch, 1838	Vera del Risco Blanco (Tigaiga y Ruiz)	Los Realejos	Tenerife	Thermo-sclerophyllous woodlands	28.378286	-16.597801	579
Dysdera crocata Koch, 1838	Virgen del Pino	El Paso	La Palma	Pine forest	28.662125	-17.840464	911
Dysdera curvisetae Wunderlich, 1992	El Médano (beach)	Granadilla	Tenerife	Lowland xerophytic shrub (Dunes)	28.033022	-16.540247	1
Dysdera curvisetae Wunderlich, 1992	Playa del Barranco del Natero	Buenavista	Tenerife	Lowland xerophytic shrub (Pebbled beaches)	28.288321	-16.861860	2
Dysdera curvisetae Wunderlich, 1992	Playa Pachila. Chinamada	Santa Cruz	Tenerife	Lowland xerophytic shrub (Pebbled beaches)	28.570148	-16.290357	4
Dysdera enghoffi Arnedo, Oromí & Ribera, 1997	Agua de Los Llanos	Agulo	La Gomera	Laurel forest	28.142610	-17.246810	775
Dysdera enghoffi Arnedo, Oromí & Ribera, 1997	Barranco de Majona	San Sebastián	La Gomera	Lowland xerophytic shrub	28.151830	-17.139235	88
Dysdera enghoffi Arnedo, Oromí & Ribera, 1997	Barranco de Matarnos. Monte del Cedro	Hermigua	La Gomera	Laurel forest	28.125040	-17.242183	1264
Dysdera enghoffi Arnedo, Oromí & Ribera, 1997	Bosque del Cedro	Hermigua	La Gomera	Laurel forest	28.137044	-17.222569	1113
Dysdera enghoffi Arnedo, Oromí & Ribera, 1997	Campamento Viejo. Monte del Cedro	Hermigua	La Gomera	Laurel forest	28.119758	-17.225285	994
Dysdera enghoffi Arnedo, Oromí & Ribera, 1997	Monte del Cedro	Hermigua	La Gomera	Laurel forest	28.119283	-17.237290	1260
Dysdera enghoffi Arnedo, Oromí & Ribera, 1997	Pinar del Infante	Vallehermoso	La Gomera	Pine forest	28.130794	-17.282267	1092
Dysdera esquiveli Ribera & Blasco, 1986	Barranco de los Cochinos. Monte del Agua	Los Silos	Tenerife	Laurel forest	28.324550	-16.819440	950
Dysdera esquiveli Ribera & Blasco, 1986	Cueva de Felipe Reventón	Icod de los Vinos	Tenerife	Lava tube	28.350180	-16.704638	612
Dysdera esquiveli Ribera & Blasco, 1986	Cueva del Bucio. Aguamansa	La Orotava	Tenerife	Lava tube	28.360891	-16.498084	1078
Dysdera esquiveli Ribera & Blasco, 1986	Cueva del Viento. Sobrado	Icod de los Vinos	Tenerife	Lava tube	28.345283	-16.698562	730

Dysdera esquiveli Ribera & Blasco, 1986	Cueva Labrada	El Sauzal	Tenerife	Lava tube	28.437553	-16.413396	1698
Dysdera gibbifera Wunderlich, 1992	Barranco de los Cochinos. Monte del Agua	Los Silos	Tenerife	Laurel forest	28.324550	-16.819440	950
Dysdera gibbifera Wunderlich, 1992	Cueva de Felipe Reventón	Icod de los Vinos	Tenerife	Lava tube	28.350180	-16.704638	612
Dysdera gibbifera Wunderlich, 1992	El Bailadero. Anaga	Santa Cruz de Tenerife	Tenerife	Laurel forest	28.550411	-16.203986	665
Dysdera gibbifera Wunderlich, 1992	Hoya de Ijuana. Anaga	Santa Cruz de Tenerife	Tenerife	Laurel forest	28.560191	-16.169190	752
Dysdera gollumi Ribera & Arnedo, 1994	Cueva de los Roques. Teide National Park	La Orotava	Tenerife	Lava tube	28.236393	-16.642253	2266
Dysdera gomerensis Strand, 1911	Arure to Las Hayas road	Valle Gran Rey	La Gomera	Midland xerophytic shrub	28.129606	-17.300846	935
Dysdera gomerensis Strand, 1911	Barranco de Juel	San Sebastián	La Gomera	Laurel forest	28.151547	-17.162962	640
Dysdera gomerensis Strand, 1911	Barranco de los Castradores	San Sebastián	La Gomera	Midland xerophytic shrub	28.096180	-17.201420	855
Dysdera gomerensis Strand, 1911	Barranco de Majona	San Sebastián	La Gomera	Lowland xerophytic shrub	28.151830	-17.139235	88
Dysdera gomerensis Strand, 1911	Barranco de Matarnos. Monte del Cedro	Hermigua	La Gomera	Laurel forest	28.125040	-17.242183	1264
Dysdera gomerensis Strand, 1911	Barranco de Paijén	San Sebastián	La Gomera	Lowland xerophytic shrub	28.088450	-17.201420	920
Dysdera gomerensis Strand, 1911	Binto Forest road	Frontera	El Hierro	Pine forest	27.729061	-18.084246	1250
Dysdera gomerensis Strand, 1911	Cañada de Jorge	Valle Gran Rey	La Gomera	Laurel forest	28.147416	-17.291071	1065
Dysdera gomerensis Strand, 1911	Carretera Cumbre, close to Mora Gaspar	Vallehermoso	La Gomera	Laurel forest	28.146140	-17.262784	706
Dysdera gomerensis Strand, 1911	Carretera Juego de Bolas - Laguna Grande	Agulo	La Gomera	Laurel forest	28.129925	-17.254707	1186
Dysdera gomerensis Strand, 1911	Charco Manso	Valverde	El Hierro	Xerophytic scrubs	27.848201	-17.925035	18
Dysdera gomerensis Strand, 1911	Chorros de Epina	Vallehermoso	La Gomera	Laurel forest	28.167036	-17.305892	640
Dysdera gomerensis Strand, 1911	Cueva de la Curva	El Pinar	El Hierro	Lava tube	27.692348	-17.972877	621
Dysdera gomerensis Strand, 1911	Cueva de Mauricio	Frontera	El Hierro	Lava tube	27.739130	-18.066850	913
Dysdera gomerensis Strand, 1911	Cueva del Hoyo	Frontera	El Hierro	Lava tube	27.753511	-18.002527	366
Dysdera gomerensis Strand, 1911	Cueva del Mocán	Frontera	El Hierro	Lava tube	27.717959	-18.005370	1245
Dysdera gomerensis Strand, 1911	El Brezal	Frontera	El Hierro	Laurel forest	27.732629	-18.028909	1067
Dysdera gomerensis Strand, 1911	El Cepo	Agulo	La Gomera	Thermo-sclerophyllous woodlands (degraded)	28.191769	-17.216818	650
Dysdera gomerensis Strand, 1911	El Fayal	Frontera	El Hierro	Laurel forest	27.733435	-17.996380	1316
Dysdera gomerensis Strand, 1911	El Golfo	Frontera	El Hierro	Laurel forest	27.743924	-18.017786	508
Dysdera gomerensis Strand, 1911	El Sabinar	Frontera	El Hierro	Thermo-sclerophyllous woodlands	27.751746	-18.129598	517
Dysdera gomerensis Strand, 1911	Ermita de Las Nieves	San Sebastián	La Gomera	Pine forest (planted)	28.101134	-17.202401	1120
Dysdera gomerensis Strand, 1911	Ermita del Santo	Valle Gran Rey	La Gomera	Midland xerophytic shrub	28.131020	-17.322730	800
Dysdera gomerensis Strand, 1911	Forest path between Barranco Higuera and Barranco San Juan	Vallehermoso	La Gomera	Laurel forest	28.193320	-17.292940	695
Dysdera gomerensis Strand, 1911	Forest road to El Derrabado	Frontera	El Hierro	Laurel forest	27.740804	-18.053080	919

Dysdera gomerensis Strand, 1911	Forest road to El Garoé	Valverde	El Hierro	Pine forest (Pinus radiata)	27.781744	-17.951391	1036
Dysdera gomerensis Strand, 1911	Forest road to El Mercader	El Pinar	El Hierro	Pine forest	27.712944	-18.022175	1075
Dysdera gomerensis Strand, 1911	Frontera Forest House	Frontera	El Hierro	Laurel forest	27.737593	-18.022747	774
Dysdera gomerensis Strand, 1911	Fuente la Llanía	Valverde	El Hierro	Laurel forest	27.736430	-17.996938	1342
Dysdera gomerensis Strand, 1911	Fuente Mencáfite	El Golfo	El Hierro	Laurel forest	27.735497	-18.085804	925
Dysdera gomerensis Strand, 1911	Hoya del Pino	Frontera	El Hierro	Pine forest	27.737154	-18.046305	1000
Dysdera gomerensis Strand, 1911	Juaclo de las Moleras	Frontera	El Hierro	Thermo-sclerophyllous woodlands	27.732883	-18.140708	449
Dysdera gomerensis Strand, 1911	La Asomadita (Chipude forest road)	Vallehermoso	La Gomera	Laurel forest	28.111600	-17.264800	1255
Dysdera gomerensis Strand, 1911	La Campana	Hermigua	La Gomera	Laurel forest	28.158055	-17.160623	730
Dysdera gomerensis Strand, 1911	La Dehesa	Frontera	El Hierro	Thermo-sclerophyllous woodlands	27.738237	-18.136543	482
Dysdera gomerensis Strand, 1911	Las Paredes	Vallehermoso	La Gomera	Pine forest (planted)	28.098820	-17.249420	1360
Dysdera gomerensis Strand, 1911	Llanos de Crispín	Vallehermoso	La Gomera	Laurel forest	28.123806	-17.266567	1201
Dysdera gomerensis Strand, 1911	Los Noruegos	Hermigua	La Gomera	Laurel forest	28.106905	-17.233323	1360
Dysdera gomerensis Strand, 1911	Mirador de Alojera	Valle Gran Rey	La Gomera	Laurel forest	28.149780	-17.308130	990
Dysdera gomerensis Strand, 1911	Mirador de Bascos	Frontera	El Hierro	Thermo-sclerophyllous woodlands	27.754861	-18.118218	658
Dysdera gomerensis Strand, 1911	Mirador de Jinama	Valverde	El Hierro	Laurel forest (degraded)	27.762755	-17.980418	1235
Dysdera gomerensis Strand, 1911	Mirador de Las Playas	Frontera	El Hierro	Pine forest	27.731970	-17.972127	1053
Dysdera gomerensis Strand, 1911	Montaña Caldereta	Frontera	El Hierro	Laurel forest	27.743623	-18.016548	527
Dysdera gomerensis Strand, 1911	Montaña de Fara	Valverde	El Hierro	Laurel forest	27.795267	-17.950103	905
Dysdera gomerensis Strand, 1911	Montaña de Las Pilas, La Mérica	Valle Gran Rey	La Gomera	Lowland xerophytic shrub	28.113283	-17.335701	800
Dysdera gomerensis Strand, 1911	Montaña del Dinero	Agulo	La Gomera	Laurel forest	28.159433	-17.243576	1028
Dysdera gomerensis Strand, 1911	Montaña La Asomada (Laguna Grande - Juego de Bolas)	Vallehermoso	La Gomera	Laurel forest	28.135646	-17.257454	1122
Dysdera gomerensis Strand, 1911	Monte del Cedro	Hermigua	La Gomera	Laurel forest	28.119283	-17.237290	1260
Dysdera gomerensis Strand, 1911	Ojila	San Sebastián	La Gomera	Laurel forest	28.118032	-17.211050	802
Dysdera gomerensis Strand, 1911	Pajarito	Hermigua	La Gomera	Laurel forest	28.108856	-17.241604	1339
Dysdera gomerensis Strand, 1911	Pico Pedraje. El Mocanal	Valverde	El Hierro	Thermo-sclerophyllous woodlands	27.803692	-17.946575	969
Dysdera gomerensis Strand, 1911	Pie del Roque Cano	Vallehermoso	La Gomera	Laurel forest	28.184542	-17.256932	280
Dysdera gomerensis Strand, 1911	Pinar del Infante	Vallehermoso	La Gomera	Pine forest	28.130794	-17.282267	1092
Dysdera gomerensis Strand, 1911	Playa Arenas Blancas	Frontera	El Hierro	Sand dunes	27.767470	-18.122826	10
Dysdera gomerensis Strand, 1911	Playa de Hermigua	Hermigua	La Gomera	Lowland xerophytic shrub (Pebbled beaches)	28.176240	-17.175953	124
Dysdera gomerensis Strand, 1911	Punta Arenas Blancas	Frontera	El Hierro	Lowland xerophytic shrub (Dunes)	27.767373	-18.122125	5
Dysdera gomerensis Strand, 1911	Reventón Oscuro. Monte del Cedro	Hermigua	La Gomera	Laurel forest	28.125809	-17.216616	1037

Dysdera gomerensis Strand, 1911	Roques de Salmor	Valverde	El Hierro	Xerophytic scrubs	27.823454	-17.996024	95
Dysdera gomerensis Strand, 1911	Sima de las Palomas	Frontera	El Hierro	Volcanic pit	27.737870	-18.065700	1015
Dysdera gomerensis Strand, 1911	Tapagache	Vallehermoso	La Gomera	Laurel forest	28.084130	-17.289020	1134
Dysdera gomerensis Strand, 1911	Teselinde. Ermita de Santa Clara	Vallehermoso	La Gomera	Laurel forest	28.196300	-17.287540	727
Dysdera gomerensis Strand, 1911	Tiñor	Valverde	El Hierro	Pine forest	27.785991	-17.937508	1007
Dysdera gomerensis Strand, 1911	Trail to Enchereda	San Sebastián	La Gomera	Laurel forest	28.136650	-17.179063	981
Dysdera gomerensis Strand, 1911	Túnel de Agulo (Las Rosas side)	Agulo	La Gomera	Thermo-sclerophyllous woodlands	28.192455	-17.198191	250
Dysdera gomerensis Strand, 1911	Ventejís. Tiñor	Valverde	El Hierro	Pine forest	27.785991	-17.937508	1008
Dysdera guayota Arnedo & Ribera, 1999	Barranco de los Cochinos. Monte del Agua	Los Silos	Tenerife	Laurel forest	28.324550	-16.819440	950
Dysdera guayota Arnedo & Ribera, 1999	Boca Tauce. Teide National Park	Adeje	Tenerife	Dry subalpine scrub	28.211942	-16.681180	2040
Dysdera guayota Arnedo & Ribera, 1999	Forest road to Madre del Agua- Barranco del Río	Vilaflor	Tenerife	Pine forest	28.177310	-16.601628	1710
Dysdera guayota Arnedo & Ribera, 1999	La Escalona	Adeje	Tenerife	Lowland xerophytic shrub	28.116650	-16.673350	985
Dysdera guayota Arnedo & Ribera, 1999	Las Lajas picnic place	Vilaflor	Tenerife	Pine forest	28.190338	-16.669163	2050
Dysdera guayota Arnedo & Ribera, 1999	Los Cristianos	Arona	Tenerife	Urban area	28.047686	-16.704829	46
Dysdera guayota Arnedo & Ribera, 1999	Montaña de Las Pilas, La Mérica	Valle Gran Rey	La Gomera	Lowland xerophytic shrub	28.113296	-17.335703	801
Dysdera guayota Arnedo & Ribera, 1999	Montaña Las Lajas (beyond Boca Tauce)	Adeje	Tenerife	Pine forest	28.141800	-16.691400	1038
Dysdera guayota Arnedo & Ribera, 1999	Playa de Avalo	San Sebastián	La Gomera	Lowland xerophytic shrub (Pebbled beaches)	28.114684	-17.113168	34
Dysdera guayota Arnedo & Ribera, 1999	Roque del Conde	Adeje	Tenerife	Xerophytic scrubs	28.093144	-16.698890	525
Dysdera guayota Arnedo & Ribera, 1999	Vilaflor	Vilaflor	Tenerife	Pine forest	28.176580	-16.643480	1719
Dysdera hernandezi Arnedo & Ribera, 1999	Cueva de la Puerta	Santa Cruz de Tenerife	Tenerife	Lava tube	28.393240	-16.344310	118
Dysdera hernandezi Arnedo & Ribera, 1999	Cueva Labrada	El Sauzal	Tenerife	Lava tube	28.437553	-16.413396	1698
Dysdera hirguan Arnedo, Oromí & Ribera, 1997	Bosque del Cedro	Hermigua	La Gomera	Laurel forest	28.137044	-17.222569	1113
Dysdera hirguan Arnedo, Oromí & Ribera, 1997	Reventón Oscuro, Monte del Cedro	Hermigua	La Gomera	Laurel forest	28.125809	-17.216616	1037
Dysdera iguanensis Wunderlich, 1987	Barranco de Ijuana	Santa Cruz de Tenerife	Tenerife	Laurel forest	28.550260	-16.143894	263
Dysdera iguanensis Wunderlich, 1987	Barranco de los Cochinos. Monte del Agua	Los Silos	Tenerife	Laurel forest	28.324550	-16.819440	950
Dysdera iguanensis Wunderlich, 1987	Barranco de Nieto	Santa Cruz de Tenerife	Tenerife	Laurel forest	28.534070	-16.316255	793
Dysdera iguanensis Wunderlich, 1987	Cabezo del Tejo	Santa Cruz de Tenerife	Tenerife	Laurel forest	28.562022	-16.171397	805
Dysdera iguanensis Wunderlich, 1987	Cabezo del Tejo	Santa Cruz de Tenerife	Tenerife	Laurel forest	28.565090	-16.165982	800
Dysdera iguanensis Wunderlich, 1987	Cruz del Carmen (path to the restaurant)	La Laguna	Tenerife	Laurel forest	28.531925	-16.279999	945
Dysdera iguanensis Wunderlich, 1987	Degollada de Bicora. Anaga	Santa Cruz de Tenerife	Tenerife	Laurel forest	28.546166	-16.216339	718

Dysdera iguanensis Wunderlich, 1987	El Bailadero. Anaga	Santa Cruz de Tenerife	Tenerife	Laurel forest	28.550411	-16.203986	665
Dysdera iguanensis Wunderlich, 1987	El Pijaral. Anaga	Santa Cruz de Tenerife	Tenerife	Laurel forest	28.551966	-16.189225	790
Dysdera iguanensis Wunderlich, 1987	Forest road Las Hiedras - Las Carboneras	La Laguna	Tenerife	Laurel forest	28.535600	-16.298810	950
Dysdera iguanensis Wunderlich, 1987	Hoya de Ijuana. Anaga	Santa Cruz de Tenerife	Tenerife	Laurel forest	28.560191	-16.169190	752
Dysdera iguanensis Wunderlich, 1987	Inagua	Mogán	Gran Canaria	Pine forest	27.938683	-15.708818	1161
Dysdera iguanensis Wunderlich, 1987	Monte Aguirre	Santa Cruz de Tenerife	Tenerife	Laurel forest	28.529535	-16.268309	692
Dysdera iguanensis Wunderlich, 1987	Palo Blanco	Los Realejos	Tenerife	Laurel forest	28.357941	-16.586559	869
Dysdera iguanensis Wunderlich, 1987	Roque de los Pasos. Anaga	Santa Cruz de Tenerife	Tenerife	Laurel forest	28.542914	-16.222667	929
Dysdera iguanensis Wunderlich, 1987	Vueltas de Taganana	Santa Cruz de Tenerife	Tenerife	Laurel forest	28.542082	-16.228833	833
Dysdera insulana Simon, 1883	Aguagarcía	Tacoronte	Tenerife	Pine forest	28.455283	-16.404024	928
Dysdera insulana Simon, 1883	Barranco de los Cochinos. Monte del Agua	Los Silos	Tenerife	Laurel forest	28.324550	-16.819440	950
Dysdera insulana Simon, 1883	Barranco del Río	Arico	Tenerife	Pine forest	28.170842	-16.556740	905
Dysdera insulana Simon, 1883	Cabezo del Tejo	Santa Cruz de Tenerife	Tenerife	Laurel forest	28.565090	-16.165982	80
Dysdera insulana Simon, 1883	Cruz del Carmen (path to the restaurant)	La Laguna	Tenerife	Laurel forest	28.531925	-16.279999	945
Dysdera insulana Simon, 1883	El Bailadero. Anaga	Santa Cruz de Tenerife	Tenerife	Laurel forest	28.550411	-16.203986	665
Dysdera insulana Simon, 1883	Forest road El Batán - Cruz del Carmen	La Laguna	Tenerife	Laurel forest	28.535320	-16.296800	880
Dysdera insulana Simon, 1883	Forest road Las Hiedras - Las Carboneras	La Laguna	Tenerife	Laurel forest	28.535600	-16.298810	950
Dysdera insulana Simon, 1883	Hoya de Ijuana. Anaga	Santa Cruz de Tenerife	Tenerife	Laurel forest	28.560191	-16.169190	752
Dysdera insulana Simon, 1883	Inagua	Mogán	Gran Canaria	Pine forest	27.938683	-15.708818	1161
Dysdera insulana Simon, 1883	La Esperanza	El Rosario	Tenerife	Pine forest	28.427362	-16.381073	1128
Dysdera insulana Simon, 1883	La Montañeta	Garachico	Tenerife	Pine forest	28.332726	-16.757315	1037
Dysdera insulana Simon, 1883	Monte Aguirre	Santa Cruz de Tenerife	Tenerife	Laurel forest	28.529535	-16.268309	692
Dysdera insulana Simon, 1883	Monte de Las Mercedes	La Laguna	Tenerife	Laurel forest	28.525678	-16.287059	776
Dysdera insulana Simon, 1883	Zapata	La Laguna	Tenerife	Laurel forest	28.535499	-16.296200	889
Dysdera labradaensis Wunderlich, 1994	Cueva de Felipe Reventón	Icod de los Vinos	Tenerife	Lava tube	28.350180	-16.704638	612
Dysdera labradaensis Wunderlich, 1994	Cueva del Viento. Sobrado	Icod de Los Vinos	Tenerife	Lava tube	28.345283	-16.698562	730
Dysdera labradaensis Wunderlich, 1994	Cueva Labrada	El Sauzal	Tenerife	Lava tube	28.437553	-16.413396	1698
Dysdera lancerotensis Simon, 1907	Atalaya de Femés. Los Ajaches	Yaiza	Lanzarote	Lowland xerophytic shrub	28.919520	-13.763700	380
Dysdera lancerotensis Simon, 1907	Barranco del Ciervo. Cumbres de Jandía	Pájara	Fuerteventura	Thermo-sclerophyllous woodlands	28.093768	-14.363823	686

Dysdera lancerotensis Simon, 1907	Cabecera Barranco Elvira Sánchez	Haría	Lanzarote	Thermo-sclerophyllous woodlands	29.130724	-13.516903	566
Dysdera lancerotensis Simon, 1907	Caldera. Montaña Clara	Montaña Clara	Lanzarote	Lowland xerophytic shrub	29.298860	-13.535300	241
Dysdera lancerotensis Simon, 1907	Caleta del Sebo	La Graciosa	Lanzarote	Sand dunes	29.232511	-13.505077	14
Dysdera lancerotensis Simon, 1907	Costa Calma	Pájara	Fuerteventura	Lowland xerophytic shrub	28.151492	-14.238377	64
Dysdera lancerotensis Simon, 1907	Degollada Barranco Mosquitos	Jandía	Fuerteventura	Thermo-sclerophyllous woodlands	28.087906	-14.409458	465
Dysdera lancerotensis Simon, 1907	E from Punta Ballena N from Cotillo	La Oliva	Fuerteventura	Lowland xerophytic shrub	28.715238	-14.014530	3
Dysdera lancerotensis Simon, 1907	El Cotillo. Los Lagos	La Oliva	Fuerteventura	Lowland xerophytic shrub	28.690842	-14.010146	5
Dysdera lancerotensis Simon, 1907	Famara	Haría	Lanzarote	Thermo-sclerophyllous woodlands	29.184360	-13.501310	347
Dysdera lancerotensis Simon, 1907	Femés	Femés	Lanzarote	Lowland xerophytic shrub	28.922303	-13.765028	297
Dysdera lancerotensis Simon, 1907	Fuente Ovejas. Guinate	Haría	Lanzarote	Thermo-sclerophyllous woodlands	29.184361	-13.501313	348
Dysdera lancerotensis Simon, 1907	Jable Barranco Conejos	La Graciosa	Lanzarote	Lowland xerophytic shrub (Dunes)	29.256073	-13.488980	34
Dysdera lancerotensis Simon, 1907	La Caldera (inner bottom)	Alegranza	Lanzarote	Lowland xerophytic shrub	29.399594	-13.526578	242
Dysdera lancerotensis Simon, 1907	Los Matorrales, Caleta de Famara	Teguise	Lanzarote	Lowland xerophytic shrub	29.110621	-13.551666	25
Dysdera lancerotensis Simon, 1907	Malpaís de Bayuyo	La Oliva	Fuerteventura	Lowland xerophytic shrub	28.653331	-13.902783	160
Dysdera lancerotensis Simon, 1907	Meseta de Concheta. La Caldera	Alegranza	Lanzarote	Lowland xerophytic shrub	29.399590	-13.526570	242
Dysdera lancerotensis Simon, 1907	Mirador de Morro Veloso	Betancuria	Fuerteventura	Lowland xerophytic shrub	28.441191	-14.056776	590
Dysdera lancerotensis Simon, 1907	Mirador del Río	Haría	Lanzarote	Lowland xerophytic shrub	29.211204	-13.484156	439
Dysdera lancerotensis Simon, 1907	Montaña Blanca	Tías	Lanzarote	Lowland xerophytic shrub	28.980257	-13.638351	513
Dysdera lancerotensis Simon, 1907	Montaña Clara Caldera (ridge)	Montaña Clara	Lanzarote	Lowland xerophytic shrub	29.300601	-13.538081	189
Dysdera lancerotensis Simon, 1907	Montaña del Mojón	La Graciosa	Lanzarote	Lowland xerophytic shrub	29.242060	-13.516290	135
Dysdera lancerotensis Simon, 1907	Montaña Lobos	Alegranza	Lanzarote	Lowland xerophytic shrub	29.393920	-13.501940	74
Dysdera lancerotensis Simon, 1907	Montaña Tinache	Tinajo	Lanzarote	Lowland xerophytic shrub	29.051850	-13.669850	3214
Dysdera lancerotensis Simon, 1907	Morro del Cavadero	Pájara	Fuerteventura	Thermo-sclerophyllous woodlands	28.093768	-14.363823	686
Dysdera lancerotensis Simon, 1907	Peña Laja	La Graciosa	Lanzarote	Sand dunes	29.231029	-13.508988	22
Dysdera lancerotensis Simon, 1907	Pico de la Zarza	Jandía	Fuerteventura	Thermo-sclerophyllous woodlands	28.097596	-14.362101	666
Dysdera lancerotensis Simon, 1907	Pico del Fraile	Jandía	Fuerteventura	Thermo-sclerophyllous woodlands	28.090043	-14.394320	657
Dysdera lancerotensis Simon, 1907	Playa de Teneza	Tinajo	Lanzarote	Lowland xerophytic shrub (Pebbled beaches)	29.079730	-13.715702	1
Dysdera lancerotensis Simon, 1907	Playa Lambra	La Graciosa	La Graciosa	Lowland xerophytic shrub (Pebbled beaches)	29.277603	-13.490806	8
Dysdera lancerotensis Simon, 1907	Valle de Fenauco. Los Lomos	Yaiza	Lanzarote	Lowland xerophytic shrub	28.932870	-13.773660	282
Dysdera lancerotensis Simon, 1907	Valle de Fuentedulce. Órzola	Haría	Lanzarote	Thermo-sclerophyllous woodlands	29.202288	-13.461592	115
Dysdera lancerotensis Simon, 1907	Valle de Haría	Haría	Lanzarote	Thermo-sclerophyllous woodlands	29.152247	-13.502376	281
Dysdera lancerotensis Simon, 1907	Zonzamas	San Bartolomé	Lanzarote	Lowland xerophytic shrub	29.012830	-13.569950	185

Dysdera levipes Wunderlich, 1987	Barranco de Nieto	Santa Cruz de Tenerife	Tenerife	Laurel forest	28.534070	-16.316255	793
Dysdera levipes Wunderlich, 1987	Barranco del Río	Arico	Tenerife	Pine forest	28.170842	-16.556740	905
Dysdera levipes Wunderlich, 1987	Cabezo del Tejo	Santa Cruz de Tenerife	Tenerife	Laurel forest	28.562022	-16.171397	805
Dysdera levipes Wunderlich, 1987	Cabezo del Tejo	Santa Cruz de Tenerife	Tenerife	Laurel forest	28.565090	-16.165982	805
Dysdera levipes Wunderlich, 1987	Cruz del Carmen (path to the restaurant)	La Laguna	Tenerife	Laurel forest	28.531925	-16.279999	945
Dysdera levipes Wunderlich, 1987	El Montillo	La Matanza	Tenerife	Lowland xerophytic shrub	28.442525	-16.454611	500
Dysdera levipes Wunderlich, 1987	Enchereda (towards degollada)	San Sebastián	La Gomera	Laurel forest	28.136650	-17.179063	915
Dysdera levipes Wunderlich, 1987	Forest path between Barranco Higuera and Barranco San Juan	Vallehermoso	La Gomera	Laurel forest	28.193320	-17.292940	695
Dysdera levipes Wunderlich, 1987	Forest road El Batán - Cruz del Carmen	La Laguna	Tenerife	Laurel forest	28.535320	-16.296800	880
Dysdera levipes Wunderlich, 1987	Forest road Las Hiedras- La Carboneras	La Laguna	Tenerife	Laurel forest	28.535600	-16.298810	950
Dysdera levipes Wunderlich, 1987	Ijuana forest road	Santa Cruz de Tenerife	Tenerife	Laurel forest	28.560191	-16.169190	752
Dysdera levipes Wunderlich, 1987	Inagua	Mogán	Gran Canaria	Pine forest	27.938683	-15.708818	1161
Dysdera levipes Wunderlich, 1987	Monte Aguirre	Santa Cruz de Tenerife	Tenerife	Laurel forest	28.529535	-16.268309	692
Dysdera levipes Wunderlich, 1987	Pajarito	Hermigua	La Gomera	Laurel forest	28.108856	-17.241604	1339
Dysdera levipes Wunderlich, 1987	Palo Blanco	Los Realejos	Tenerife	Laurel forest	28.357941	-16.586559	869
Dysdera levipes Wunderlich, 1987	Pinar de Ifonche	Adeje	Tenerife	Pine forest	28.141800	-16.691400	1037
Dysdera levipes Wunderlich, 1987	Pinoleris	La Orotava	Tenerife	Pine forest	28.400377	-16.496257	761
Dysdera levipes Wunderlich, 1987	Teno Alto	Buenavista	Tenerife	Laurel forest	28.335996	-16.865760	861
Dysdera levipes Wunderlich, 1987	Teselinde. Ermita de Santa Clara	Vallehermoso	La Gomera	Laurel forest	28.196300	-17.287540	727
Dysdera levipes Wunderlich, 1987	Zapata	La Laguna	Tenerife	Laurel forest	28.535499	-16.296200	889
Dysdera liostethus Simon, 1907	Andén Verde	Artenara	Gran Canaria	Lowland xerophytic shrub	28.035189	-15.746346	677
Dysdera liostethus Simon, 1907	Barranco de Tirajana	Santa Lucía de Tirajana	Gran Canaria	Lowland xerophytic shrub	27.847879	-15.483428	159
Dysdera liostethus Simon, 1907	Barranco El Draguillo	Ingenio	Gran Canaria	Lowland xerophytic shrub	27.941568	-15.429992	200
Dysdera liostethus Simon, 1907	Barranco Tasartico	San Nicolás de Tolentino	Gran Canaria	Midland xerophytic shrub	27.930859	-15.791131	316
Dysdera liostethus Simon, 1907	Brezal del Palmital	Santa María de Guía	Gran Canaria	Laurel forest	28.111450	-15.601970	495
Dysdera liostethus Simon, 1907	Caideros	Gáldar	Gran Canaria	Thermo-sclerophyllous woodlands	28.075760	-15.649420	840
Dysdera liostethus Simon, 1907	Caldera de Bandama	Las Palmas de GC	Gran Canaria	Thermo-sclerophyllous woodlands	28.032912	-15.460607	459
Dysdera liostethus Simon, 1907	Caldera de los Marteles	Telde	Gran Canaria	Thermo-sclerophyllous woodlands	27.952243	-15.527475	1536
Dysdera liostethus Simon, 1907	Cortijo San Gregorio. Tamaraceite	Las Palmas de GC	Gran Canaria	Thermo-sclerophyllous woodlands	28.085788	-15.491280	320
Dysdera liostethus Simon, 1907	Degollada Cueva de la Negra. Inagua	Tejeda	Gran Canaria	Pine forest	27.932851	-15.646300	964

Dysdera liostethus Simon, 1907	Degollada de Las Brujas	Mogán	Gran Canaria	Pine forest	27.940540	-15.731890	1220
Dysdera liostethus Simon, 1907	El Sao	Agaete	Gran Canaria	Pine forest	28.068764	-15.656990	480
Dysdera liostethus Simon, 1907	Forest road to Tirma	Artenara	Gran Canaria	Lowland xerophytic shrub	28.032133	-15.755400	610
Dysdera liostethus Simon, 1907	Inagua	Mogán	Gran Canaria	Pine forest	27.938683	-15.708818	1161
Dysdera liostethus Simon, 1907	Llanos de la Pez	Tejeda	Gran Canaria	Pine forest	27.964312	-15.585547	1662
Dysdera liostethus Simon, 1907	Los Majaletes	Ingenio	Gran Canaria	Thermo-sclerophyllous woodlands	27.940161	-15.499200	900
Dysdera liostethus Simon, 1907	Mina de los Llanetes	Valsequillo	Gran Canaria	Thermo-sclerophyllous woodlands	27.988402	-15.478981	396
Dysdera liostethus Simon, 1907	Pico Viento	Gáldar	Gran Canaria	Lowland xerophytic shrub	28.099788	-15.651685	805
Dysdera liostethus Simon, 1907	Playa del Risco	Agaete	Gran Canaria	Lowland xerophytic shrub (Pebbled beaches)	28.052779	-15.739769	5
Dysdera liostethus Simon, 1907	Tirajana (Pine forest)	San Bartolome de Tirajana	Gran Canaria	Pine forest	27.908870	-15.570200	930
Dysdera longa Wunderlich, 1992	Barranco del Ciervo. Cumbres de Jandía	Pájara	Fuerteventura	Thermo-sclerophyllous woodlands	28.093768	-14.363823	686
Dysdera longa Wunderlich, 1992	Cumbre de Jandía	Pájara	Fuerteventura	Thermo-sclerophyllous woodlands	28.094636	-14.440265	429
Dysdera longa Wunderlich, 1992	Morro del Cavadero	Pájara	Fuerteventura	Thermo-sclerophyllous woodlands	28.093768	-14.363823	686
Dysdera longa Wunderlich, 1992	Morro del Jorao	Jandía	Fuerteventura	Thermo-sclerophyllous woodlands	28.117460	-14.334534	578
Dysdera longa Wunderlich, 1992	Pico de la Zarza	Jandía	Fuerteventura	Thermo-sclerophyllous woodlands	28.097596	-14.362101	666
Dysdera longa Wunderlich, 1992	Pico del Fraile	Jandía	Fuerteventura	Thermo-sclerophyllous woodlands	28.090043	-14.394320	657
Dysdera macra Simon, 1883	Barranco de Badajoz	Güímar	Tenerife	Laurel forest	28.303927	-16.440052	595
Dysdera macra Simon, 1883	Barranco de San Andrés	Santa Cruz de Tenerife	Tenerife	Lowland xerophytic shrub	28.519926	-16.189101	455
Dysdera macra Simon, 1883	Barranco del Agua	Güímar	Tenerife	Laurel forest	28.307838	-16.448156	800
Dysdera macra Simon, 1883	Barranco del Pino	Santa Úrsula	Tenerife	Pine forest	28.384151	-16.475028	1375
Dysdera macra Simon, 1883	Barranco del Río	Arico	Tenerife	Pine forest	28.170842	-16.556740	905
Dysdera macra Simon, 1883	Barranco Samarines	Candelaria	Tenerife	Tabaibal-Cardonal	28.345548	-16.371103	27
Dysdera macra Simon, 1883	Base Zig-Zag. Teide National Park	La Orotava	Tenerife	Dry subalpine scrub	28.271970	-16.614630	2686
Dysdera macra Simon, 1883	Caldera de Pedro Gil	Arico	Tenerife	Pine forest	28.248283	-16.529917	1790
Dysdera macra Simon, 1883	Candelaria (close to pig farm)	Candelaria	Tenerife	Lowland xerophytic shrub	28.343010	-16.383854	144
Dysdera macra Simon, 1883	Cañada de Diego Hernández. Teide National Park	La Orotava	Tenerife	Dry subalpine scrub	28.277097	-16.551003	2095
Dysdera macra Simon, 1883	Cañadas del Teide	La Orotava	Tenerife	Dry subalpine scrub	28.288195	-16.573390	2184
Dysdera macra Simon, 1883	Close to Ucanca hut. Teide National Park	La Orotava	Tenerife	Dry subalpine scrub	28.211143	-16.627367	2200
Dysdera macra Simon, 1883	Cueva de Cosme	Arafo-Güímar	Tenerife	Lava tube	28.340410	-16.471530	1616
Dysdera macra Simon, 1883	Cumbre Bolico	Santiago del Teide	Tenerife	Laurel forest	28.314186	-16.826940	1191
Dysdera macra Simon, 1883	Cumbres de Arico. Trail from Izaña	Arico	Tenerife	Pine forest	28.249217	-16.528748	1950

Dysdera macra Simon, 1883	El Aderno, over Buenavista	Buenavista del Norte	Tenerife	Thermo-sclerophyllous woodlands	28.358258	-16.864456	200
Dysdera macra Simon, 1883	El Diablillo	La Victoria de Acentejo	Tenerife	Pine forest	28.404818	-16.423071	1659
Dysdera macra Simon, 1883	El Escobonal	Güímar	Tenerife	Lowland xerophytic shrub	28.267749	-16.425706	562
Dysdera macra Simon, 1883	El Guanche. Trail to Chimoche. Aguamansa	La Orotava	Tenerife	Pine forest	28.347186	-16.514020	1425
Dysdera macra Simon, 1883	El Lagar. Llano del Hospital forest road	La Guancha	Tenerife	Pine forest	28.335544	-16.657566	1065
Dysdera macra Simon, 1883	El Palmar	Arona	Tenerife	Urban area	28.022870	-16.694530	45
Dysdera macra Simon, 1883	El Pinalito	Vilaflor	Tenerife	Pine forest	28.183345	-16.636379	1866
Dysdera macra Simon, 1883	El Portillo	La Orotava	Tenerife	Dry subalpine scrub	28.296899	-16.565151	2115
Dysdera macra Simon, 1883	Forest road to Benijos	Los Realejos	Tenerife	Pine forest	28.338981	-16.547893	1292
Dysdera macra Simon, 1883	Forest road to Los Órganos (beyond los Sanguinos Forest)	Santa Úrsula	Tenerife	Pine forest	28.362888	-16.483766	1295
Dysdera macra Simon, 1883	Forest road to Madre del Agua- Barranco del Río	Vilaflor	Tenerife	Pine forest	28.177310	-16.601628	1710
Dysdera macra Simon, 1883	Guergue	Buenavista	Tenerife	Midland xerophytic shrub	28.292050	-16.846042	910
Dysdera macra Simon, 1883	Izaña	La Orotava	Tenerife	Dry subalpine scrub	28.306892	-16.514530	2237
Dysdera macra Simon, 1883	La Caldera. Aguamansa	La Orotava	Tenerife	Pine forest	28.356576	-16.499748	1199
Dysdera macra Simon, 1883	La Fortaleza. Teide National Park	La Orotava	Tenerife	Subalpine shrubs	28.316723	-16.591240	2070
Dysdera macra Simon, 1883	Las Arenas	Santiago del Teide	Tenerife	Thermo-sclerophyllous woodlands	28.299588	-16.795435	1200
Dysdera macra Simon, 1883	Las Cañadas del Teide	La Orotava	Tenerife	Dry subalpine scrub	28.288195	-16.573390	2184
Dysdera macra Simon, 1883	Las Chafiras crossroad (highway)	San Miguel	Tenerife	Lowland xerophytic shrub	28.053842	-16.598014	140
Dysdera macra Simon, 1883	Las Lagunetas	El Rosario	Tenerife	Pine forest	28.418535	-16.410090	1400
Dysdera macra Simon, 1883	Las Lajas picnic place	Vilaflor	Tenerife	Pine forest	28.190338	-16.669163	2050
Dysdera macra Simon, 1883	Las Raíces	El Rosario	Tenerife	Pine forest	28.435580	-16.373500	1200
Dysdera macra Simon, 1883	Los Lomitos. Monte de Las Mesas	Santa Cruz de Tenerife	Tenerife	Semi-arid tropical shrubs	28.481365	-16.263627	390
Dysdera macra Simon, 1883	Madre del Agua	Granadilla	Tenerife	Pine forest	28.169368	-16.630688	1700
Dysdera macra Simon, 1883	Malpaís de Rasca	Arona	Tenerife	Lowland xerophytic shrub	28.018203	-16.690674	52
Dysdera macra Simon, 1883	Mirador sobre La Rosa de Piedra	La Orotava	Tenerife	Pine forest	28.339491	-16.524285	1514
Dysdera macra Simon, 1883	Mirador Vista a La Palma	La Orotava	Tenerife	Pine forest	28.347912	-16.516101	1383
Dysdera macra Simon, 1883	Montaña Chusqueros. Siete Cañadas	La Orotava	Tenerife	Subalpine shrubs	28.292240	-16.559003	2099
Dysdera macra Simon, 1883	Montaña Roja. El Médano	Granadilla de Abona	Tenerife	Lowland xerophytic shrub	28.030936	-16.545984	49
Dysdera macra Simon, 1883	Montaña Viña Vieja	San Miguel	Tenerife	Lowland xerophytic shrub	28.102705	-16.608081	656
Dysdera macra Simon, 1883	Monte de Santa Úrsula. La Corujera	Santa Úrsula	Tenerife	Laurel forest	28.403179	-16.486186	880
Dysdera macra Simon, 1883	Morada del Viento (Siete Fuentes road). Las Lagunetas	El Rosario	Tenerife	Pine forest	28.411180	-16.421127	1540

Dysdera macra Simon, 1883	Orticosa. Choza de la Loca	Arafo	Tenerife	Pine forest	28.384498	-16.447410	1450
Dysdera macra Simon, 1883	Pinar de Chío	Guía de Isora	Tenerife	Pine forest	28.241833	-16.763415	1184
Dysdera macra Simon, 1883	Pinar de Ifonche	Adeje	Tenerife	Pine forest	28.141800	-16.691400	1037
Dysdera macra Simon, 1883	Pine forest Roque del Peral. Teide National Park	Los Realejos	Tenerife	Pine forest	28.308159	-16.576300	2000
Dysdera macra Simon, 1883	Polígono Industrial Arafo-Güímar	Güímar	Tenerife	Lowland xerophytic shrub	28.343305	-16.374289	70
Dysdera macra Simon, 1883	Retamar (3050 m). Teide National Park	La Orotava	Tenerife	Dry subalpine scrub	28.273448	-16.625179	3054
Dysdera macra Simon, 1883	Retamar Denso. Teide National Park	La Orotava	Tenerife	Dry subalpine scrub	28.299317	-16.601028	2121
Dysdera macra Simon, 1883	Rincón de Atanasio (Old road)	Candelaria	Tenerife	Lowland xerophytic shrub	28.356447	-16.380447	112
Dysdera macra Simon, 1883	Roque del Conde	Adeje	Tenerife	Xerophytic scrubs	28.093144	-16.698890	525
Dysdera macra Simon, 1883	Rosa de Piedra	La Orotava	Tenerife	Pine forest	28.340555	-16.524742	1484
Dysdera macra Simon, 1883	Siete Cañadas. Teide National Park	La Orotava	Tenerife	Dry subalpine scrub	28.292240	-16.559003	2099
Dysdera macra Simon, 1883	Tabaiba	El Rosario	Tenerife	Xerophytic scrubs	28.407689	-16.328955	230
Dysdera macra Simon, 1883	Teno Alto	Buenavista	Tenerife	Laurel forest	28.335996	-16.865760	861
Dysdera macra Simon, 1883	Torre del Gaitero	Arafo	Tenerife	Pine forest	28.394697	-16.431984	1735
Dysdera macra Simon, 1883	Valle de San Lorenzo (crossroad)	Arona	Tenerife	Lowland xerophytic shrub	28.054350	-16.677984	109
Dysdera macra Simon, 1883	Valle Jiménez	Santa Cruz de Tenerife	Tenerife	Thermo-sclerophyllous woodlands	28.498880	-16.273290	481
Dysdera macra Simon, 1883	Volcán Chuchurumbache	Granadilla	Tenerife	Lowland xerophytic shrub	28.097330	-16.583230	515
Dysdera macra Simon, 1883	Barranco de los Cochinos. Monte del Agua	Los Silos	Tenerife	Laurel forest	28.324550	-16.819440	950
Dysdera macra Simon, 1883	Montaña Blanca. Teide National Park	La Orotava	Tenerife	Dry subalpine scrub	28.270218	-16.597251	513
Dysdera mahan Macías-Hernández & Arnedo, 2010	Caleta de Arriba	La Graciosa	Lanzarote	Sand dunes	29.244485	-13.492957	7
Dysdera mahan Macías-Hernández & Arnedo, 2010	Caleta de Famara	Haría	Lanzarote	Sand dunes	29.203408	-13.424308	10
Dysdera mahan Macías-Hernández & Arnedo, 2010	Malpaís de la Corona. Tide ponds	Haría	Lanzarote	Lowland xerophytic shrub (Dunes)	29.194456	-13.427880	30
Dysdera mahan Macías-Hernández & Arnedo, 2010	Playa Caleta del Congrio. Papagayo	Yaiza	Lanzarote	Lowland xerophytic shrub (Pebbled beaches)	28.838823	-13.787028	1
Dysdera mahan Macías-Hernández & Arnedo, 2010	Playa Caleta del Mariscadero. Timanfaya	Tinajo	Lanzarote	Lowland xerophytic shrub (Pebbled beaches)	29.064238	-13.765097	8.5
Dysdera mahan Macías-Hernández & Arnedo, 2010	Playa Catalina Cabrera. Famara	Haría	Lanzarote	Lowland xerophytic shrub (Pebbled beaches)	29.194448	-13.505116	7
Dysdera mahan Macías-Hernández & Arnedo, 2010	Playa de El Trillo	Alegranza	Lanzarote	Lowland xerophytic shrub (Pebbled beaches)	29.404184	-13.490834	2
Dysdera mahan Macías-Hernández & Arnedo, 2010	Playa de Esquinzo	La Oliva	Fuerteventura	Lowland xerophytic shrub (Pebbled beaches)	28.636453	-14.025551	35
Dysdera mahan Macías-Hernández & Arnedo, 2010	Playa de Teneza	Tinajo	Lanzarote	Lowland xerophytic shrub (Pebbled beaches)	29.079730	-13.715702	1
Dysdera mahan Macías-Hernández & Arnedo, 2010	Playa la Madera. Timanfaya	Tinajo	Lanzarote	Lowland xerophytic shrub (Pebbled beaches)	29.062068	-13.774604	4

Dysdera mahan Macías-Hernández & Arnedo, 2010	Playa Las Salinas. Lobos	Lobos	Fuerteventura	Lowland xerophytic shrub (Pebbled beaches)	28.743173	-13.829946	3
Dysdera mahan Macías-Hernández & Arnedo, 2010	Playa Las Salinas. Puerto Calero	Yaiza	Lanzarote	Lowland xerophytic shrub (Pebbled beaches)	28.918115	-13.698886	6
Dysdera mahan Macías-Hernández & Arnedo, 2010	Playa Majanicho	Corralejo	Fuerteventura	Lowland xerophytic shrub (Pebbled beaches)	28.744863	-13.873707	3
Dysdera mahan Macías-Hernández & Arnedo, 2010	Punta Pasitos. Mala	Haría	Lanzarote	Lowland xerophytic shrub (Pebbled beaches)	29.104336	-13.455999	7
Dysdera minutissima Wunderlich, 1992	Aguamansa	La Orotava	Tenerife	Pine forest	28.356576	-16.499748	1200
Dysdera minutissima Wunderlich, 1992	Barranco del Pino	Santa Úrsula	Tenerife	Pine forest	28.384151	-16.475028	1375
Dysdera minutissima Wunderlich, 1992	La Caldera, Aguamansa, Lomo Colorado forest road	La Orotava	Tenerife	Pine forest	28.359658	-16.501015	1141
Dysdera montanetensis Wunderlich, 1992	Aguamansa	La Orotava	Tenerife	Pine forest	28.356576	-16.499748	1200
Dysdera montanetensis Wunderlich, 1992	Barranco de los Cochinos. Monte del Agua	Los Silos	Tenerife	Laurel forest	28.324550	-16.819440	950
Dysdera montanetensis Wunderlich, 1992	Cueva Labrada	El Sauzal	Tenerife	Lava tube	28.437553	-16.413396	1698
Dysdera montanetensis Wunderlich, 1992	La Montañeta	Garachico	Tenerife	Pine forest	28.332726	-16.757315	1037
Dysdera montanetensis Wunderlich, 1992	Las Raíces	El Rosario	Tenerife	Pine forest	28.435580	-16.373500	1200
Dysdera nesiotes Simon, 1907	Atalaya de Femés. Los Ajaches	Yaiza	Lanzarote	Lowland xerophytic shrub	28.919520	-13.763700	380
Dysdera nesiotes Simon, 1907	Barranco Hondo del Valle	Haría	Lanzarote	Thermo-sclerophyllous woodlands	29.140800	-13.483200	125
Dysdera nesiotes Simon, 1907	Cabecera Barranco Elvira Sánchez	Haría	Lanzarote	Thermo-sclerophyllous woodlands	29.130724	-13.516903	566
Dysdera nesiotes Simon, 1907	Caldera de Montaña Lobos	Alegranza	Lanzarote	Lowland xerophytic shrub	29.393920	-13.501940	74
Dysdera nesiotes Simon, 1907	Caldera. Montaña Clara	Montaña Clara	Lanzarote	Lowland xerophytic shrub	29.298860	-13.535300	241
Dysdera nesiotes Simon, 1907	Famara Mountains	Haría	Lanzarote	Thermo-sclerophyllous woodlands	29.211204	-13.484156	440
Dysdera nesiotes Simon, 1907	Femés	Femés	Lanzarote	Lowland xerophytic shrub	28.922303	-13.765028	297
Dysdera nesiotes Simon, 1907	Fuente Ovejas. Guinate	Haría	Lanzarote	Thermo-sclerophyllous woodlands	29.184361	-13.501313	348
Dysdera nesiotes Simon, 1907	Máguez	Haría	Lanzarote	Thermo-sclerophyllous woodlands	29.154410	-13.519440	268
Dysdera nesiotes Simon, 1907	Meseta de Concheta. La Caldera	Alegranza	Lanzarote	Lowland xerophytic shrub	29.399590	-13.526570	242
Dysdera nesiotes Simon, 1907	Mirador del Río	Haría	Lanzarote	Lowland xerophytic shrub	29.211204	-13.484156	439
Dysdera nesiotes Simon, 1907	Montaña Blanca	Tías	Lanzarote	Lowland xerophytic shrub	28.980257	-13.638351	513
Dysdera nesiotes Simon, 1907	Montaña Tinache	Tinajo	Lanzarote	Lowland xerophytic shrub	29.051850	-13.669850	3214
Dysdera nesiotes Simon, 1907	Valle de Fenauco. Los Lomos	Yaiza	Lanzarote	Lowland xerophytic shrub	28.932870	-13.773660	282
Dysdera nesiotes Simon, 1907	Valle de Haría	Haría	Lanzarote	Thermo-sclerophyllous woodlands	29.152247	-13.502376	281
Dysdera nesiotes Simon, 1907	Valle de Malpaso	Haría	Lanzarote	Thermo-sclerophyllous woodlands	29.135308	-13.507113	360
Dysdera orahan Arnedo, Oromí & Ribera, 1997	Mirador de Bascos	Frontera	El Hierro	Thermo-sclerophyllous woodlands	27.754861	-18.118218	658
Dysdera orahan Arnedo, Oromí & Ribera, 1997	Puntallana	San Sebastián	La Gomera	Lowland xerophytic shrub (Dunes)	28.127989	-17.106447	1037
Dysdera orahan Arnedo, Oromí & Ribera, 1997	Ventejís. Tiñor	Valverde	El Hierro	Pine forest	27.785991	-17.937508	1008

Dysdera paucispinosa Wunderlich, 1992	Degollada Cueva de la Negra. Inagua	Tejeda	Gran Canaria	Pine forest	27.932851	-15.646300	964
Dysdera paucispinosa Wunderlich, 1992	Degollada de Becerra	Tejeda	Gran Canaria	Pine forest	27.989730	-15.592920	1565
Dysdera paucispinosa Wunderlich, 1992	Degollada de Las Brujas	Mogán	Gran Canaria	Pine forest	27.940540	-15.731890	1220
Dysdera paucispinosa Wunderlich, 1992	Inagua	Mogán	Gran Canaria	Pine forest	27.938683	-15.708818	1161
Dysdera paucispinosa Wunderlich, 1992	Los Berrazales	Agaete	Gran Canaria	Pine forest	28.068779	-15.658864	435
Dysdera paucispinosa Wunderlich, 1992	Pinar de Pajonales	Tejeda	Gran Canaria	Pine forest	27.943295	-15.678000	1280
Dysdera paucispinosa Wunderlich, 1992	Tamadaba	Agaete	Gran Canaria	Pine forest	28.031840	-15.677020	1134
Dysdera ramblae Arnedo, Oromí & Ribera, 1997	Apartacaminos	Vallehermoso	La Gomera	Laurel forest	28.155349	-17.300600	945
Dysdera ramblae Arnedo, Oromí & Ribera, 1997	Barranco de Juel	San Sebastián	La Gomera	Laurel forest	28.151547	-17.162962	640
Dysdera ramblae Arnedo, Oromí & Ribera, 1997	Camino forestal Meriga - El Cedro	Agulo	La Gomera	Laurel forest	28.168996	-17.222357	840
Dysdera ramblae Arnedo, Oromí & Ribera, 1997	El Cedro	Hermigua	La Gomera	Laurel forest	28.137044	-17.222569	890
Dysdera ramblae Arnedo, Oromí & Ribera, 1997	Enchereda (towards degollada)	San Sebastián	La Gomera	Laurel forest	28.136650	-17.179063	915
Dysdera ramblae Arnedo, Oromí & Ribera, 1997	La Asomadita (Chipude forest road)	Vallehermoso	La Gomera	Laurel forest	28.111600	-17.264800	1255
Dysdera ramblae Arnedo, Oromí & Ribera, 1997	La Campana	Hermigua	La Gomera	Laurel forest	28.158055	-17.160623	730
Dysdera ramblae Arnedo, Oromí & Ribera, 1997	Laguna Grande	Agulo	La Gomera	Laurel forest	28.125420	-17.257171	1255
Dysdera ramblae Arnedo, Oromí & Ribera, 1997	Las Creces	P.N. Garajonay	La Gomera	Laurel forest	28.138714	-17.287851	1050
Dysdera ramblae Arnedo, Oromí & Ribera, 1997	Los Noruegos	Hermigua	La Gomera	Laurel forest	28.106905	-17.233323	1360
Dysdera ramblae Arnedo, Oromí & Ribera, 1997	Los Pajaritos	Hermigua	La Gomera	Laurel forest	28.108856	-17.241604	1355
Dysdera ramblae Arnedo, Oromí & Ribera, 1997	Monte de Juan Tomé. La Laja	San Sebastián	La Gomera	Laurel forest	28.123022	-17.210137	1035
Dysdera ramblae Arnedo, Oromí & Ribera, 1997	Monte del Cedro	Hermigua	La Gomera	Laurel forest	28.119283	-17.237290	1260
Dysdera ramblae Arnedo, Oromí & Ribera, 1997	Mora de Gaspar	Vallehermoso	La Gomera	Laurel forest	28.137564	-17.254264	1085
Dysdera ramblae Arnedo, Oromí & Ribera, 1997	Road Juego de Bolas - Laguna Grande	Agulo	La Gomera	Laurel forest	28.129925	-17.254707	1184
Dysdera ramblae Arnedo, Oromí & Ribera, 1997	Taguluche	Vallehermoso	La Gomera	Xerophytic scrubs	28.155201	-17.311055	580
Dysdera ramblae Arnedo, Oromí & Ribera, 1997	Teselinde. Ermita de Santa Clara	Vallehermoso	La Gomera	Laurel forest	28.196300	-17.287540	727
Dysdera ratonensis Wunderlich, 1992	Cueva Callejones	Mazo	La Palma	Lava tube	28.602790	-17.763046	194
Dysdera ratonensis Wunderlich, 1992	Cueva de La Fajanita	Garafía	La Palma	Lava tube	28.839789	-17.893348	96
Dysdera ratonensis Wunderlich, 1992	Cueva de los Arreboles	Fuencaliente	La Palma	Lava tube	28.493785	-17.829030	430
Dysdera ratonensis Wunderlich, 1992	Cueva de Los Caños	Mazo	La Palma	Lava tube	28.579496	-17.799226	943
Dysdera ratonensis Wunderlich, 1992	Cueva de los Murciélagos II	San Andrés y Sauces	La Palma	Lava tube	28.774036	-17.823787	1177
Dysdera ratonensis Wunderlich, 1992	Cueva de Los Palmeros	Fuencaliente	La Palma	Lava tube	28.507538	-17.856808	682
Dysdera ratonensis Wunderlich, 1992	Cueva de Tacande	Mazo	La Palma	Lava tube	28.640046	-17.880740	669
Dysdera ratonensis Wunderlich, 1992	Cueva del Ratón	Fuencaliente	La Palma	Lava tube	28.462557	-17.846746	165

Dysdera ratonensis Wunderlich, 1992	Cueva del Salto de Tigalate	Mazo	La Palma	Lava tube	28.534501	-17.793949	72
Dysdera ratonensis Wunderlich, 1992	Cueva El Canal	Mazo	La Palma	Lava tube	28.539763	-17.801238	365
Dysdera ratonensis Wunderlich, 1992	Cueva Honda de Gallegos	Garafía	La Palma	Lava tube	28.828932	-17.819993	449
Dysdera rugichelis Simon, 1909	Barranco de Guayadeque	Agüimes	Gran Canaria	Lowland xerophytic shrub	27.932980	-15.480070	550
Dysdera rugichelis Simon, 1909	Cumbre de Pajonales	Tejeda	Gran Canaria	Pine forest	27.943295	-15.678000	1280
Dysdera rugichelis Simon, 1909	Fontanales	Moya	Gran Canaria	Thermo-sclerophyllous woodlands	28.055530	-15.608730	1050
Dysdera rugichelis Simon, 1909	Inagua	Mogán	Gran Canaria	Pine forest	27.938683	-15.708818	1161
Dysdera rugichelis Simon, 1909	Los Majaletes	Ingenio	Gran Canaria	Thermo-sclerophyllous woodlands	27.940161	-15.499200	900
Dysdera rugichelis Simon, 1909	Pinar de Tamadaba	Agaete	Gran Canaria	Pine forest	28.031840	-15.677020	1134
Dysdera rugichelis Simon, 1909	Pinar de Tamadaba, forest house	Agaete	Gran Canaria	Pine forest	28.053618	-15.689764	1262
Dysdera sanborondon Arnedo, Oromí & Ribera, 2000	Cuchillos de Jacomar	Tuineje	Fuerteventura	Lowland xerophytic shrub	28.275507	-13.913101	51
Dysdera sanborondon Arnedo, Oromí & Ribera, 2000	Mirador de Morro Veloso	Betancuria	Fuerteventura	Lowland xerophytic shrub	28.441191	-14.056776	590
Dysdera sanborondon Arnedo, Oromí & Ribera, 2000	Montaña de la Cruz	Betancuria	Fuerteventura	Lowland xerophytic shrub	28.441619	-14.057354	592
Dysdera sanborondon Arnedo, Oromí & Ribera, 2000	Morro Tabaiba. Vallebrón	La Oliva	Fuerteventura	Lowland xerophytic shrub	28.592252	-13.943331	398
Dysdera sanborondon Arnedo, Oromí & Ribera, 2000	Tegú (road Antigua-Betancuria)	Betancuria	Fuerteventura	Lowland xerophytic shrub	28.430451	-14.046955	639
Dysdera sibyllina Arnedo, 2007	Cueva de Felipe Reventón	Icod de los Vinos	Tenerife	Lava tube	28.350180	-16.704638	612
Dysdera sibyllina Arnedo, 2007	Cueva del Viento. Breveritas	Icod de los Vinos	Tenerife	Lava tube	28.350010	-16.702820	625
Dysdera sibyllina Arnedo, 2007	Cueva del Viento. Sobrado	Icod de los Vinos	Tenerife	Lava tube	28.345283	-16.698562	730
Dysdera silvatica Schmidt, 1981	Barranco Aramaqué. Near Los Aceviños	Hermigua	La Gomera	Laurel forest	28.149151	-17.220779	855
Dysdera silvatica Schmidt, 1981	Barranco de Juel	San Sebastián	La Gomera	Laurel forest	28.151547	-17.162962	640
Dysdera silvatica Schmidt, 1981	Barranco de la Traves (PNCT)	El Paso	La Palma	Pine forest	28.717984	-17.893314	1100
Dysdera silvatica Schmidt, 1981	Barranco de Majona	San Sebastián	La Gomera	Lowland xerophytic shrub	28.151830	-17.139235	88
Dysdera silvatica Schmidt, 1981	Barranco de Matarnos. Monte del Cedro	Hermigua	La Gomera	Laurel forest	28.119131	-17.215838	1117
Dysdera silvatica Schmidt, 1981	Barranco de Paijén	San Sebastián	La Gomera	Lowland xerophytic shrub	28.088450	-17.201420	920
Dysdera silvatica Schmidt, 1981	Bosque de Arure	Valle Gran Rey	La Gomera	Laurel forest	28.133210	-17.312446	838
Dysdera silvatica Schmidt, 1981	Bosque del Cedro	Hermigua	La Gomera	Laurel forest	28.137044	-17.222569	1113
Dysdera silvatica Schmidt, 1981	Chorros de Epina	Vallehermoso	La Gomera	Laurel forest	28.167036	-17.305892	640
Dysdera silvatica Schmidt, 1981	Close to Los Aceviños	Bosque del Cedro	La Gomera	Laurel forest	28.147358	-17.219868	919
Dysdera silvatica Schmidt, 1981	Cueva de Jinama	Valverde	El Hierro	Lava tube	27.773536	-17.984089	1190
Dysdera silvatica Schmidt, 1981	Cueva de Longueras	Frontera	El Hierro	Lava tube	27.746730	-18.026256	445
Dysdera silvatica Schmidt, 1981	El Castillo	Garafía	La Palma	Pine forest	28.796360	-17.971169	501
Dysdera silvatica Schmidt, 1981	Forest path between Barranco Higuera and Barranco San Juan	Vallehermoso	La Gomera	Laurel forest	28.193320	-17.292940	695

Dysdera silvatica Schmidt, 1981	Forest road to El Derrabado	Frontera	El Hierro	Laurel forest	27.740804	-18.053080	919
Dysdera silvatica Schmidt, 1981	Forest road to El Mercader	El Pinar	El Hierro	Pine forest	27.712944	-18.022175	1075
Dysdera silvatica Schmidt, 1981	Forest road to Machín	Garafía	La Palma	Pine forest	28.789710	-17.896884	1267
Dysdera silvatica Schmidt, 1981	Fuente Mencáfite	El Golfo	El Hierro	Laurel forest	27.735497	-18.085804	930
Dysdera silvatica Schmidt, 1981	Hoya del Pino	Frontera	El Hierro	Pine forest	27.737154	-18.046305	1000
Dysdera silvatica Schmidt, 1981	Juan Adalid	Garafía	La Palma	Thermo-sclerophyllous woodlands	28.843639	-17.906350	296
Dysdera silvatica Schmidt, 1981	La Campana	Hermigua	La Gomera	Laurel forest	28.158055	-17.160623	730
Dysdera silvatica Schmidt, 1981	La Zarcita	Hermigua	La Gomera	Laurel forest	28.111342	-17.218806	1170
Dysdera silvatica Schmidt, 1981	Laguna Grande	Hermigua	La Gomera	Laurel forest	28.126085	-17.233223	1250
Dysdera silvatica Schmidt, 1981	Las Creces	P.N. Garajonay	La Gomera	Laurel forest	28.138737	-17.287852	1050
Dysdera silvatica Schmidt, 1981	Las Tajoras	P.N. Garajonay	La Gomera	Laurel forest	28.112736	-17.262511	1248
Dysdera silvatica Schmidt, 1981	Los Noruegos	Hermigua	La Gomera	Laurel forest	28.106905	-17.233323	1360
Dysdera silvatica Schmidt, 1981	Mirador de Bascos	Frontera	El Hierro	Thermo-sclerophyllous woodlands	27.754861	-18.118218	658
Dysdera silvatica Schmidt, 1981	Monte de Juan Tomé. La Laja	San Sebastián	La Gomera	Laurel forest	28.123022	-17.210137	1035
Dysdera silvatica Schmidt, 1981	Monte del Cedro	Hermigua	La Gomera	Laurel forest	28.119283	-17.237290	1260
Dysdera silvatica Schmidt, 1981	Pajarito	Hermigua	La Gomera	Laurel forest	28.108856	-17.241604	1339
Dysdera silvatica Schmidt, 1981	Pico de la Cruz	El Paso	La Palma	Dry subalpine scrub	28.756058	-17.855244	2297
Dysdera silvatica Schmidt, 1981	Pinar de Roque Faro	Garafía	La Palma	Pine forest	28.798556	-17.879149	1077
Dysdera silvatica Schmidt, 1981	Playa de Avalo	San Sebastián	La Gomera	Lowland xerophytic shrub (Pebbled beaches)	28.114684	-17.113168	34
Dysdera silvatica Schmidt, 1981	Road Juego de Bolas - Laguna Grande	Agulo	La Gomera	Laurel forest	28.129932	-17.254706	1186
Dysdera silvatica Schmidt, 1981	Roque de Los Muchachos (PNCT)	Garafía	La Palma	Dry subalpine scrub	28.760880	-17.885206	2261
Dysdera silvatica Schmidt, 1981	Roque Faro	Garafía	La Palma	Pine forest	28.798556	-17.879149	1076
Dysdera silvatica Schmidt, 1981	Tenerra (higher part). Taburiente National Park	El Paso	La Palma	Pine forest	28.719605	-17.899636	1178
Dysdera silvatica Schmidt, 1981	Teselinde. Ermita de Santa Clara	Vallehermoso	La Gomera	Laurel forest	28.196300	-17.287540	727
Dysdera simbeque Macías-Hernández & Arnedo, 2010	Cabecera Barranco Elvira Sánchez	Haría	Lanzarote	Thermo-sclerophyllous woodlands	29.130724	-13.516903	566
Dysdera simbeque Macías-Hernández & Arnedo, 2010	Fuente Ovejas. Guinate	Haría	Lanzarote	Thermo-sclerophyllous woodlands	29.184361	-13.501313	348
Dysdera spinidorsum Wunderlich, 1992	Cuchillete Montaña del Peños	Antigua	Fuerteventura	Midland xerophytic shrub	28.277677	-13.950877	368
Dysdera spinidorsum Wunderlich, 1992	Cuchillos de Jacomar	Tuineje	Fuerteventura	Lowland xerophytic shrub	28.275507	-13.913101	51
Dysdera spinidorsum Wunderlich, 1992	La Matilla	Puerto del Rosario	Fuerteventura	Lowland xerophytic shrub	28.561304	-13.954645	365
Dysdera spinidorsum Wunderlich, 1992	Montaña de la Cruz	Betancuria	Fuerteventura	Lowland xerophytic shrub	28.441619	-14.057354	592
Dysdera spinidorsum Wunderlich, 1992	Montaña Muda. La Matilla	Pto. del Rosario	Fuerteventura	Lowland xerophytic shrub	28.572092	-13.959406	677
Dysdera spinidorsum Wunderlich, 1992	Morro del Peñón. Tequital	Tuineje	Fuerteventura	Lowland xerophytic shrub	28.277250	-13.951061	398

Dysdera spinidorsum Wunderlich, 1992	Morro Tabaiba. Vallebrón	La Oliva	Fuerteventura	Lowland xerophytic shrub	28.592252	-13.943331	398
Dysdera spinidorsum Wunderlich, 1992	NE Carretera de Betancuria	Betancuria	Fuerteventura	Lowland xerophytic shrub	28.415214	-14.062498	364
Dysdera spinidorsum Wunderlich, 1992	Tegú (road Antigua-Betancuria)	Betancuria	Fuerteventura	Lowland xerophytic shrub	28.430451	-14.046955	639
Dysdera tilosensis Wunderlich, 1992	Andén Verde	Artenara	Gran Canaria	Lowland xerophytic shrub	28.035189	-15.746346	677
Dysdera tilosensis Wunderlich, 1992	Barranco de Guayadeque	Agüimes	Gran Canaria	Lowland xerophytic shrub	27.932980	-15.480070	550
Dysdera tilosensis Wunderlich, 1992	Barranco de La Virgen, over Valsendero	Valleseco	Gran Canaria	Laurel forest	28.046310	-15.588402	870
Dysdera tilosensis Wunderlich, 1992	Barranco de Veneguera	Mogán	Gran Canaria	Lowland xerophytic shrub	27.852500	-15.778550	61
Dysdera tilosensis Wunderlich, 1992	Barranco del Andén	Valleseco	Gran Canaria	Pine forest	28.025130	-15.606750	1535
Dysdera tilosensis Wunderlich, 1992	Barranco El Draguillo	Ingenio	Gran Canaria	Lowland xerophytic shrub	27.941568	-15.429992	200
Dysdera tilosensis Wunderlich, 1992	Barranco Oscuro	Valleseco	Gran Canaria	Laurel forest	28.067250	-15.589010	767
Dysdera tilosensis Wunderlich, 1992	Brezal del Palmital	Santa María de Guía	Gran Canaria	Laurel forest	28.111450	-15.601970	495
Dysdera tilosensis Wunderlich, 1992	Caideros	Gáldar	Gran Canaria	Thermo-sclerophyllous woodlands	28.075760	-15.649420	840
Dysdera tilosensis Wunderlich, 1992	Caldera de los Marteles	Telde	Gran Canaria	Thermo-sclerophyllous woodlands	27.952243	-15.527475	1536
Dysdera tilosensis Wunderlich, 1992	Campamento de Tamadaba	Agaete	Gran Canaria	Pine forest	28.056988	-15.690203	1212
Dysdera tilosensis Wunderlich, 1992	Cortijo San Gregorio. Tamaraceite	Las Palmas de GC	Gran Canaria	Thermo-sclerophyllous woodlands	28.085788	-15.491280	320
Dysdera tilosensis Wunderlich, 1992	Cruz de Tejeda	Tejeda	Gran Canaria	Pine forest	28.000616	-15.600653	1451
Dysdera tilosensis Wunderlich, 1992	Cumbre de Pajonales	Tejeda	Gran Canaria	Pine forest	27.943295	-15.678000	1280
Dysdera tilosensis Wunderlich, 1992	Degollada de Becerra	Tejeda	Gran Canaria	Pine forest	27.989730	-15.592920	1565
Dysdera tilosensis Wunderlich, 1992	Degollada de Las Brujas	Mogán	Gran Canaria	Pine forest	27.940540	-15.731890	1220
Dysdera tilosensis Wunderlich, 1992	Degollada de Tasartico	San Nicolás de Tolentino	Gran Canaria	Lowland xerophytic shrub	27.930860	-15.791130	315
Dysdera tilosensis Wunderlich, 1992	Fontanales	Moya	Gran Canaria	Thermo-sclerophyllous woodlands	28.055530	-15.608730	1050
Dysdera tilosensis Wunderlich, 1992	Forest road to Tirma	Artenara	Gran Canaria	Lowland xerophytic shrub	28.032133	-15.755400	610
Dysdera tilosensis Wunderlich, 1992	Llano del Garañón	Tejeda	Gran Canaria	Pine forest	27.958908	-15.590814	1640
Dysdera tilosensis Wunderlich, 1992	Llanos de la Pez	Tejeda	Gran Canaria	Pine forest	27.964312	-15.585547	1662
Dysdera tilosensis Wunderlich, 1992	Los Giles	Las Palmas de GC	Gran Canaria	Lowland xerophytic shrub	28.124012	-15.472221	165
Dysdera tilosensis Wunderlich, 1992	Los Majaletes	Ingenio	Gran Canaria	Thermo-sclerophyllous woodlands	27.940161	-15.499200	900
Dysdera tilosensis Wunderlich, 1992	Los Pechos	Vega de San Mateo	Gran Canaria	Pine forest	27.963134	-15.567380	1920
Dysdera tilosensis Wunderlich, 1992	Maspalomas	San Bartolome de Tirajana	Gran Canaria	Urban area	27.745670	-15.598780	6
Dysdera tilosensis Wunderlich, 1992	Montaña Cabreja	Vega de San Mateo	Gran Canaria	Thermo-sclerophyllous woodlands	28.010872	-15.539417	1005
Dysdera tilosensis Wunderlich, 1992	Montaña de Firgas	Firgas	Gran Canaria	Thermo-sclerophyllous woodlands	28.096288	-15.563653	650
Dysdera tilosensis Wunderlich, 1992	near Cruz de San Antonio	Mogán	Gran Canaria	Pine forest	27.916234	-15.691220	900
Dysdera tilosensis Wunderlich, 1992	Near Tirajana	San Bartolome de Tirajana	Gran Canaria	Pine forest	27.908870	-15.570200	930

Dysdera tilosensis Wunderlich, 1992	Pico Viento	Gáldar	Gran Canaria	Lowland xerophytic shrub	28.099788	-15.651685	805
Dysdera tilosensis Wunderlich, 1992	Pinar de Tamadaba	Agaete	Gran Canaria	Pine forest	28.031840	-15.677020	1134
Dysdera tilosensis Wunderlich, 1992	Presa de Cuevas Blancas	Valsequillo	Gran Canaria	Pine forest	27.964830	-15.545050	1661
Dysdera tilosensis Wunderlich, 1992	Presa de Las Niñas	Mogán	Gran Canaria	Pine forest	27.928312	-15.666048	881
Dysdera tilosensis Wunderlich, 1992	Roque Aguayro	Agüimes	Gran Canaria	Pine forest	27.884265	-15.476083	437
Dysdera tilosensis Wunderlich, 1992	Teror	Teror	Gran Canaria	Thermo-sclerophyllous woodlands	28.053790	-15.539000	715
Dysdera tilosensis Wunderlich, 1992	Tilos de Moya	Moya	Gran Canaria	Laurel forest	28.088752	-15.593203	529
Dysdera unguimmanis Ribera, Ferrández & Blasco, 1986	Cueva de Felipe Reventón	Icod de los Vinos	Tenerife	Lava tube	28.350180	-16.704638	612
Dysdera unguimmanis Ribera, Ferrández & Blasco, 1986	Cueva del Bucio. Aguamansa	La Orotava	Tenerife	Lava tube	28.360891	-16.498084	1078
Dysdera unguimmanis Ribera, Ferrández & Blasco, 1986	Cueva del Viento. Galería Ingleses	Icod de los Vinos	Tenerife	Lava tube	28.350010	-16.702820	625
Dysdera unguimmanis Ribera, Ferrández & Blasco, 1986	Cueva del Viento. Sobrado	Icod de los Vinos	Tenerife	Lava tube	28.345283	-16.698562	730
Dysdera verneaui Simon, 1883	Bajamar	La Laguna	Tenerife	Lowland xerophytic shrub	28.552515	-16.345881	25
Dysdera verneaui Simon, 1883	Barranco de Badajoz	Güímar	Tenerife	Laurel forest	28.303927	-16.440052	595
Dysdera verneaui Simon, 1883	Barranco de Las Cuevas	Buenavista	Tenerife	Lowland xerophytic shrub	28.317652	-16.831377	1047
Dysdera verneaui Simon, 1883	Barranco de los Cochinos. Monte del Agua	Los Silos	Tenerife	Laurel forest	28.324550	-16.819440	950
Dysdera verneaui Simon, 1883	Barranco de Nieto	Santa Cruz de Tenerife	Tenerife	Laurel forest	28.534070	-16.316255	793
Dysdera verneaui Simon, 1883	Barranco del Agua	Güímar	Tenerife	Laurel forest	28.307838	-16.448156	800
Dysdera verneaui Simon, 1883	Barranco del Pino	Santa Úrsula	Tenerife	Pine forest	28.384151	-16.475028	1375
Dysdera verneaui Simon, 1883	Barranco del Río	Arico	Tenerife	Pine forest	28.170842	-16.556740	905
Dysdera verneaui Simon, 1883	Base Zig-Zag. Teide National Park	La Orotava	Tenerife	Dry subalpine scrub	28.271970	-16.614630	2686
Dysdera verneaui Simon, 1883	Cabezo del Tejo	Santa Cruz de Tenerife	Tenerife	Laurel forest	28.565090	-16.165982	805
Dysdera verneaui Simon, 1883	Caldera de Pedro Gil	Arico	Tenerife	Pine forest	28.248283	-16.529917	1790
Dysdera verneaui Simon, 1883	Camino a Ichires. Anaga	La Laguna	Tenerife	Laurel forest	28.540023	-16.231988	830
Dysdera verneaui Simon, 1883	Camino La Ensillada - Chamorga	Anaga. La Laguna	Tenerife	Laurel forest	28.556209	-16.179828	800
Dysdera verneaui Simon, 1883	Cañada de Diego Hernández. Teide National Park	La Orotava	Tenerife	Dry subalpine scrub	28.277097	-16.551003	2095
Dysdera verneaui Simon, 1883	Cañadas del Teide	La Orotava	Tenerife	Dry subalpine scrub	28.288195	-16.573390	2184
Dysdera verneaui Simon, 1883	Chinobre	Santa Cruz de Tenerife	Tenerife	Laurel forest	28.559298	-16.173228	888
Dysdera verneaui Simon, 1883	Cruz del Carmen (path to the restaurant)	La Laguna	Tenerife	Laurel forest	28.531925	-16.279999	945
Dysdera verneaui Simon, 1883	Cueva Honda de Güímar	Güímar	Tenerife	Lava tube	28.310830	-16.370410	95
Dysdera verneaui Simon, 1883	Cumbre Bolico	Santiago del Teide	Tenerife	Laurel forest	28.249217	-16.528748	1953
Dysdera verneaui Simon, 1883	Cumbres de Arico. Trail from Izaña	Arico	Tenerife	Pine forest	28.249217	-16.528748	1950

Dysdera verneaui Simon, 1883	El Aderno, over Buenavista	Buenavista del Norte	Tenerife	Thermo-sclerophyllous woodlands	28.358258	-16.864456	200
Dysdera verneaui Simon, 1883	El Bailadero. Anaga	Santa Cruz de Tenerife	Tenerife	Laurel forest	28.550411	-16.203986	665
Dysdera verneaui Simon, 1883	El Guanche. Trail to Chimoche. Aguamansa	La Orotava	Tenerife	Pine forest	28.347186	-16.514020	1425
Dysdera verneaui Simon, 1883	El Moquinal	La Laguna	Tenerife	Laurel forest	28.537349	-16.309386	769
Dysdera verneaui Simon, 1883	El Moquinal	Santa Cruz de Tenerife	Tenerife	Laurel forest	28.537349	-16.309386	769
Dysdera verneaui Simon, 1883	El Pinalito	Vilaflor	Tenerife	Pine forest	28.183345	-16.636379	1866
Dysdera verneaui Simon, 1883	Forest road El Batán - Cruz del Carmen	La Laguna	Tenerife	Laurel forest	28.535320	-16.296800	880
Dysdera verneaui Simon, 1883	Forest road El Moquinal. Anaga	La Laguna	Tenerife	Laurel forest	28.537349	-16.309386	880
Dysdera verneaui Simon, 1883	Forest road Las Hiedras - Las Carboneras	La Laguna	Tenerife	Laurel forest	28.535600	-16.298810	950
Dysdera verneaui Simon, 1883	Forest road to Madre del Agua- Barranco del Río	Vilaflor	Tenerife	Pine forest	28.177310	-16.601628	1710
Dysdera verneaui Simon, 1883	Fuente de Mesa	Los Realejos	Tenerife	Pine forest	28.324291	-16.585643	1890
Dysdera verneaui Simon, 1883	Ijuana forest road	Santa Cruz de Tenerife	Tenerife	Laurel forest	28.560191	-16.169190	752
Dysdera verneaui Simon, 1883	Izaña	La Orotava	Tenerife	Dry subalpine scrub	28.306892	-16.514530	2237
Dysdera verneaui Simon, 1883	La Ensillada (Parking place). Anaga	Santa Cruz de Tenerife	Tenerife	Laurel forest	28.556191	-16.179817	800
Dysdera verneaui Simon, 1883	La Escalona	Adeje	Tenerife	Lowland xerophytic shrub	28.116650	-16.673350	985
Dysdera verneaui Simon, 1883	La Fortaleza. Teide National Park	La Orotava	Tenerife	Subalpine shrubs	28.316723	-16.591240	2070
Dysdera verneaui Simon, 1883	Las Lagunetas	El Rosario	Tenerife	Pine forest	28.418535	-16.410090	1400
Dysdera verneaui Simon, 1883	Las Lajas picnic place	Vilaflor	Tenerife	Pine forest	28.190338	-16.669163	2050
Dysdera verneaui Simon, 1883	Las Raíces	El Rosario	Tenerife	Pine forest	28.435580	-16.373500	1200
Dysdera verneaui Simon, 1883	Llano de los Viejos	La Laguna	Tenerife	Laurel forest	28.526709	-16.285000	772
Dysdera verneaui Simon, 1883	Llano de Ucanca. Teide National Park	La Orotava	Tenerife	Dry subalpine scrub	28.243303	-16.593732	2175
Dysdera verneaui Simon, 1883	Los Lomitos. Monte de Las Mesas	Santa Cruz de Tenerife	Tenerife	Semi-arid tropical shrubs	28.481365	-16.263627	390
Dysdera verneaui Simon, 1883	Madre del Agua	Granadilla	Tenerife	Pine forest	28.169368	-16.630688	1700
Dysdera verneaui Simon, 1883	Mirador del Moquinal	La Laguna	Tenerife	Laurel forest	28.537349	-16.309386	769
Dysdera verneaui Simon, 1883	Mirador sobre La Rosa de Piedra	La Orotava	Tenerife	Pine forest	28.339491	-16.524285	1514
Dysdera verneaui Simon, 1883	Montaña Chusqueros. Siete Cañadas	La Orotava	Tenerife	Subalpine shrubs	28.292240	-16.559003	2099
Dysdera verneaui Simon, 1883	Montaña de Los Conejos. Teide National Park	La Orotava	Tenerife	Dry subalpine scrub	28.279024	-16.606520	2424
Dysdera verneaui Simon, 1883	Montaña Las Lajas (beyond Boca Tauce)	Adeje	Tenerife	Pine forest	28.141800	-16.691400	1038
Dysdera verneaui Simon, 1883	Monte Aguirre	Santa Cruz de Tenerife	Tenerife	Laurel forest	28.529535	-16.268309	692
Dysdera verneaui Simon, 1883	Monte de Las Mercedes	La Laguna	Tenerife	Laurel forest	28.525678	-16.287059	776

Dysdera verneaui Simon, 1883	Monte de Santa Úrsula. La Corujera	Santa Úrsula	Tenerife	Laurel forest	28.403179	-16.486186	880
Dysdera verneaui Simon, 1883	Morada del Viento (Siete Fuentes road). Las Lagunetas	El Rosario	Tenerife	Pine forest	28.411180	-16.421127	1540
Dysdera verneaui Simon, 1883	Orticosa. Choza de la Loca	Arafo	Tenerife	Pine forest	28.384498	-16.447410	1450
Dysdera verneaui Simon, 1883	Over El Contador	Arico	Tenerife	Pine forest	28.197701	-16.531151	1050
Dysdera verneaui Simon, 1883	Pico Viejo. Teide National Park	La Orotava	Tenerife	Dry subalpine scrub	28.262998	-16.672482	3055
Dysdera verneaui Simon, 1883	Pine forest Roque Peral. Teide National Park	Los Realejos	Tenerife	Pine forest	28.308159	-16.576300	2000
Dysdera verneaui Simon, 1883	Punta del Hidalgo	La Laguna	Tenerife	Lowland xerophytic shrub	28.568108	-16.317760	86
Dysdera verneaui Simon, 1883	Retamar (3050 m). Teide National Park	La Orotava	Tenerife	Dry subalpine scrub	28.273448	-16.625179	3054
Dysdera verneaui Simon, 1883	Roque del Conde	Adeje	Tenerife	Xerophytic scrubs	28.093144	-16.698890	525
Dysdera verneaui Simon, 1883	Torre del Gaitero	Arafo	Tenerife	Pine forest	28.394697	-16.431984	1735
Dysdera verneaui Simon, 1883	Vilaflor	Vilaflor	Tenerife	Pine forest	28.176580	-16.643480	1719
Dysdera verneaui Simon, 1883	Vueltas de Taganana	Santa Cruz de Tenerife	Tenerife	Laurel forest	28.542082	-16.228833	833
Dysdera verneaui Simon, 1883	Zapata	La Laguna	Tenerife	Laurel forest	28.535499	-16.296200	889
Dysdera volcania Ribera, Ferrández & Blasco, 1985	Cueva de Felipe Reventón	Icod de los Vinos	Tenerife	Lava tube	28.350180	-16.704638	612
Dysdera volcania Ribera, Ferrández & Blasco, 1985	Cueva del Viento. Sobrado	Icod de los Vinos	Tenerife	Lava tube	28.345283	-16.698562	730
Dysdera yguanirae Arnedo & Ribera, 1997	Barranco del Andén	Valleseco	Gran Canaria	Pine forest	28.025130	-15.606750	1535
Dysdera yguanirae Arnedo & Ribera, 1997	Barranco Oscuro	Valleseco	Gran Canaria	Laurel forest	28.067250	-15.589010	767
Dysdera yguanirae Arnedo & Ribera, 1997	Brezal del Palmital	Santa María de Guía	Gran Canaria	Laurel forest	28.111450	-15.601970	495
Dysdera yguanirae Arnedo & Ribera, 1997	Caideros	Gáldar	Gran Canaria	Thermo-sclerophyllous woodlands	28.075760	-15.649420	840
