# Supplementary material for: A geographical distribution database of the genus Dysdera in the Canary Islands (Araneae, Dysderidae)
Source: Zookeys. 2016 Oct 19;(625):11–23. doi: 10.3897/zookeys.625.9847 (PMC5096360; doi:10.3897/zookeys.625.9847)
Supplement: Supplementary material 2 — Supplementary Figure 1 [file zookeys-625-011-s002.pdf]

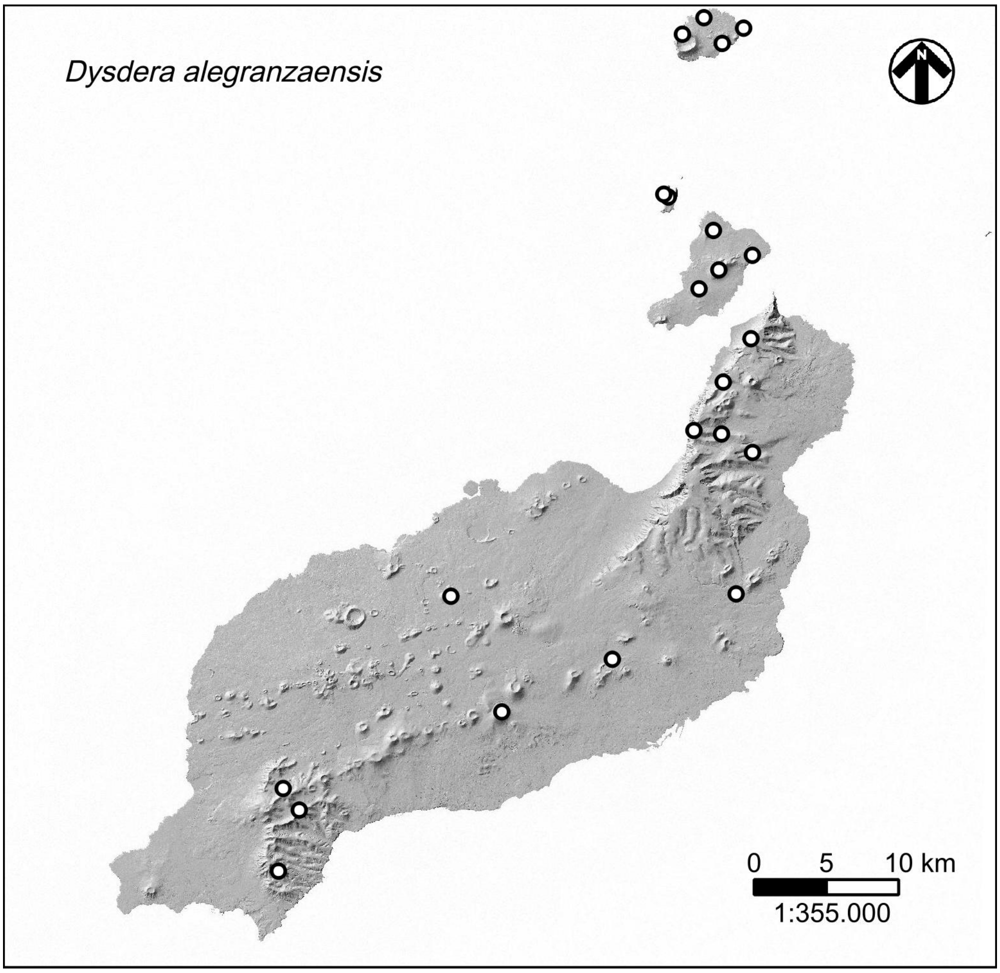
Dysdera alegranzaensis
0 5 10 km
1:355.000

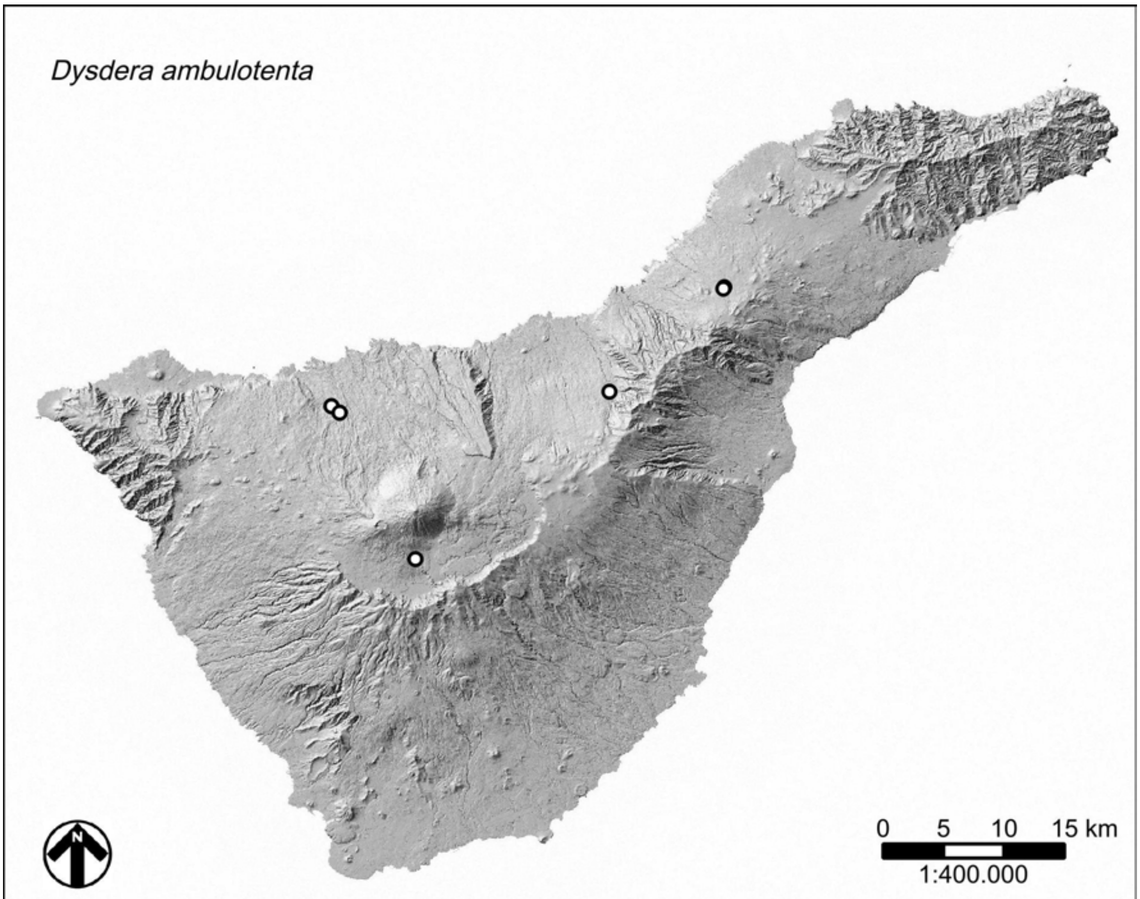
Dysdera ambulotenta
0 5 10 15 km
1:400.000

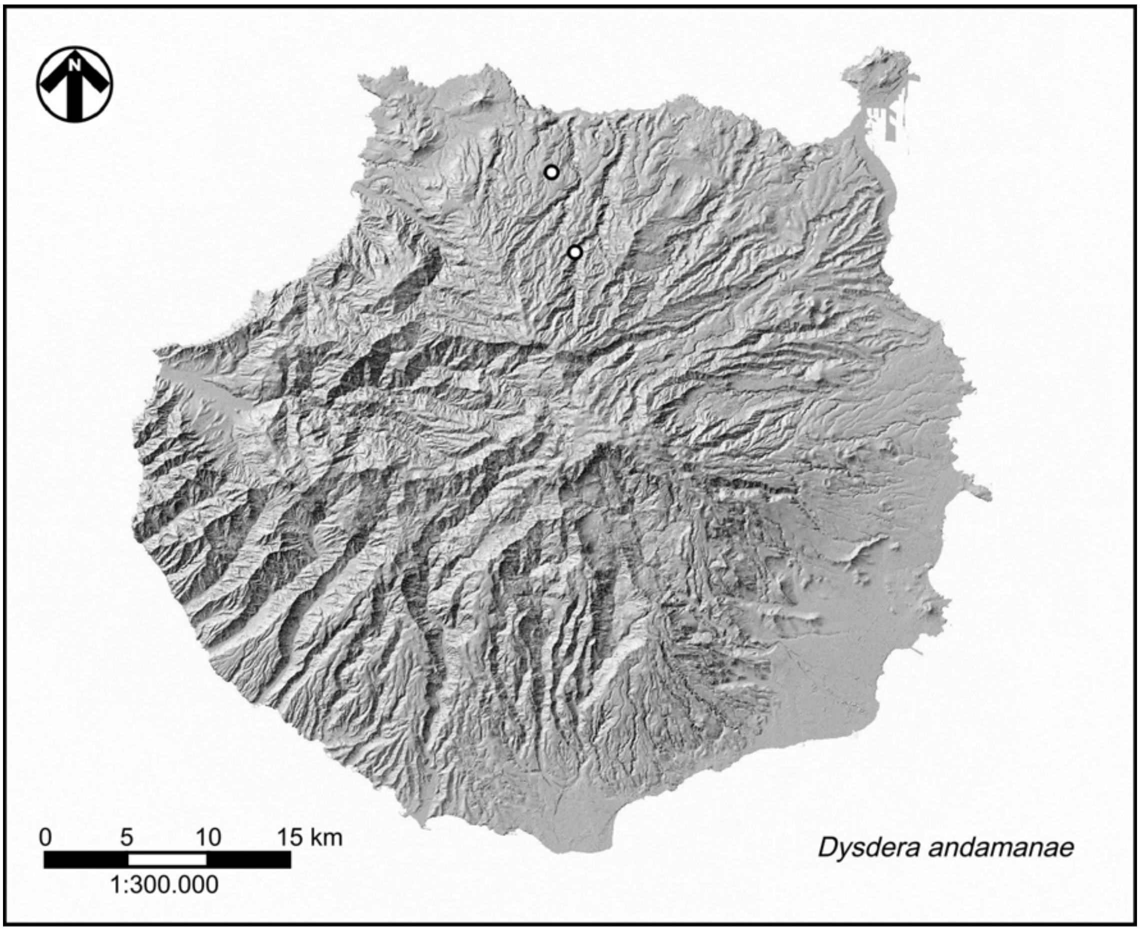
N
0 5 10 15 km
1:300.000
Dysdera andamanae

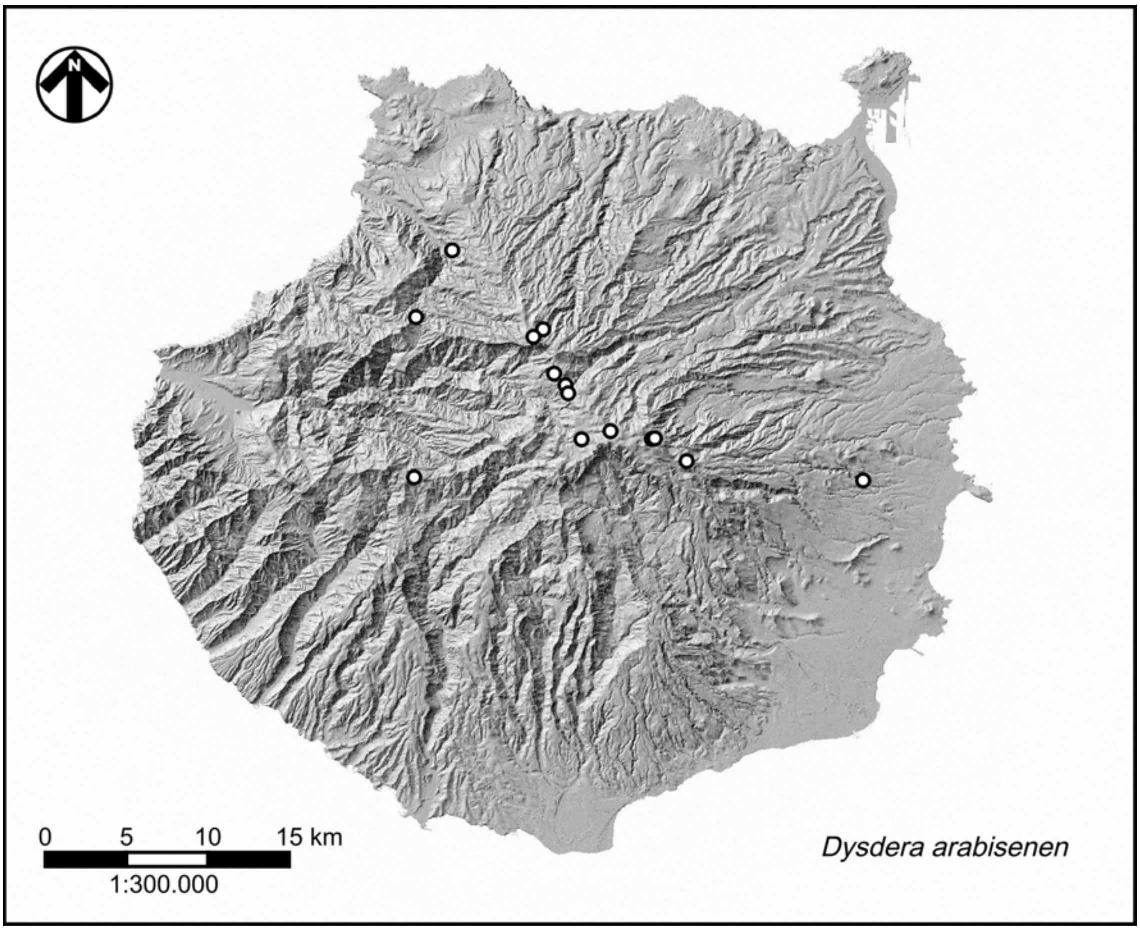
N
0 5 10 15 km
1:300.000
Dysdera arabisenen

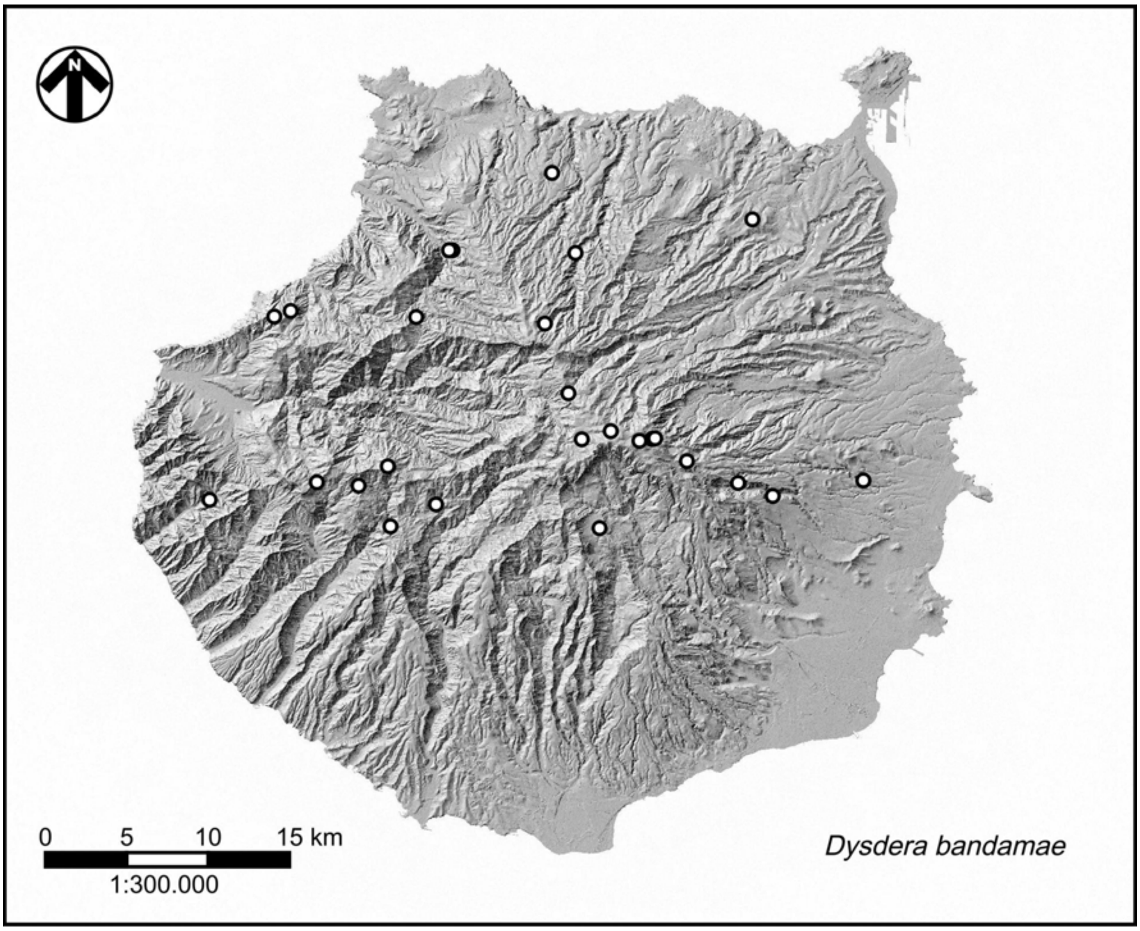
N
0 5 10 15 km
1:300.000
Dysdera bandamae

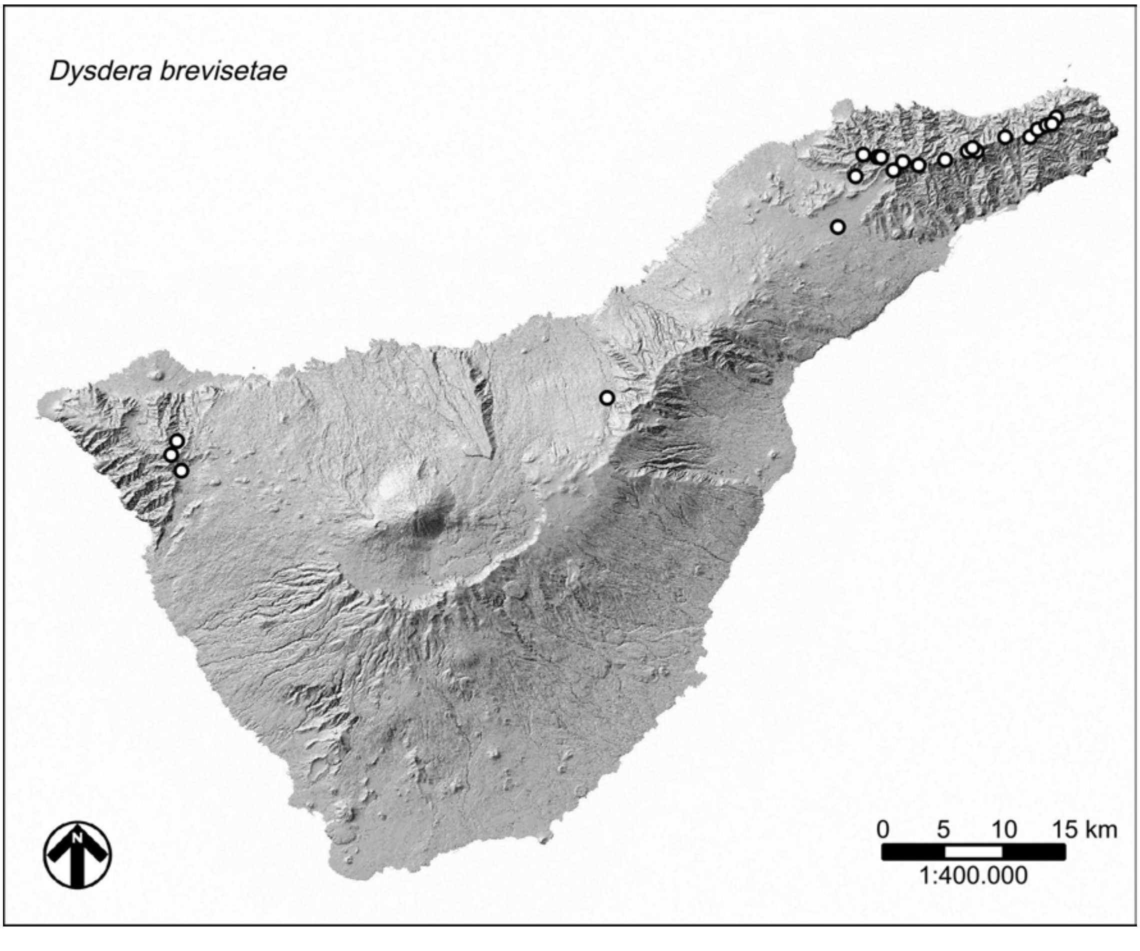
Dysdera brevisetae
0 5 10 15 km
1:400.000
N

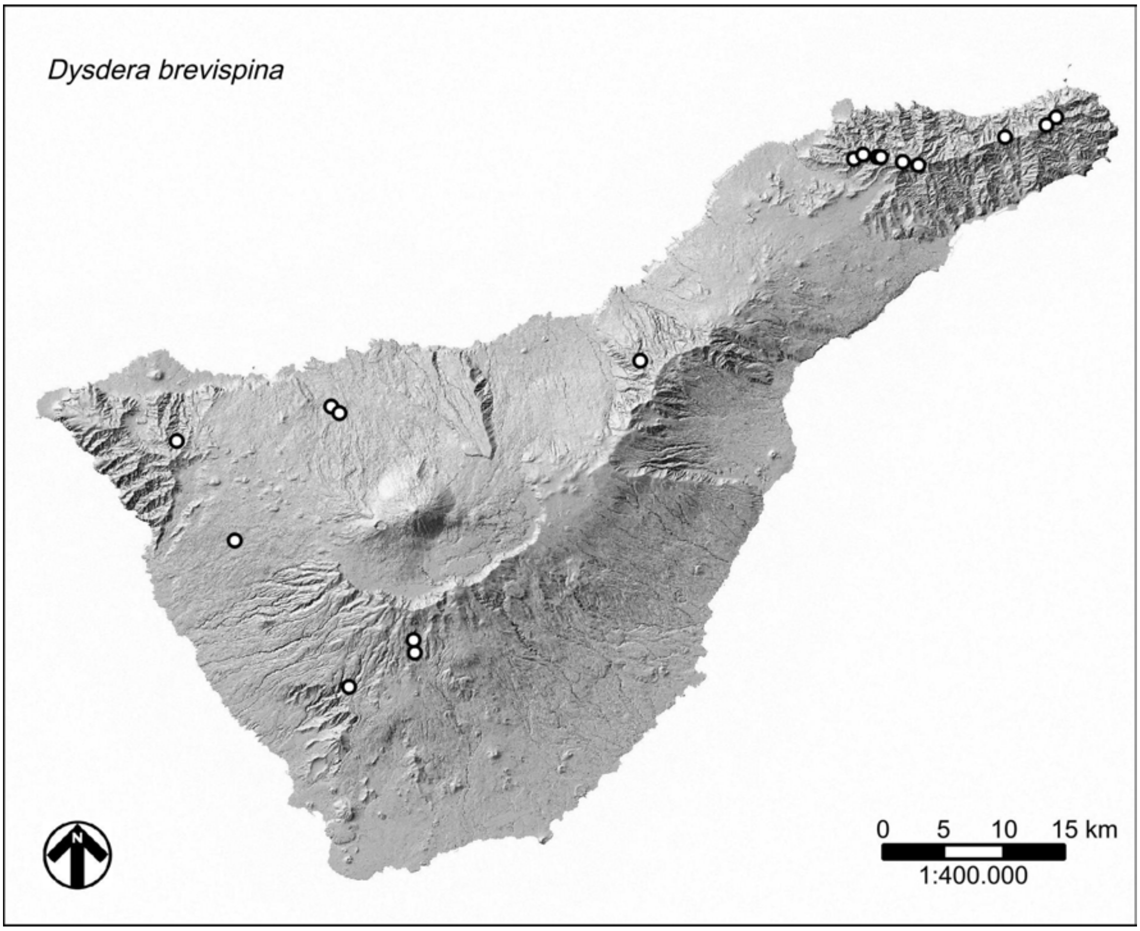
Dysdera brevispina
0 5 10 15 km
1:400.000

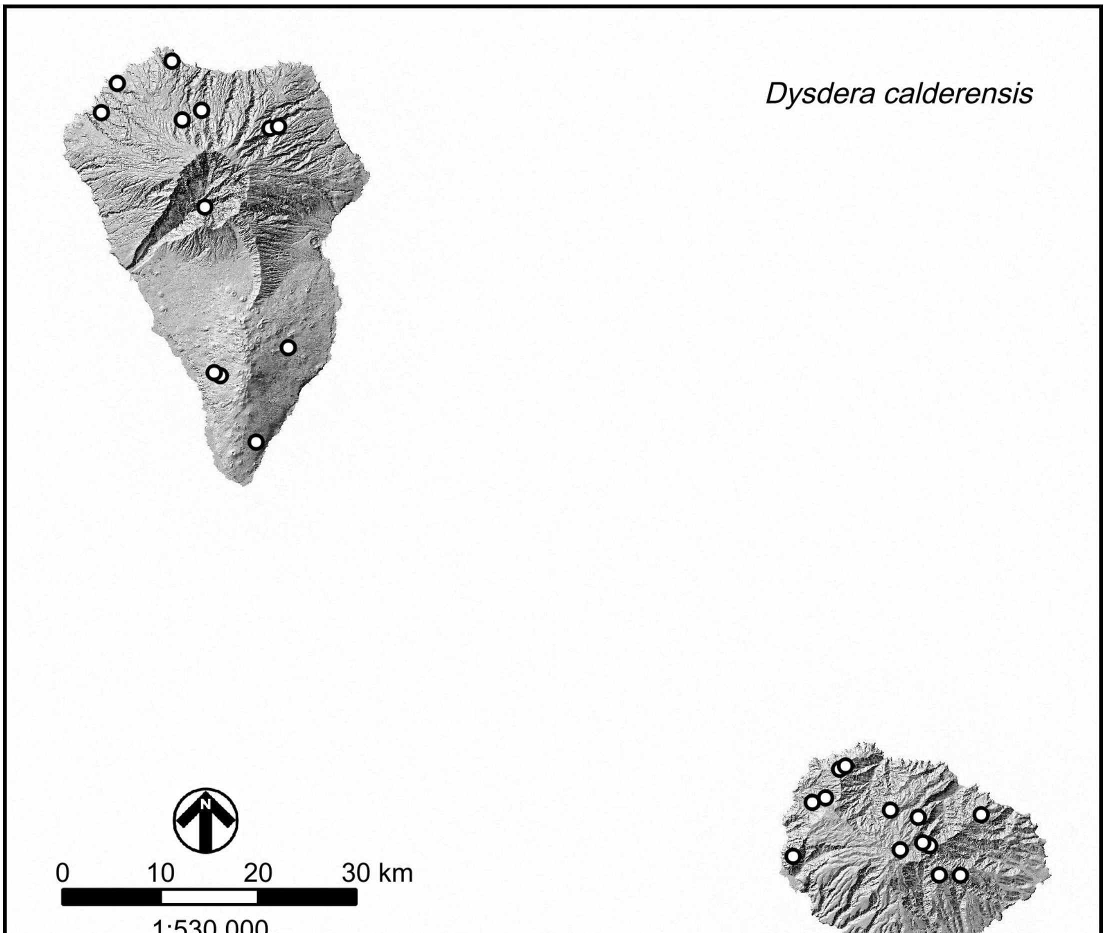
Dysdera calderensis
0 10 20 30 km
1:530.000

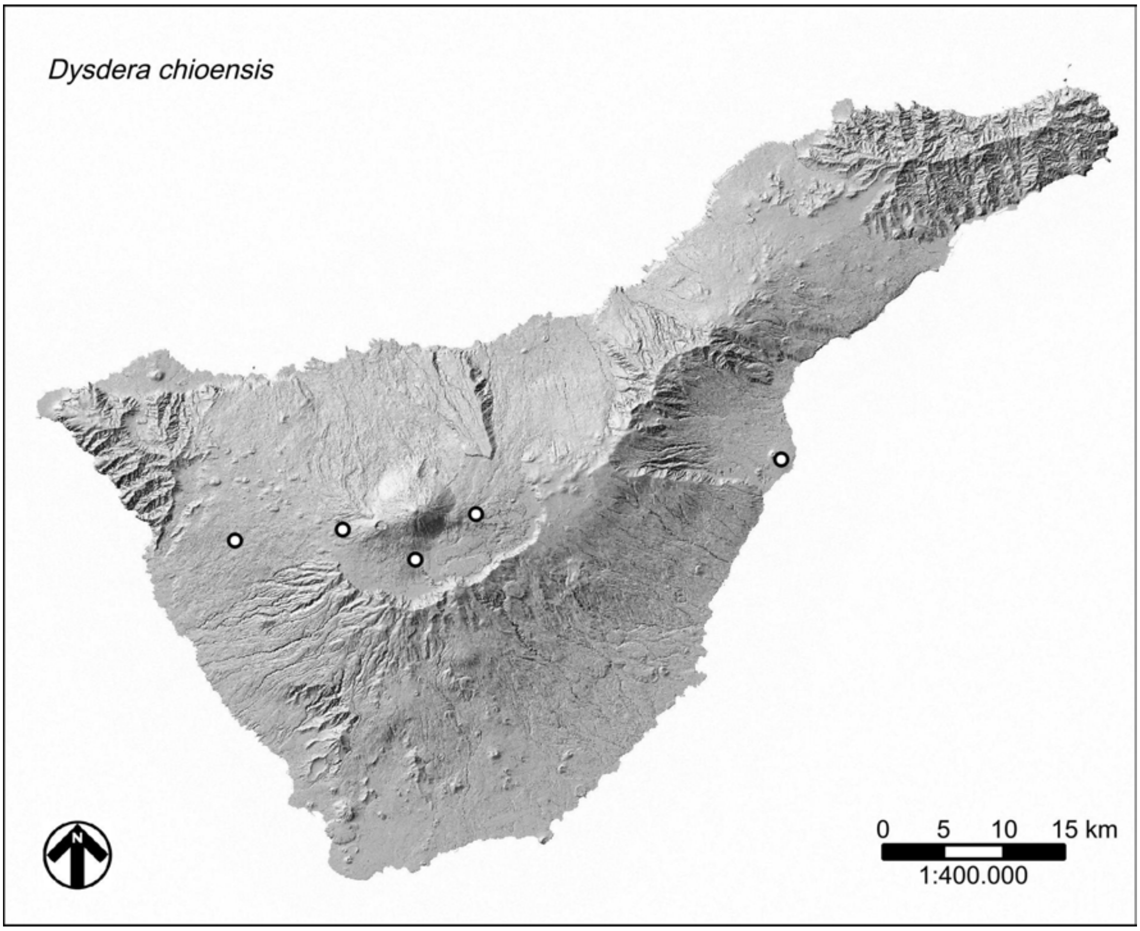
Dysdera chioensis
0 5 10 15 km
1:400.000
N

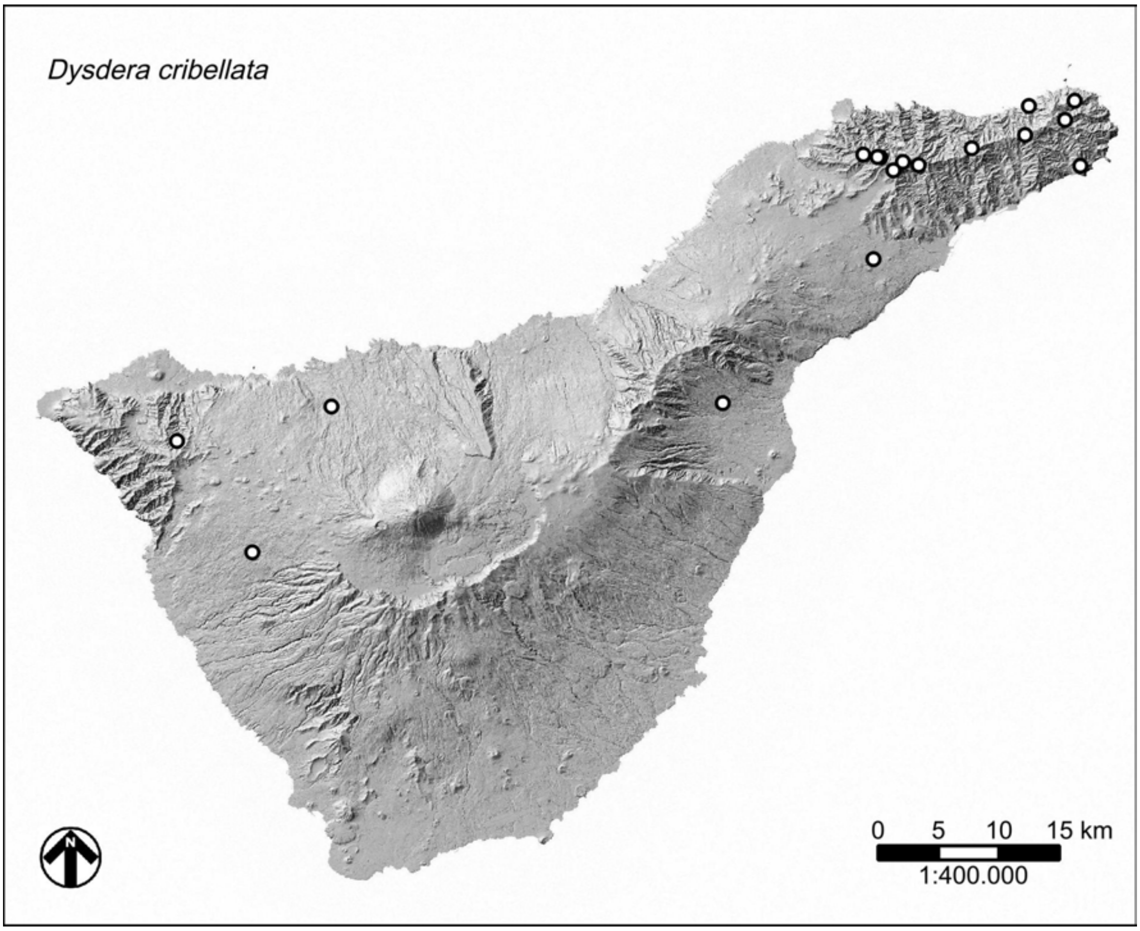
Dysdera cribellata
0 5 10 15 km
1:400.000
N

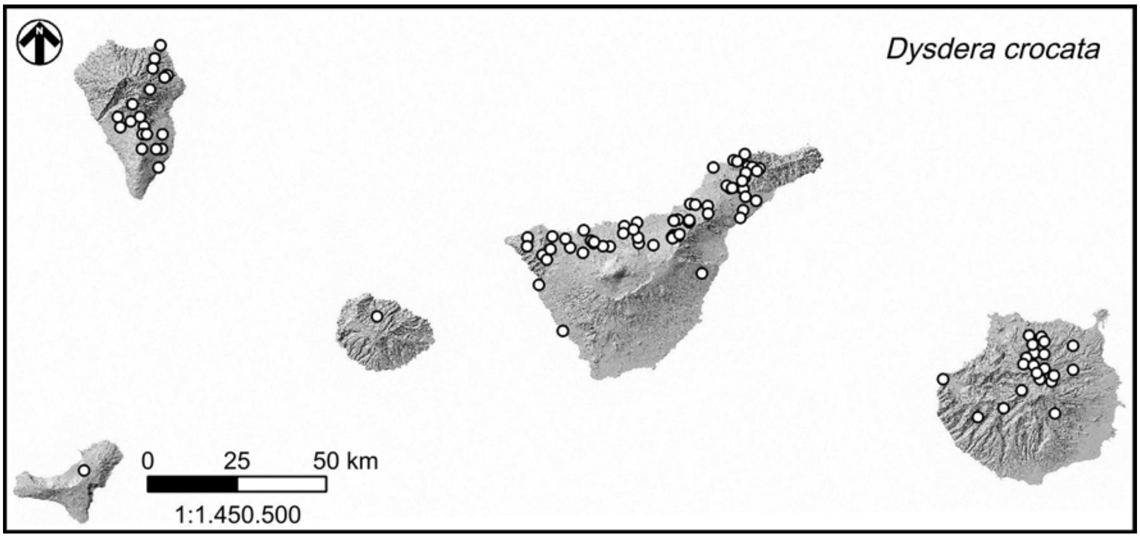
Dysdera crocata
0
25
50 km
1:1.450.500

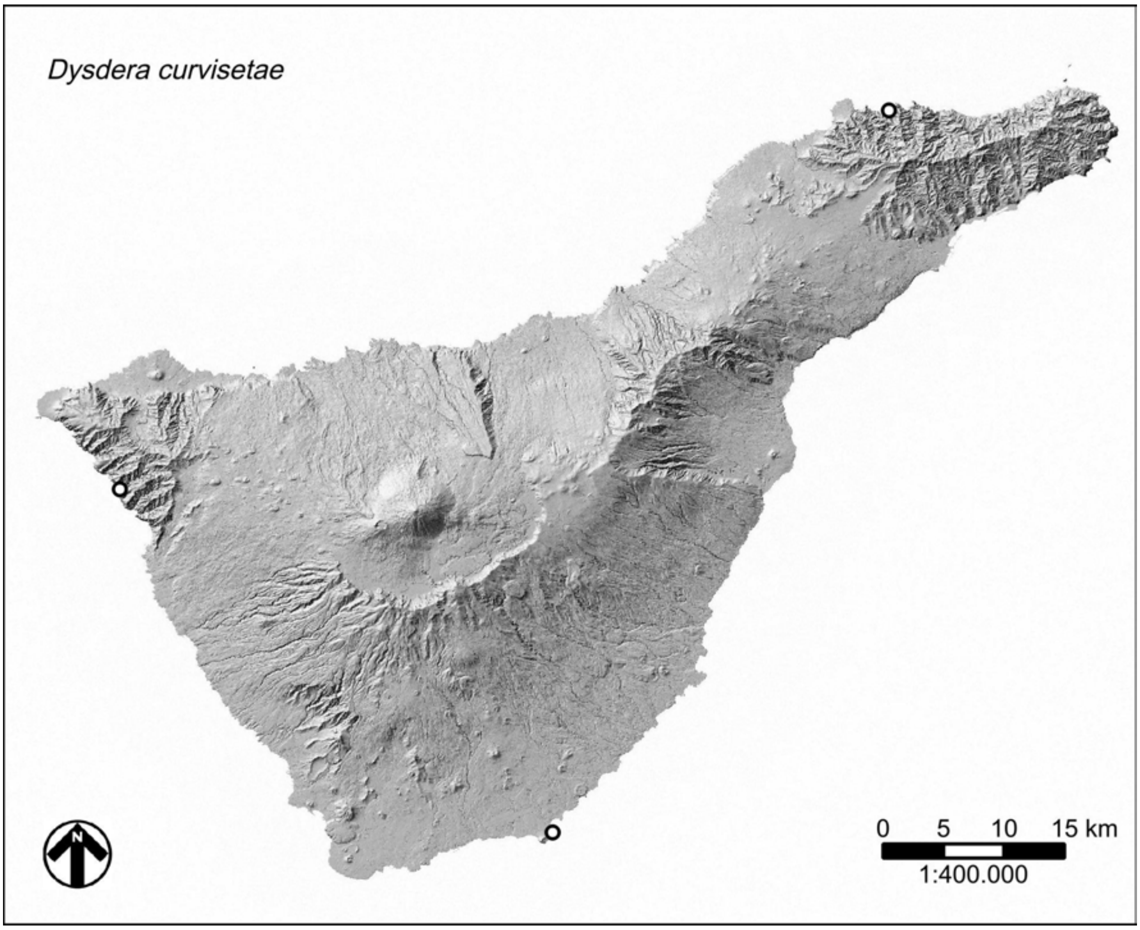
Dysdera curvisetae
0
5
10
15 km
1:400.000

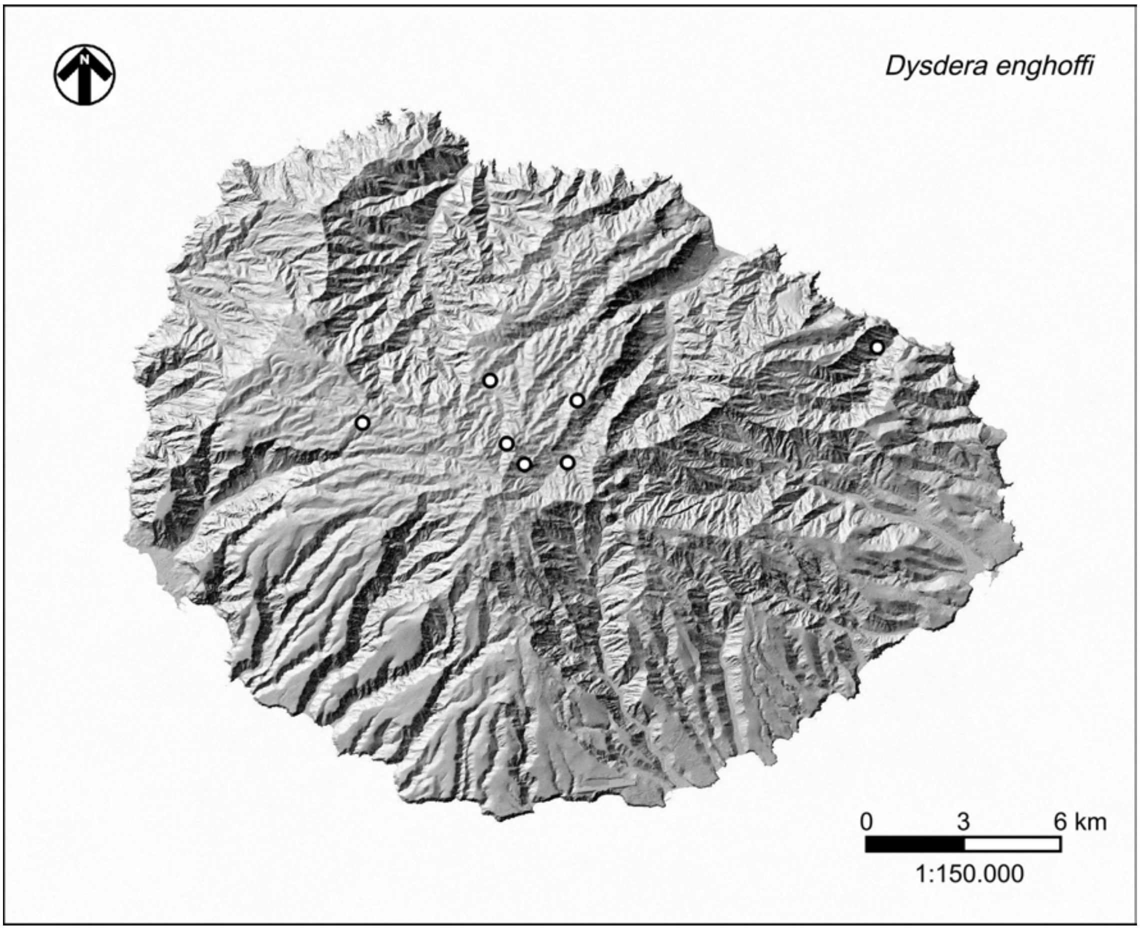
Dysdera enghoffi
N
0
3
6 km
1:150.000

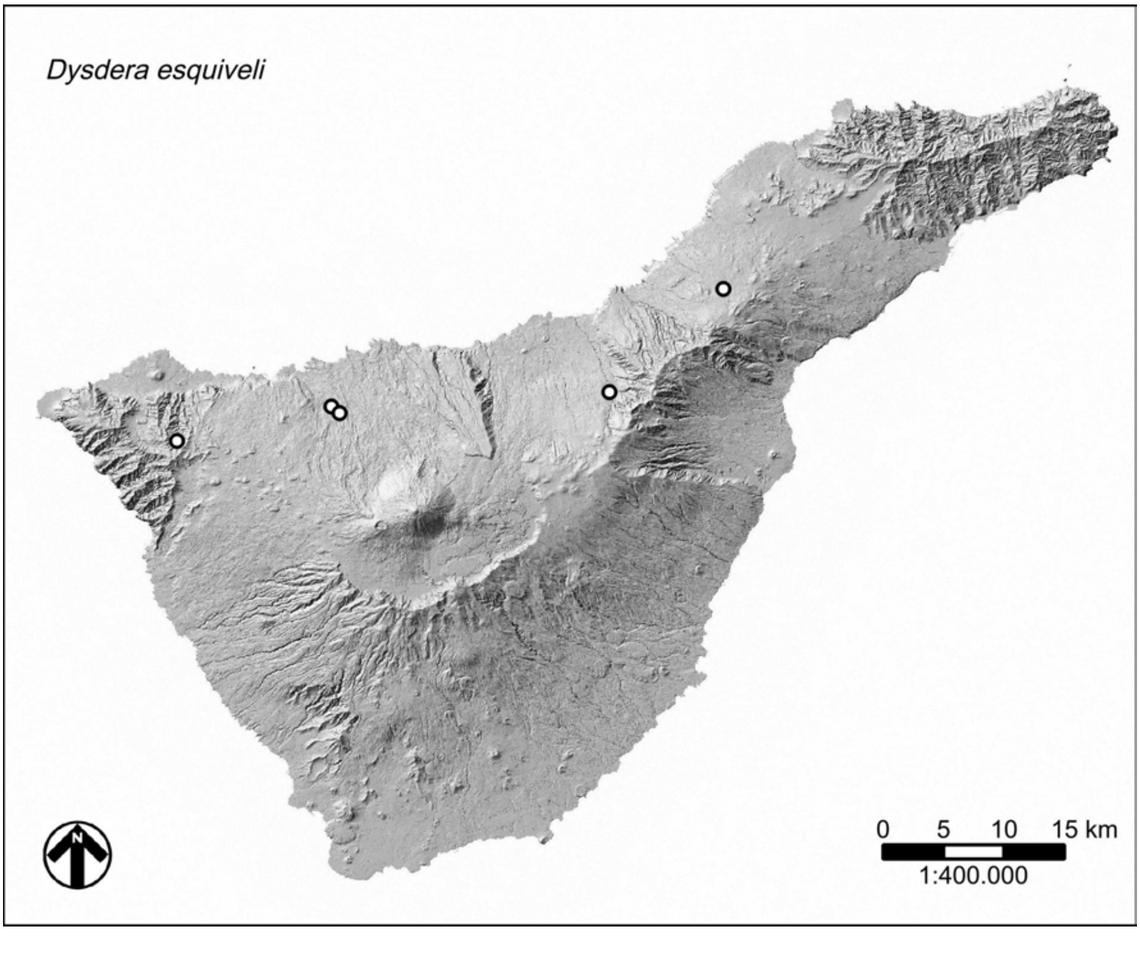
Dysdera esquiveli
N
0
5
10
15 km
1:400.000

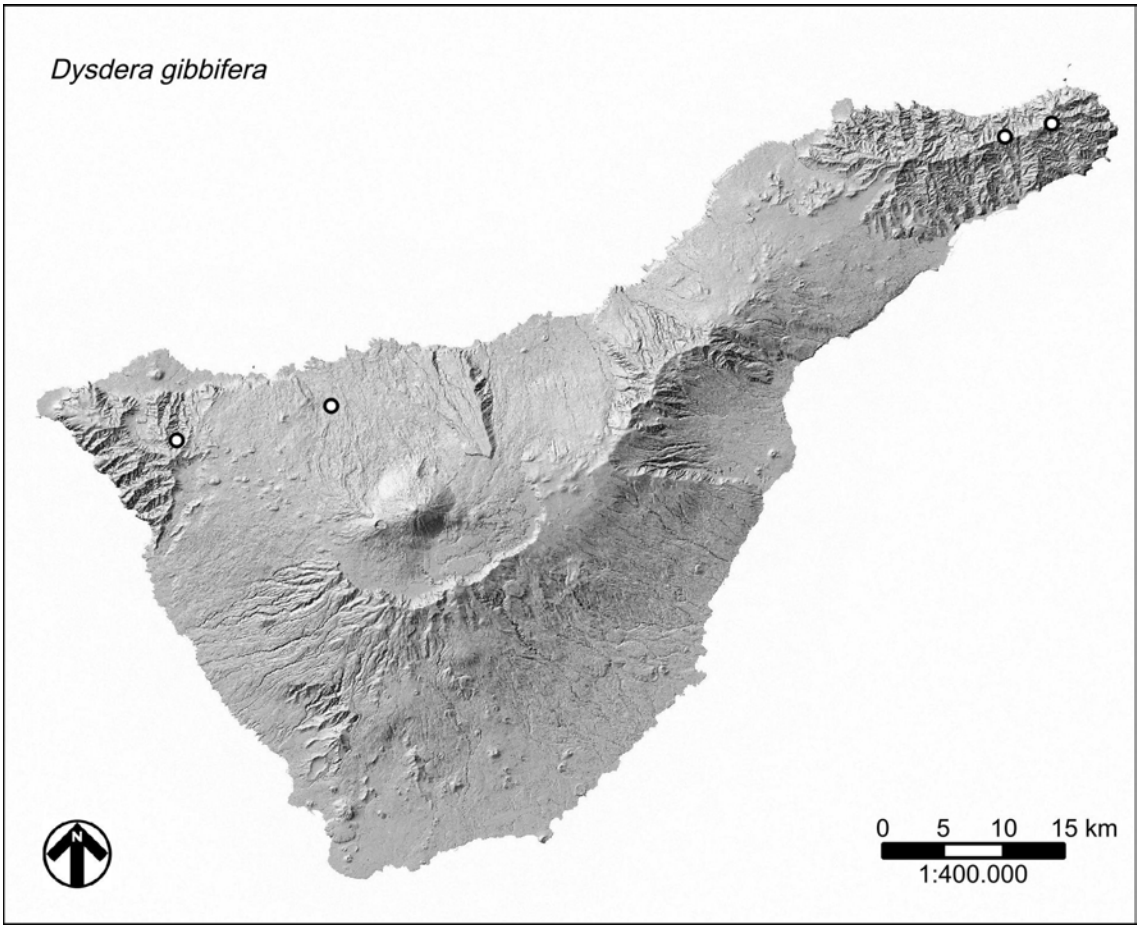
Dysdera gibbifera
0 5 10 15 km
1:400.000

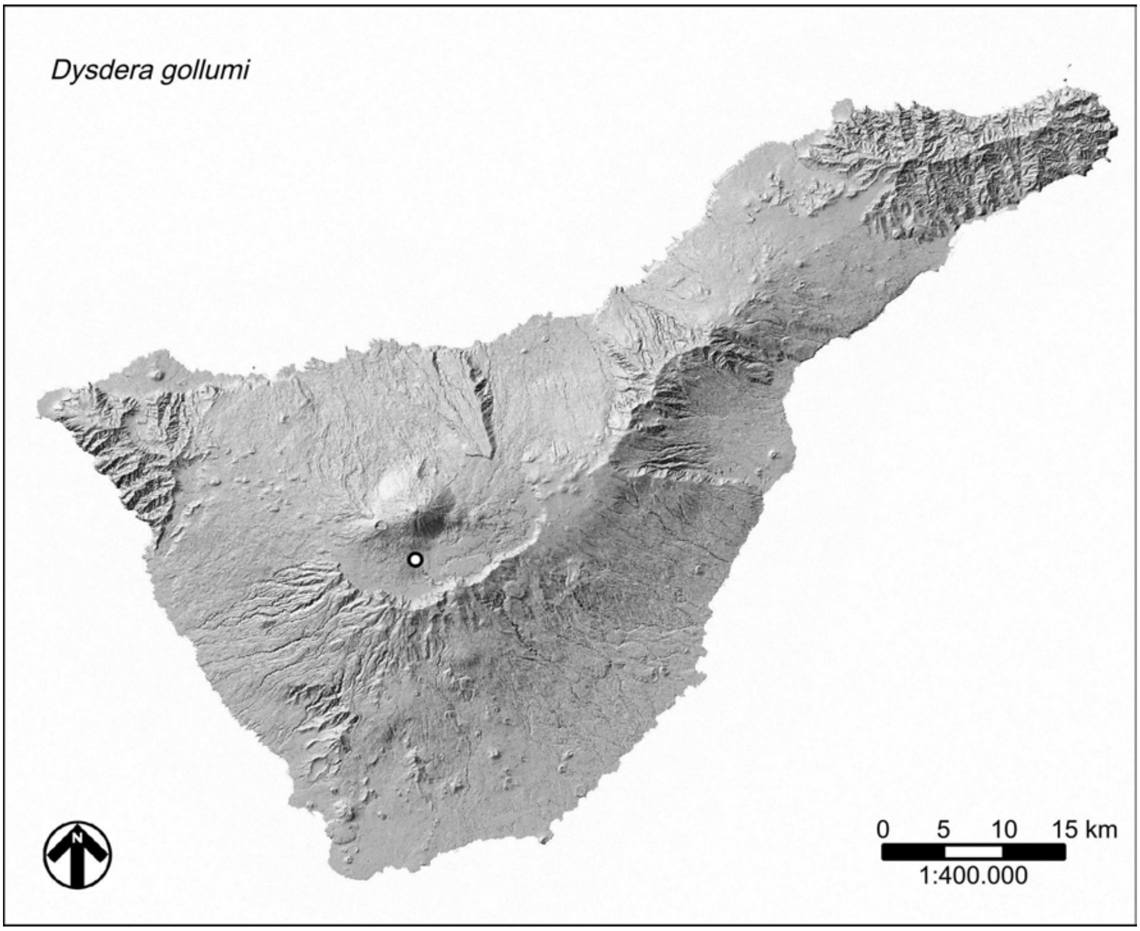
Dysdera gollumi
0 5 10 15 km
1:400.000

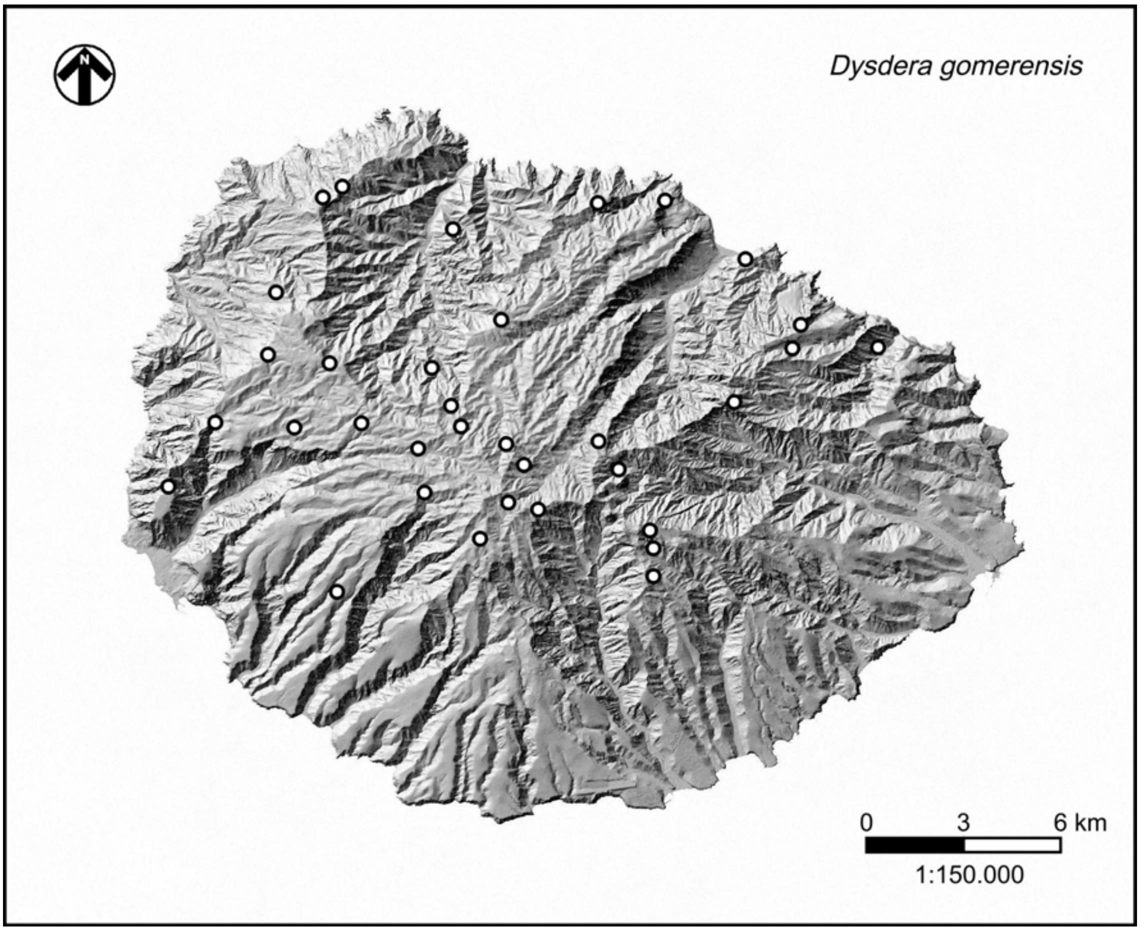
Dysdera gomerensis
0
3
6 km
1:150.000

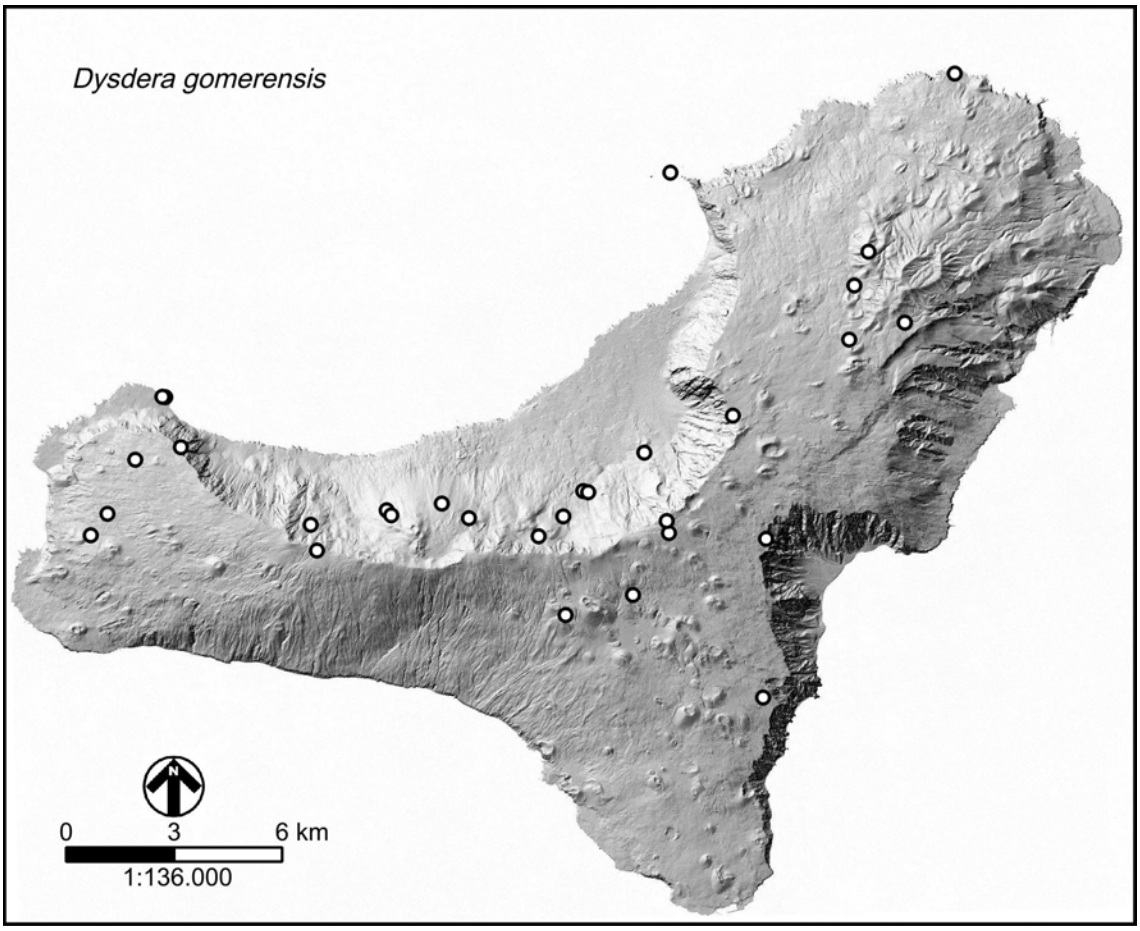
Dysdera gomerensis
0
3
6 km
1:136.000

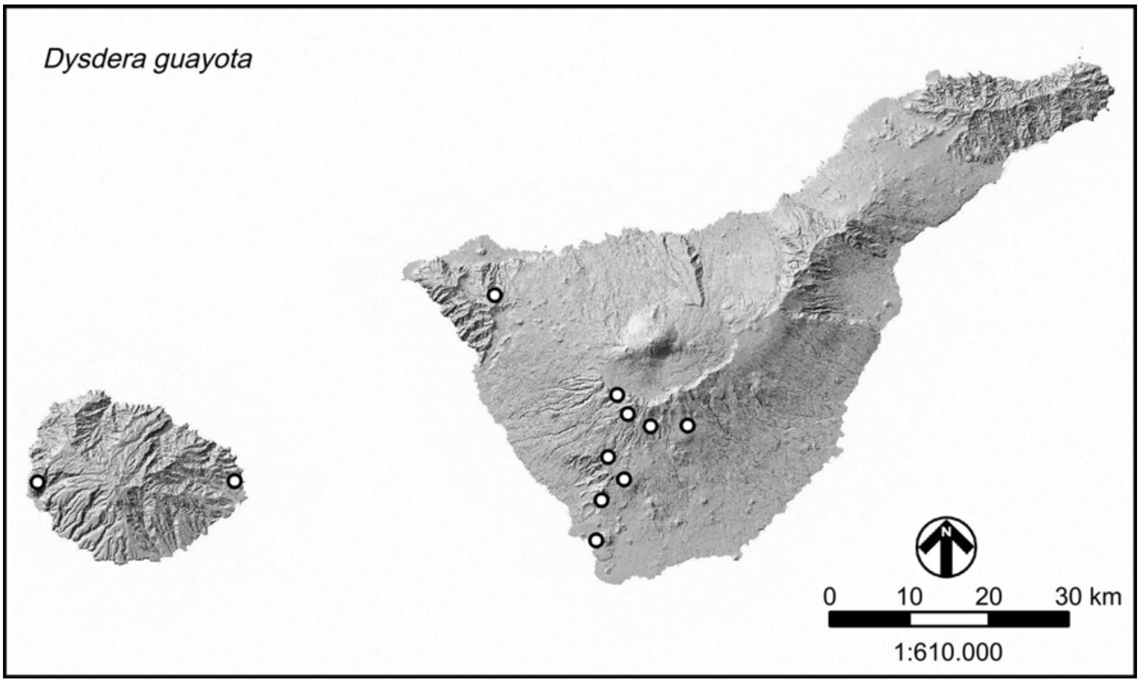
Dysdera guayota
N
0 10 20 30 km
1:610.000

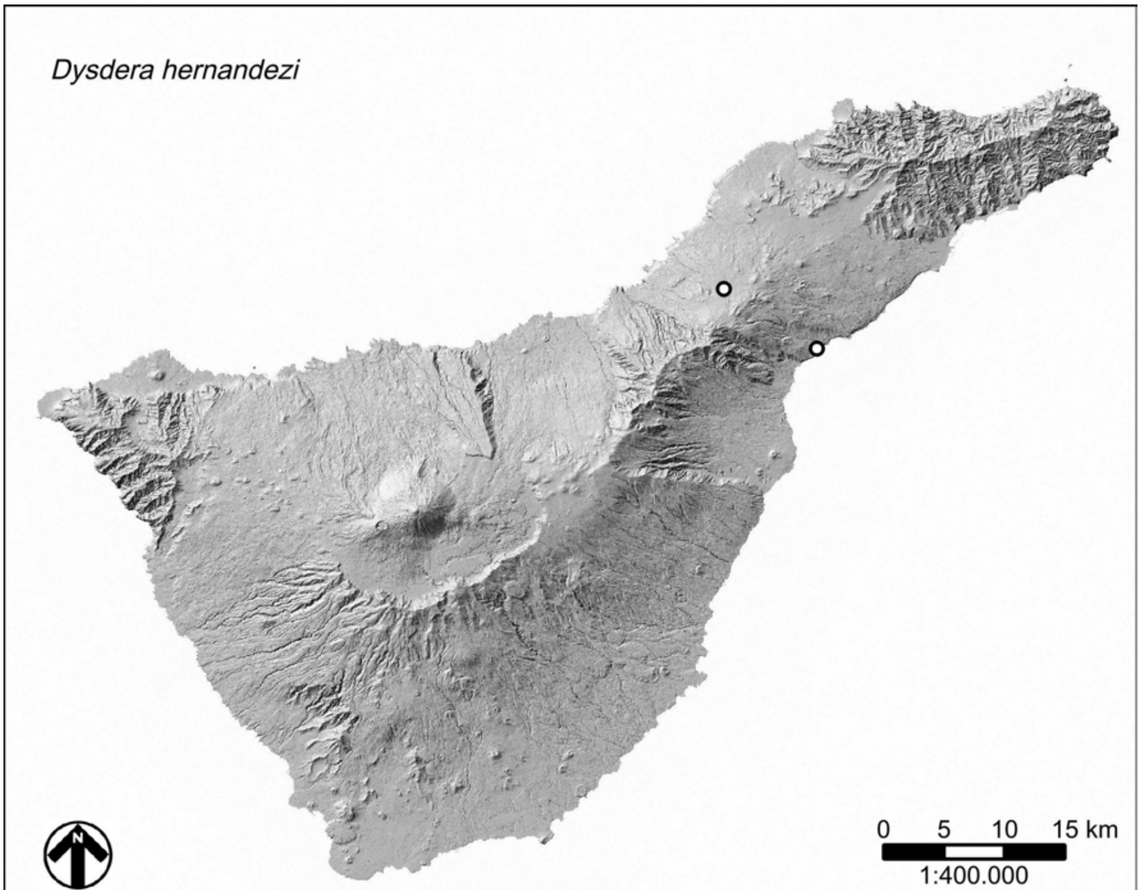
Dysdera hernandezi
N
0 5 10 15 km
1:400.000

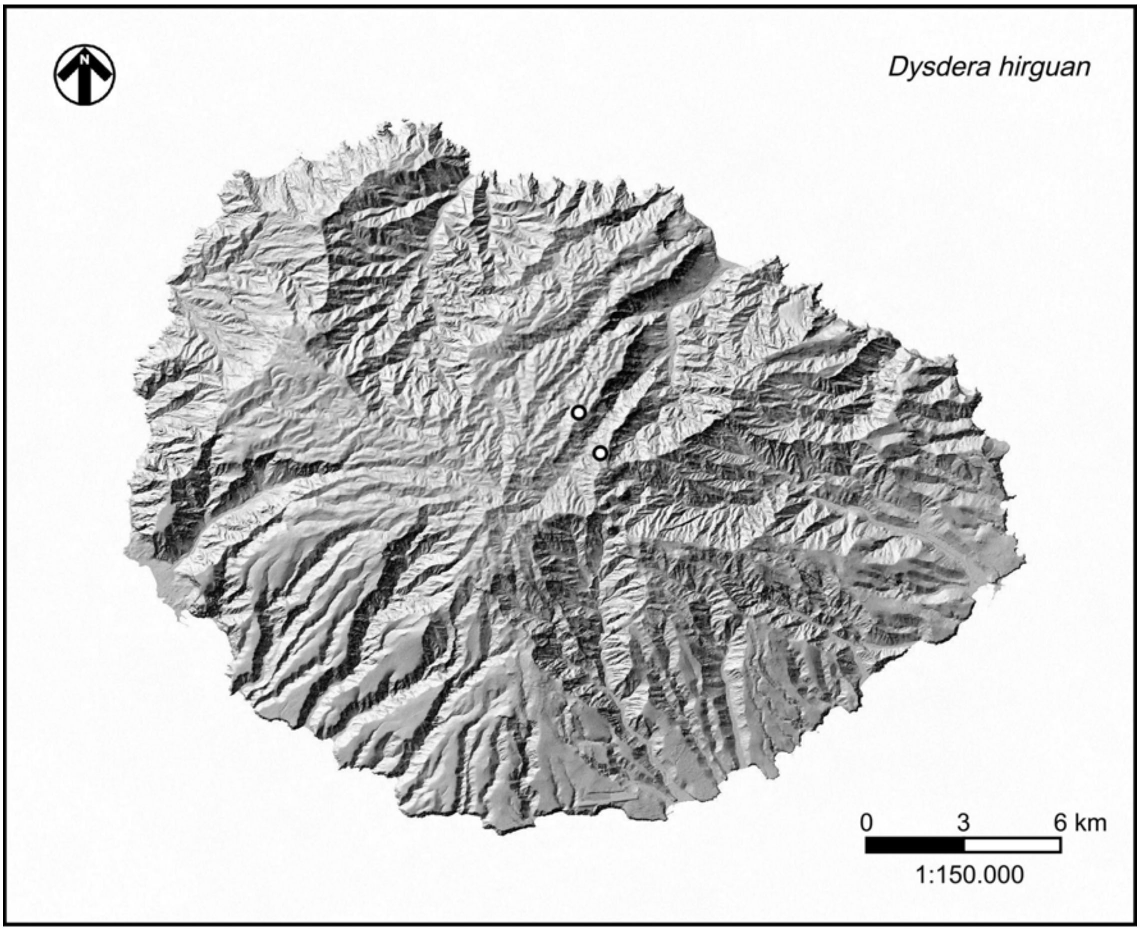
Dysdera hirguan
0 3 6 km
1:150.000

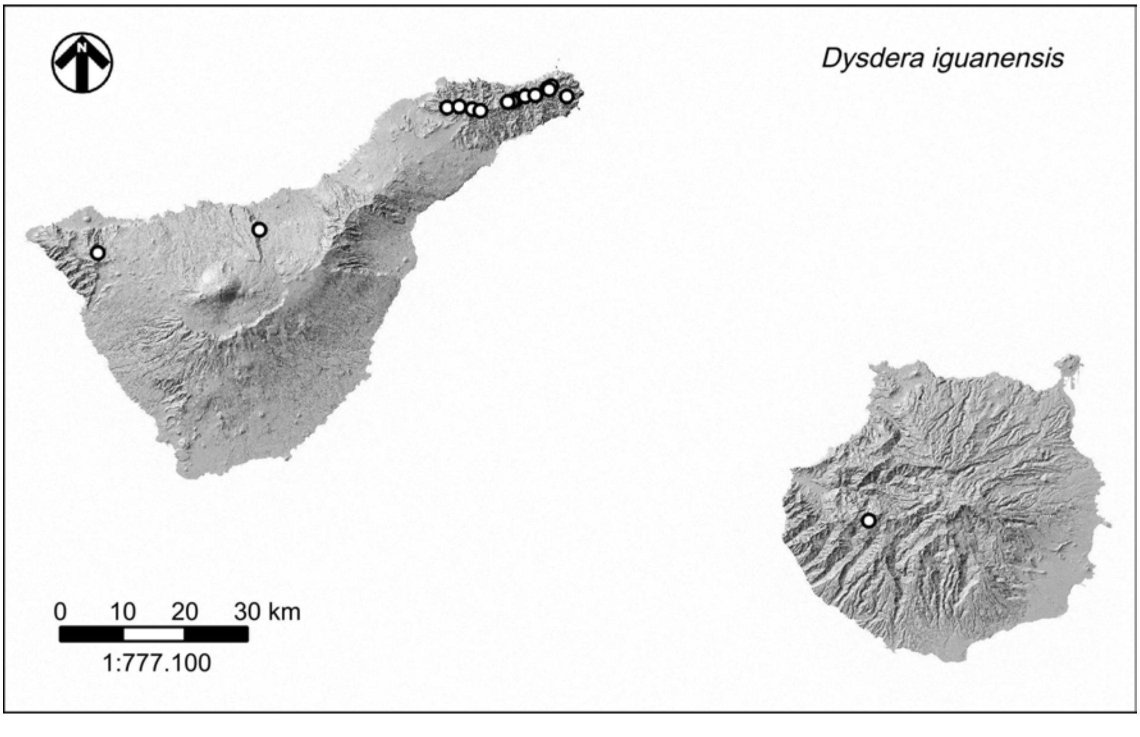
Dysdera iguanensis
0 10 20 30 km
1:777.100

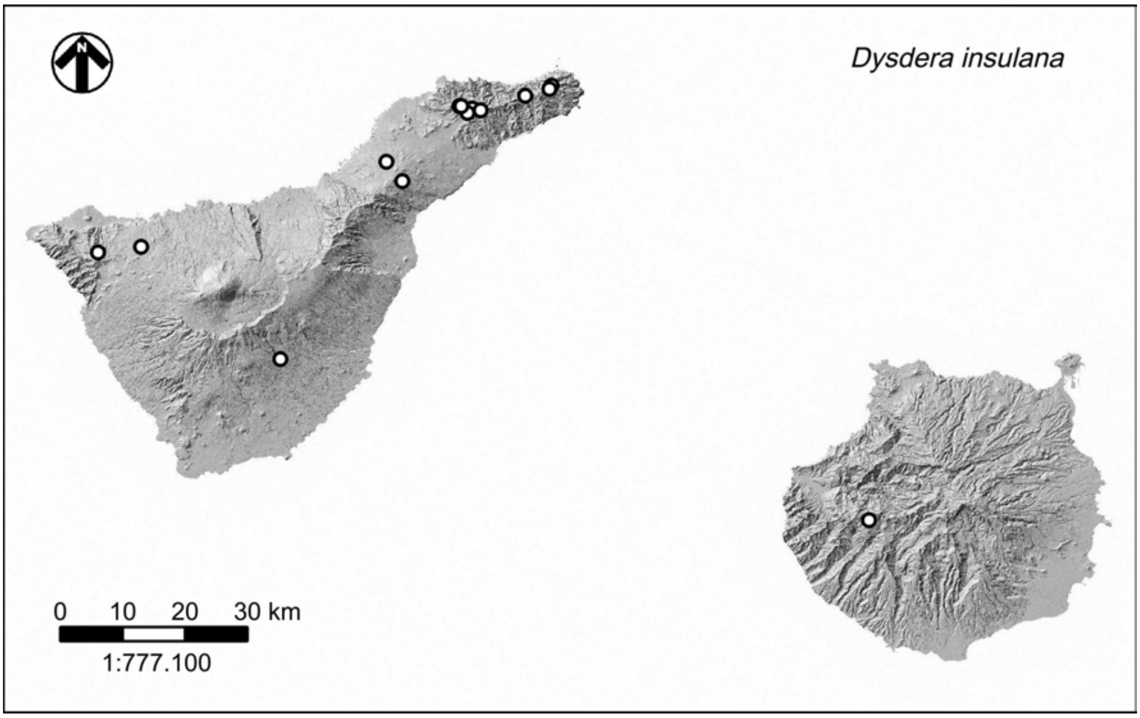

Dysdera insulana
N
0 10 20 30 km
1:777.100

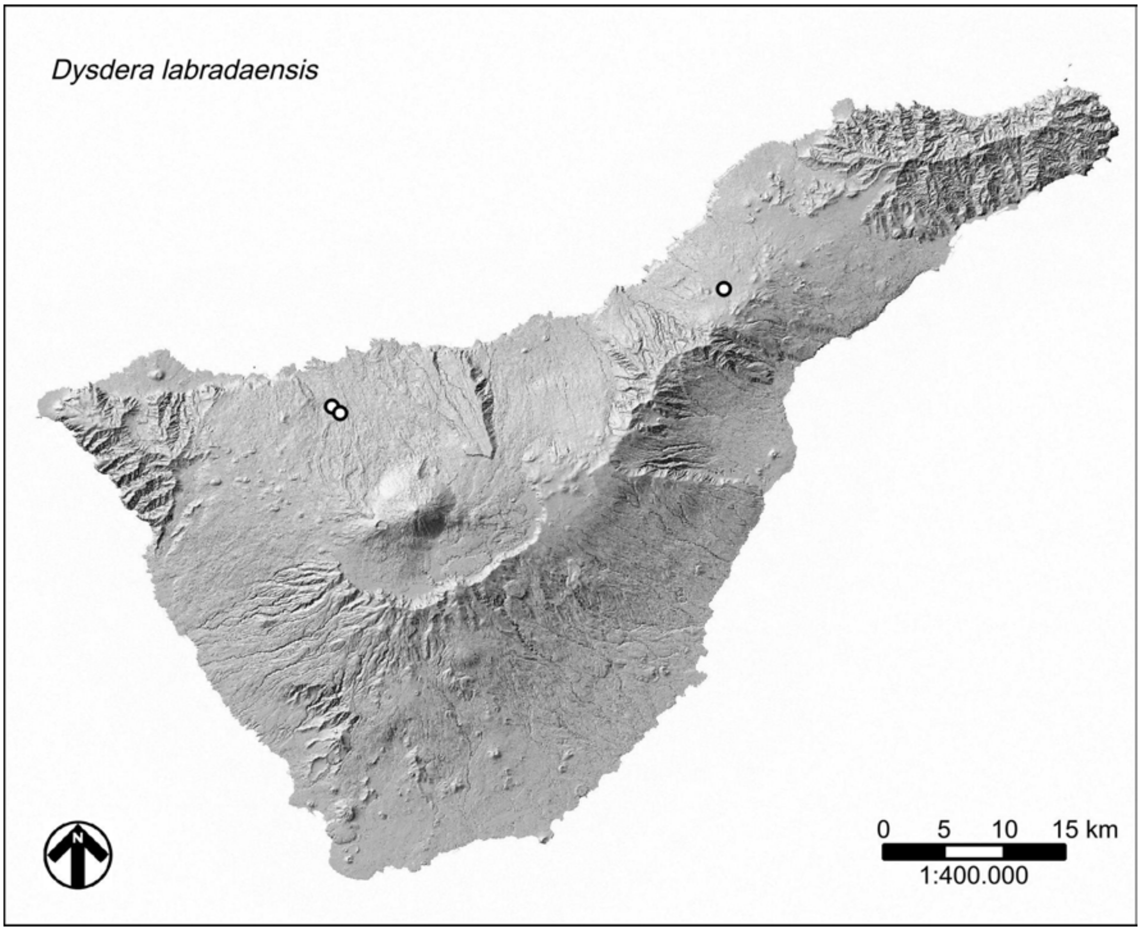

Dysdera labradaensis
N
0 5 10 15 km
1:400.000

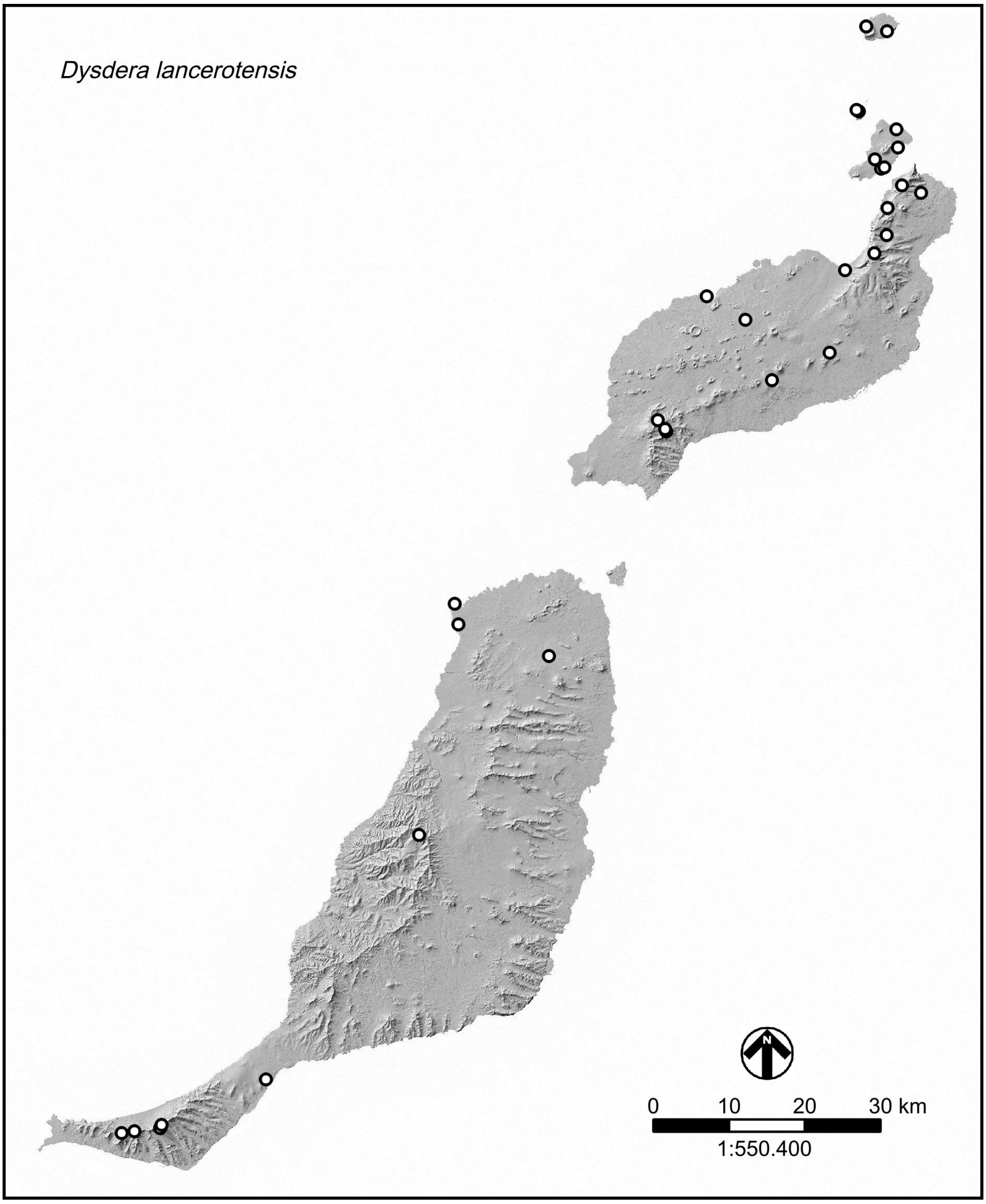
Dysdera lancerotensis
N
0 10 20 30 km
1:550.400

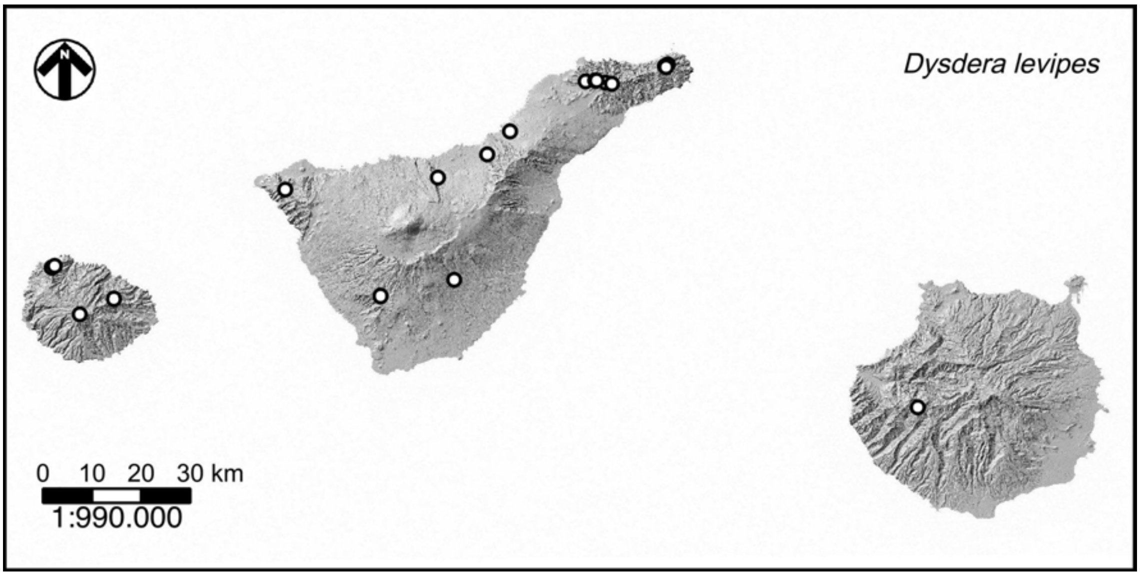
N
Dysdera levipes
0 10 20 30 km
1:990.000

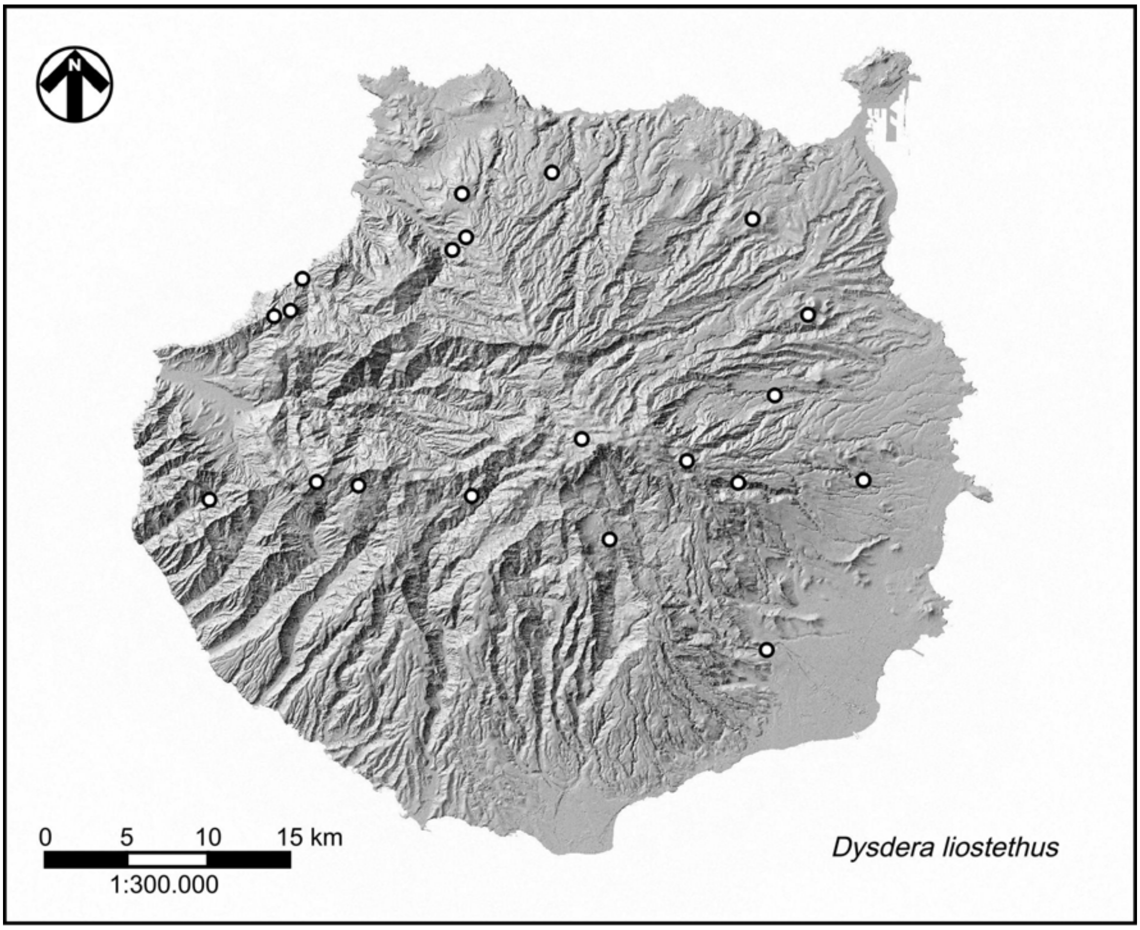
N
0 5 10 15 km
1:300.000
Dysdera liostethus

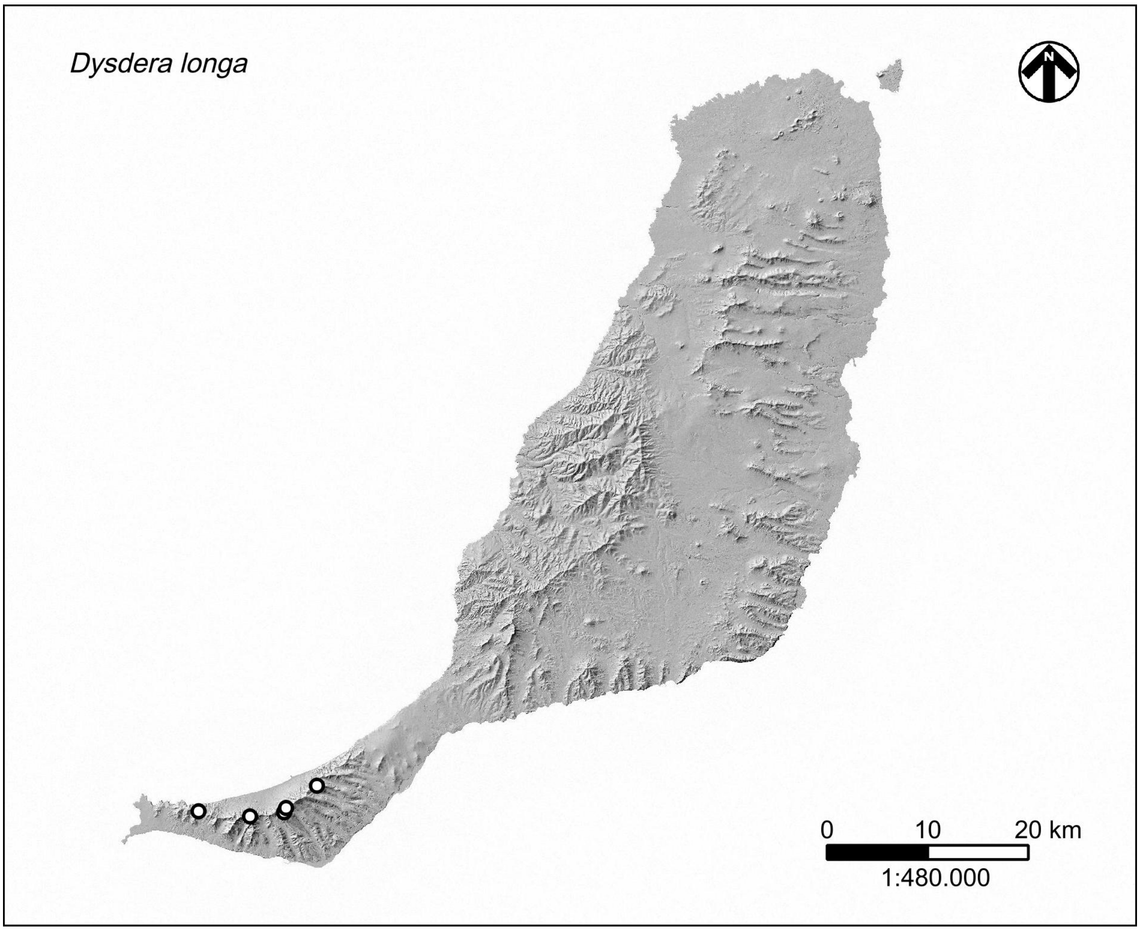
Dysdera longa
N
0 10 20 km
1:480.000

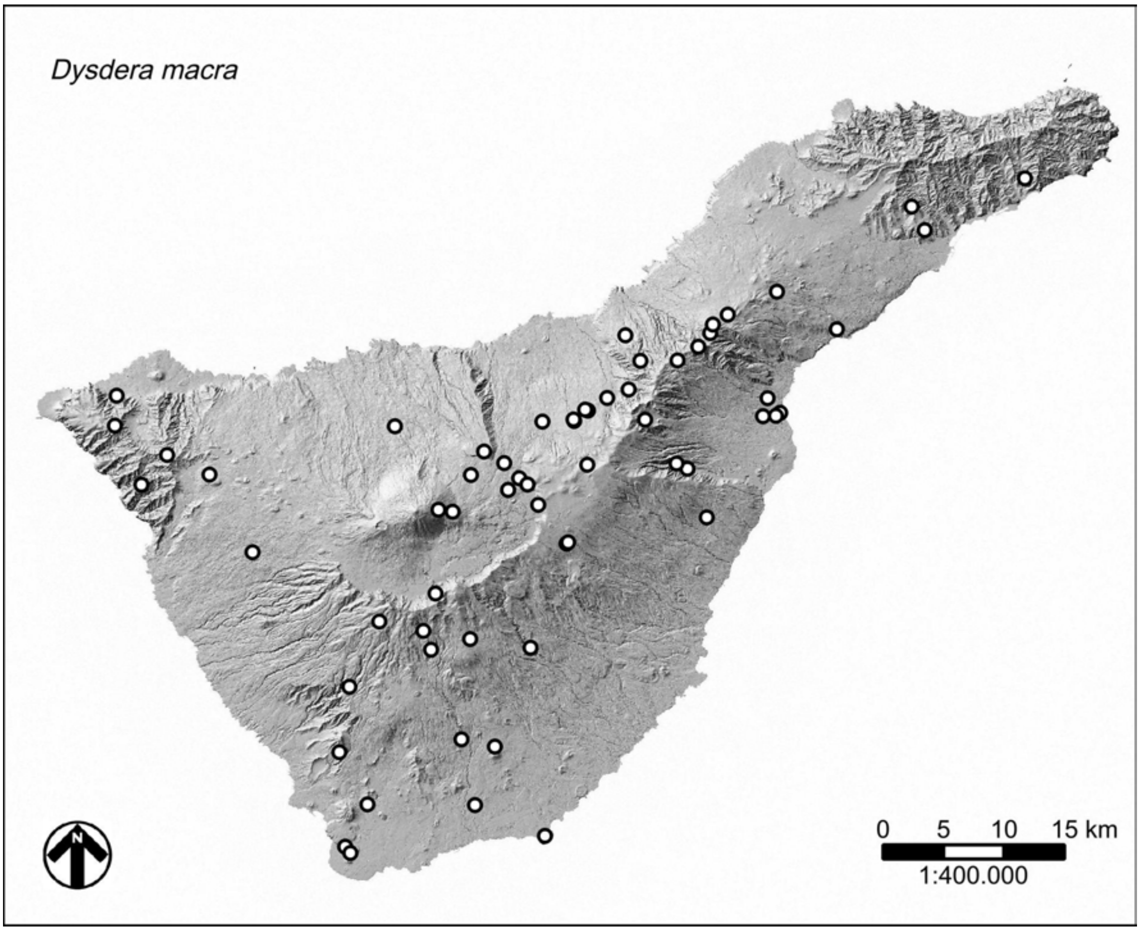
Dysdera macra
0 5 10 15 km
1:400.000
N

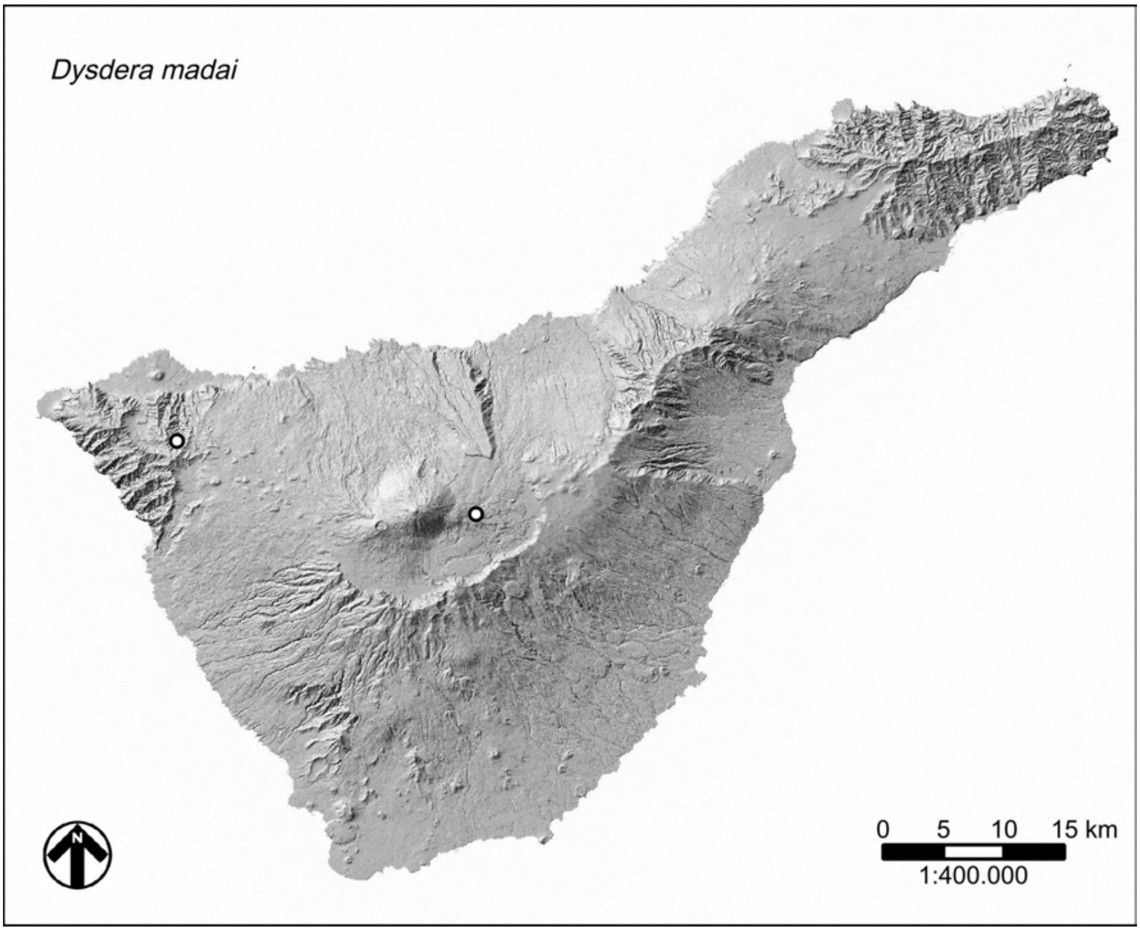
Dysdera madai
0 5 10 15 km
1:400.000
N

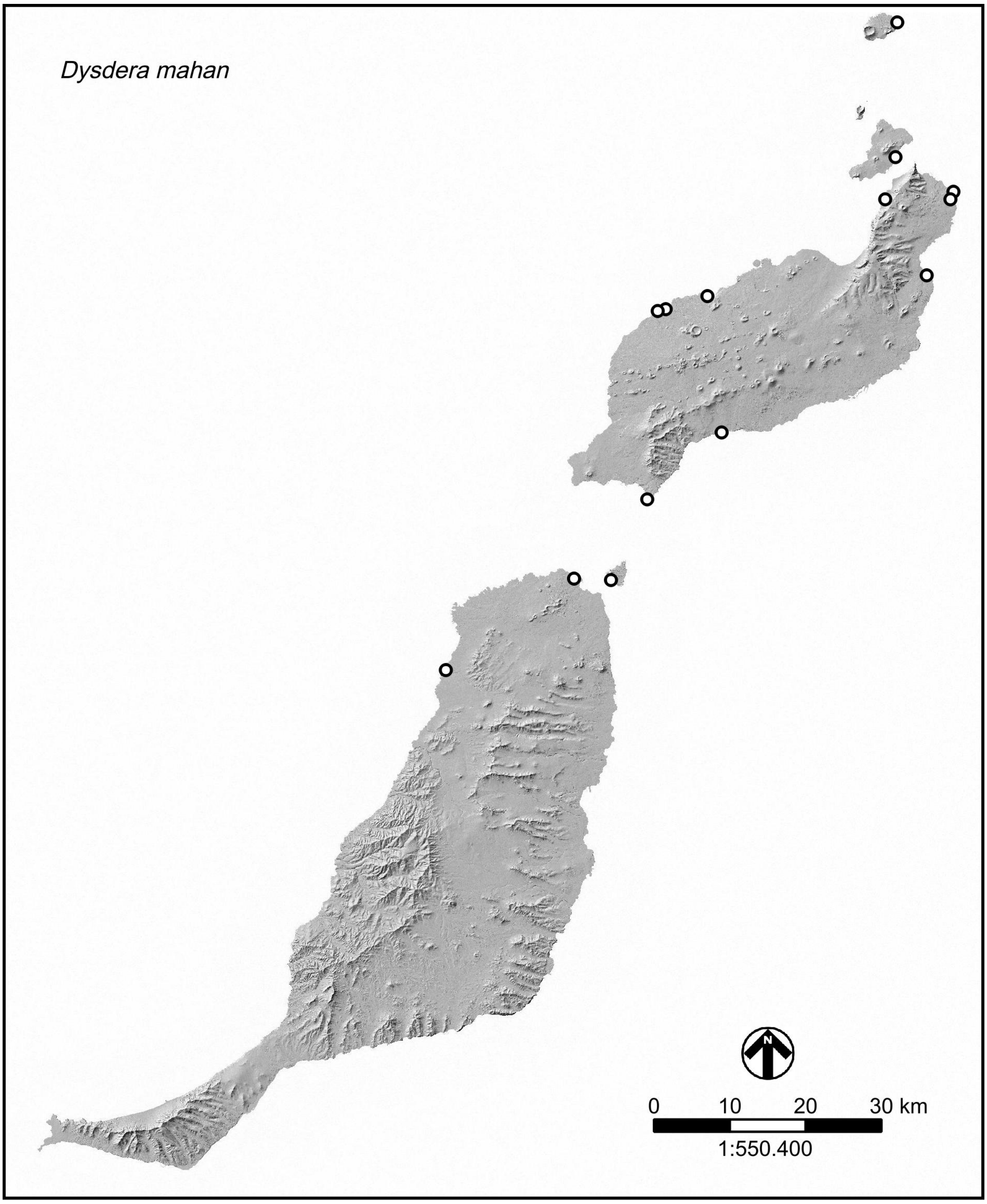
Dysdera mahan
N
0
10
20
30 km
1:550.400

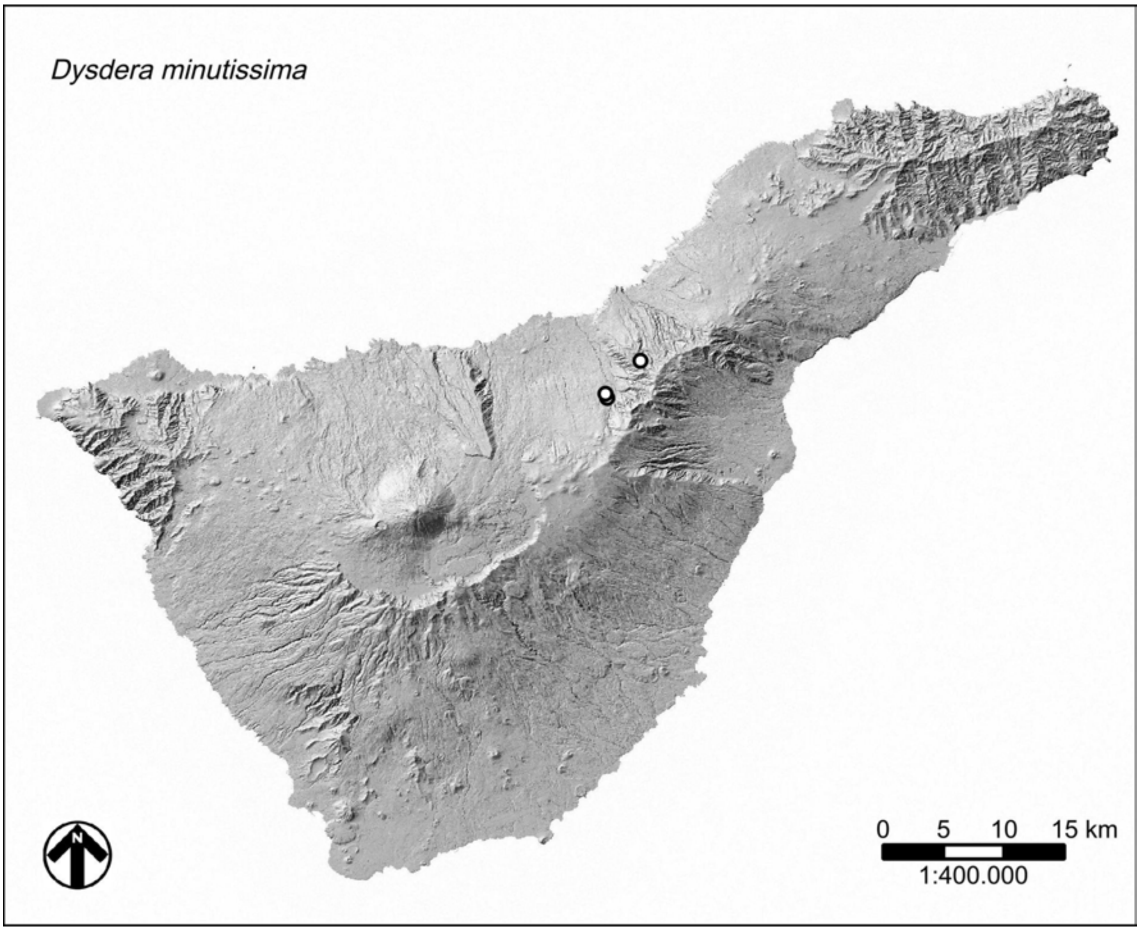
Dysdera minutissima
0 5 10 15 km
1:400.000

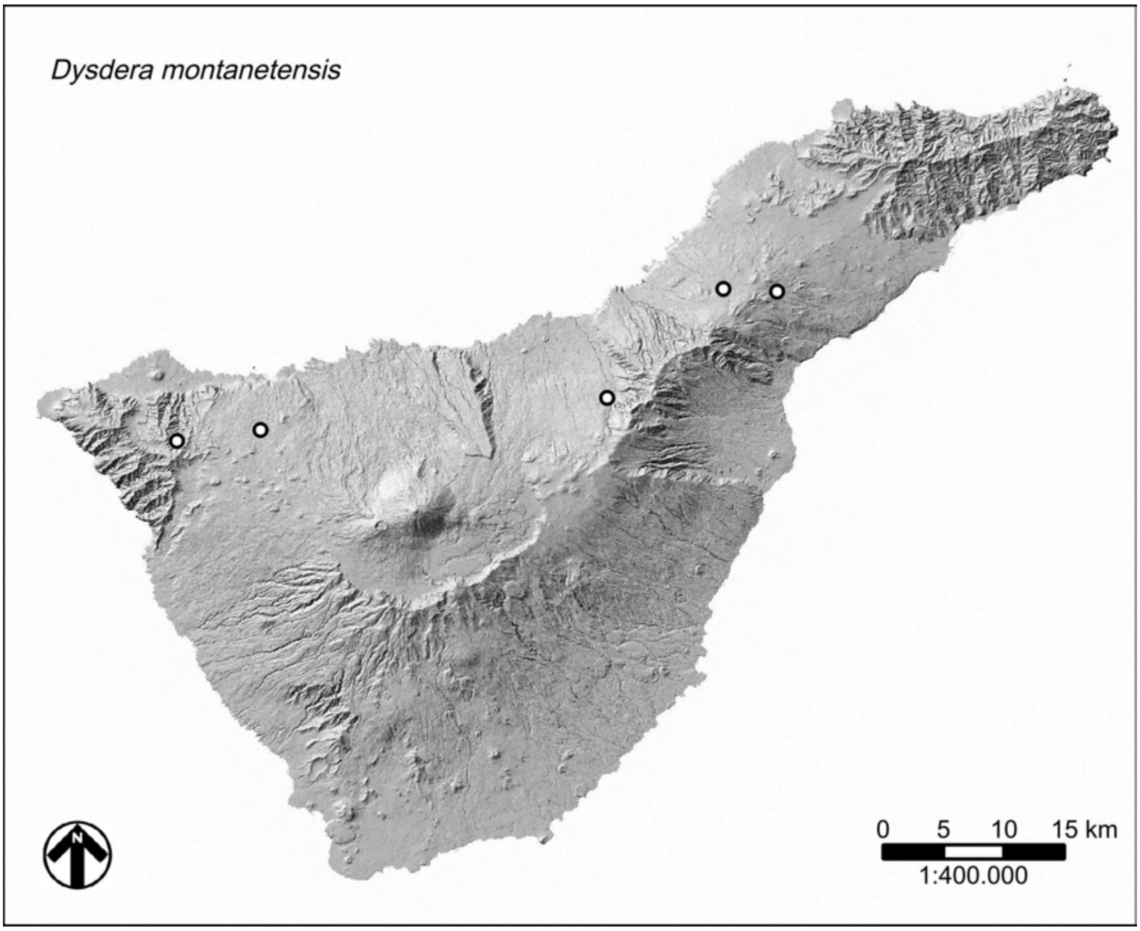
Dysdera montanetensis
0 5 10 15 km
1:400.000

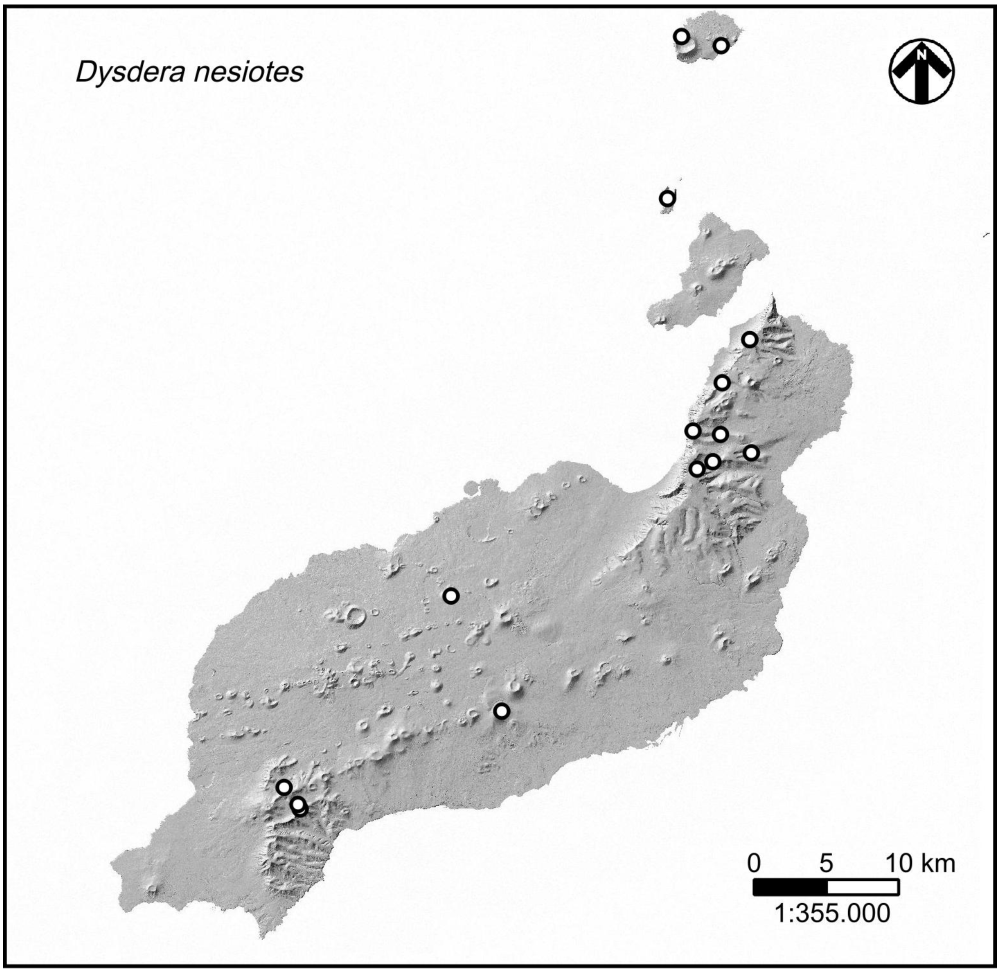

Dysdera nesiotes
N
0
5
10 km
1:355.000

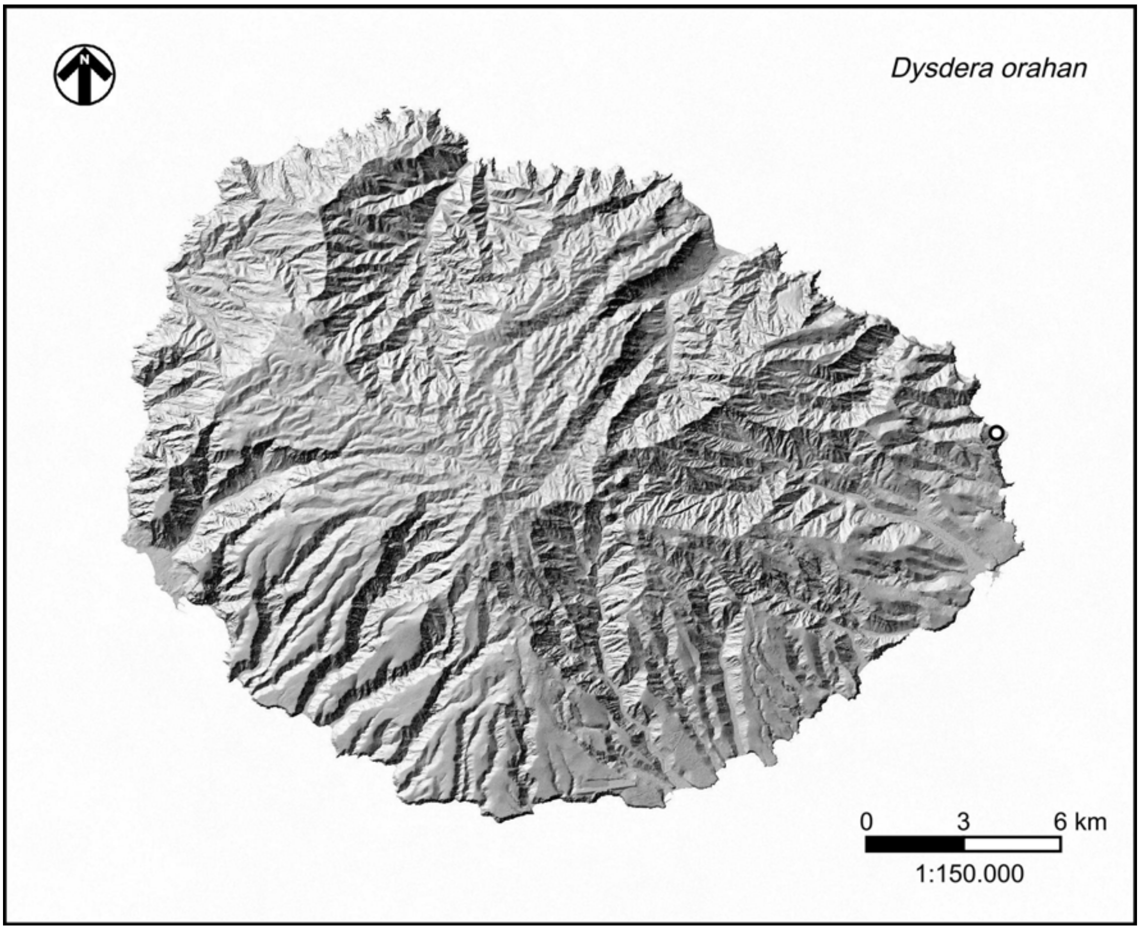
Dysdera orahan
0 3 6 km
1:150.000

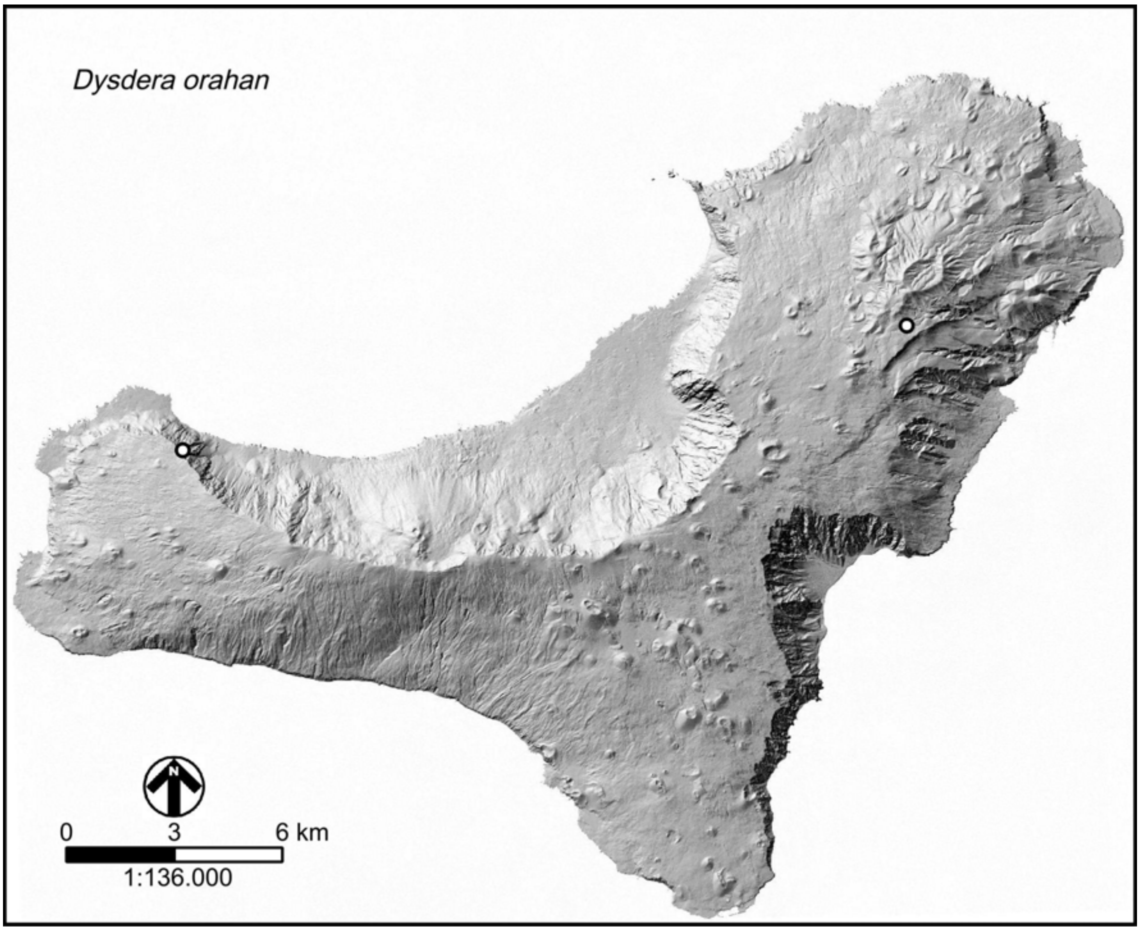
Dysdera orahan
0 3 6 km
1:136.000

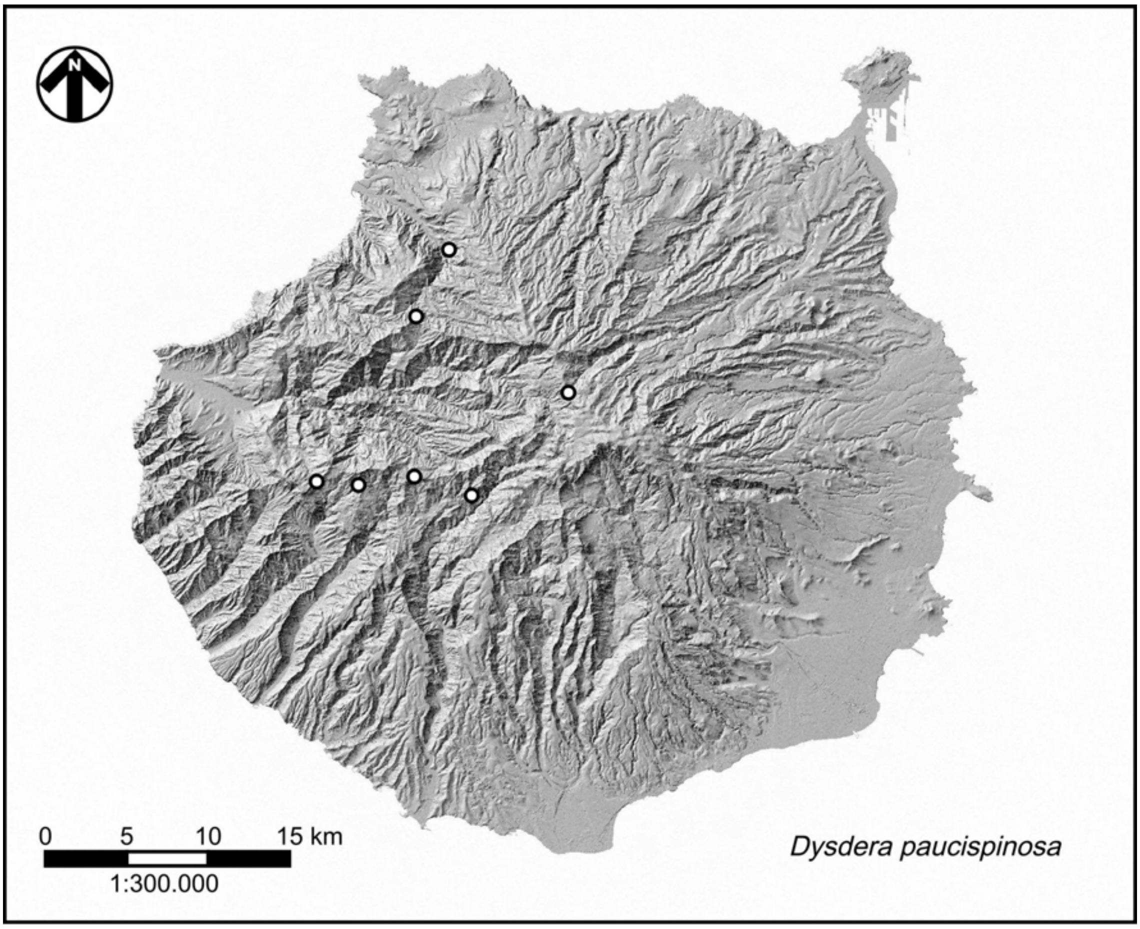
N
0
5
10
15 km
1:300.000
Dysdera paucispinosa

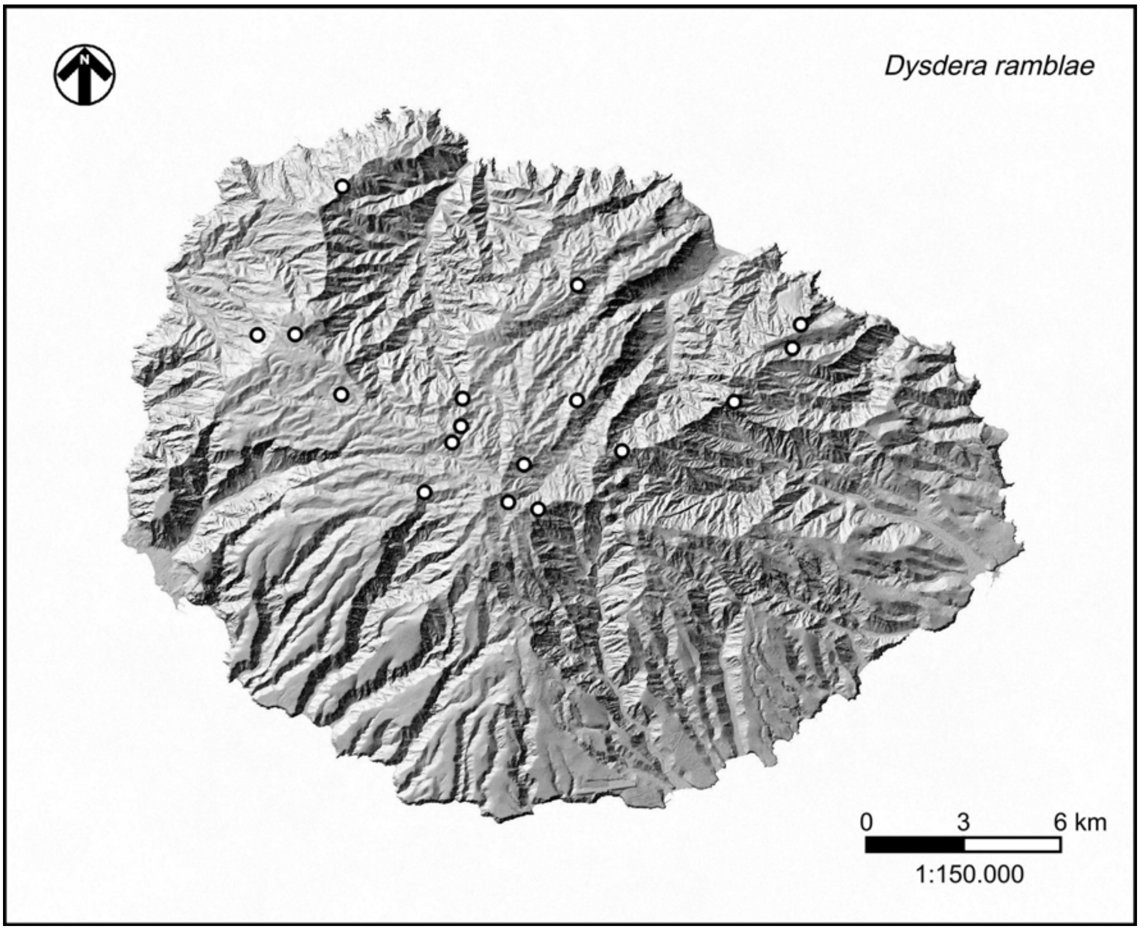
N
Dysdera ramblae
0
3
6 km
1:150.000

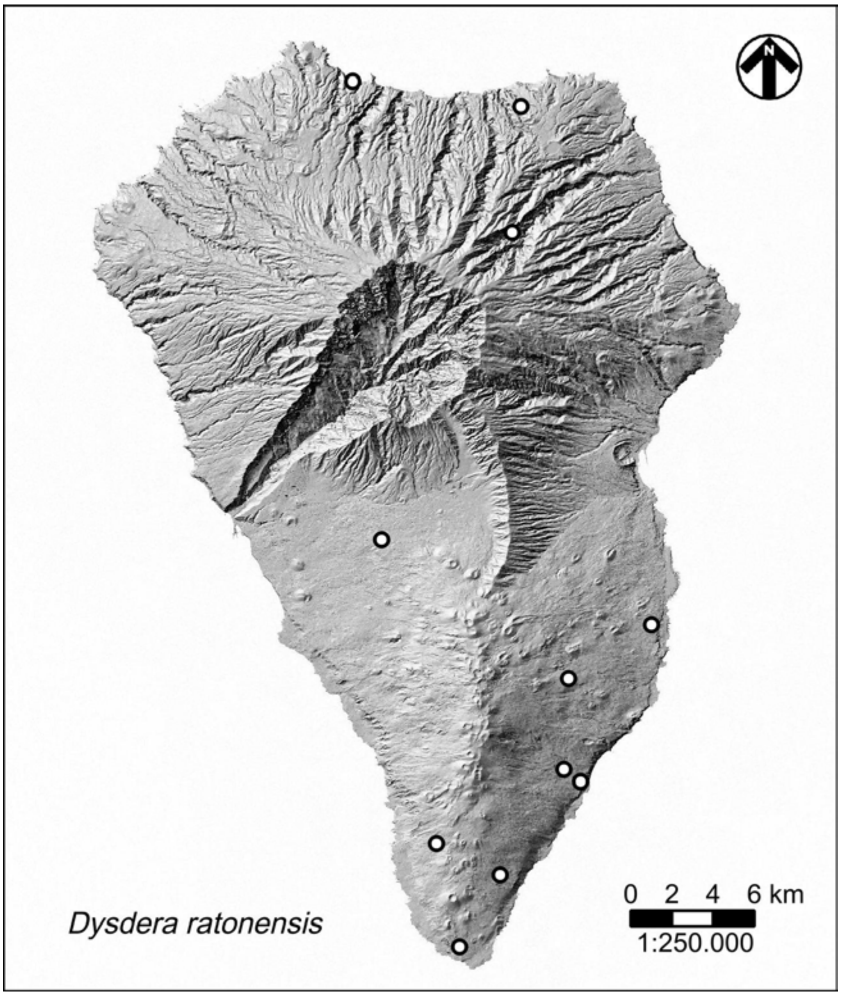
N
0 2 4 6 km
1:250.000
Dysdera ratonensis

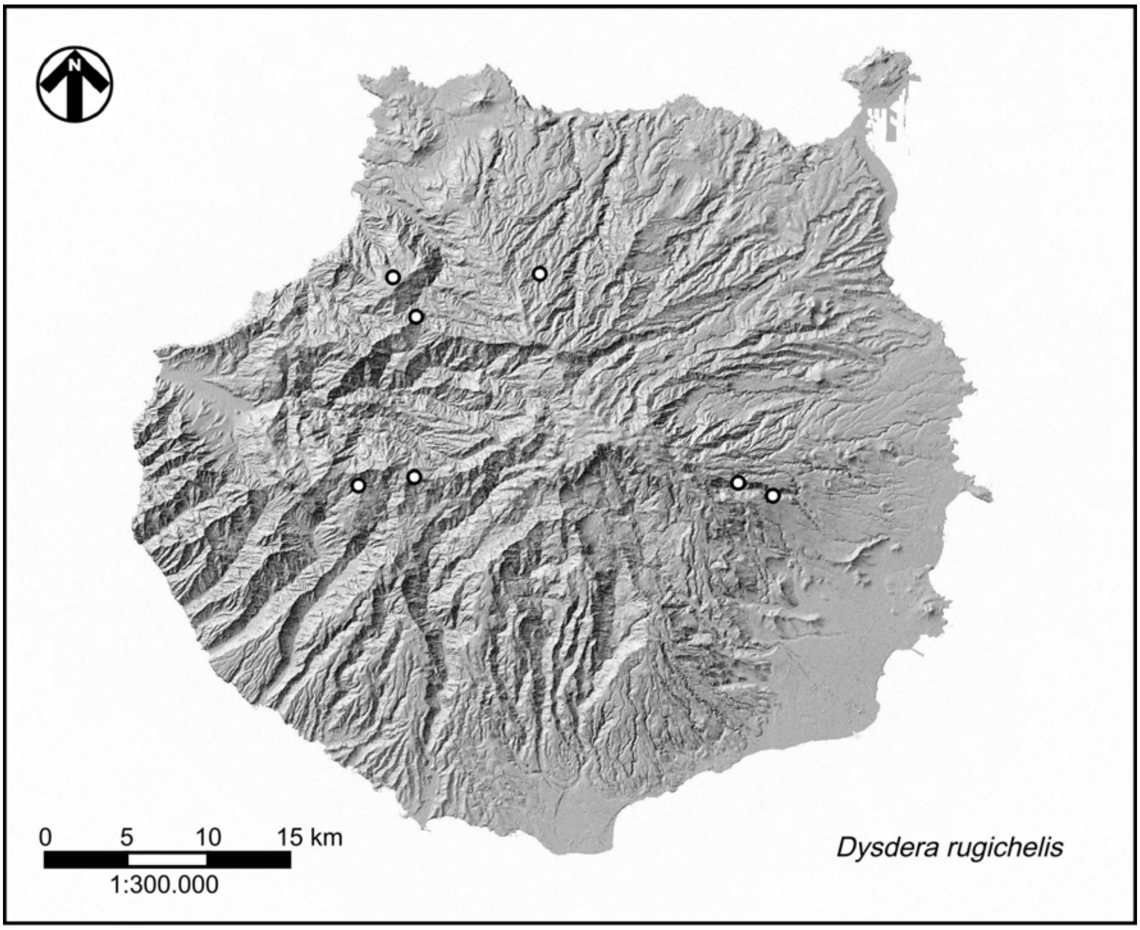
N
0 5 10 15 km
1:300.000
Dysdera rugichelis

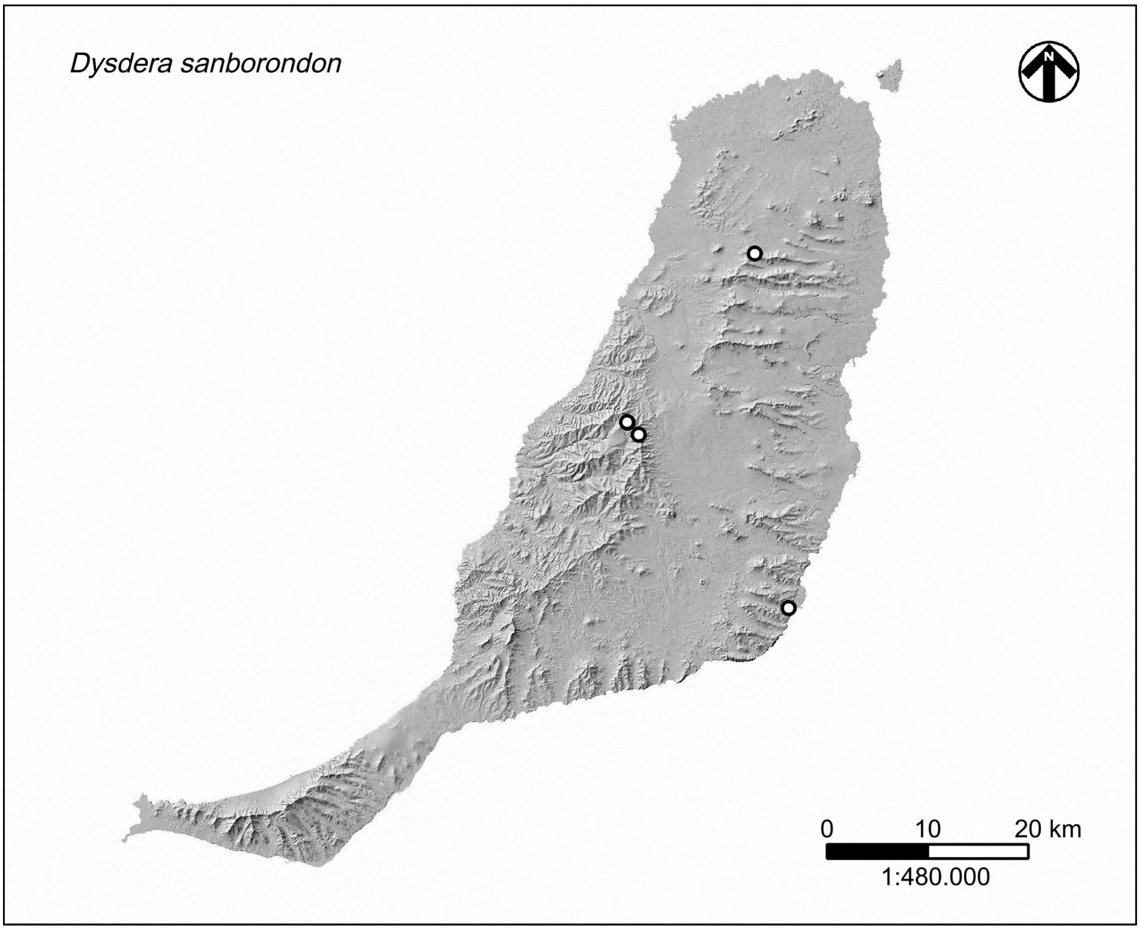
Dysdera sanborondon
N
0 10 20 km
1:480.000

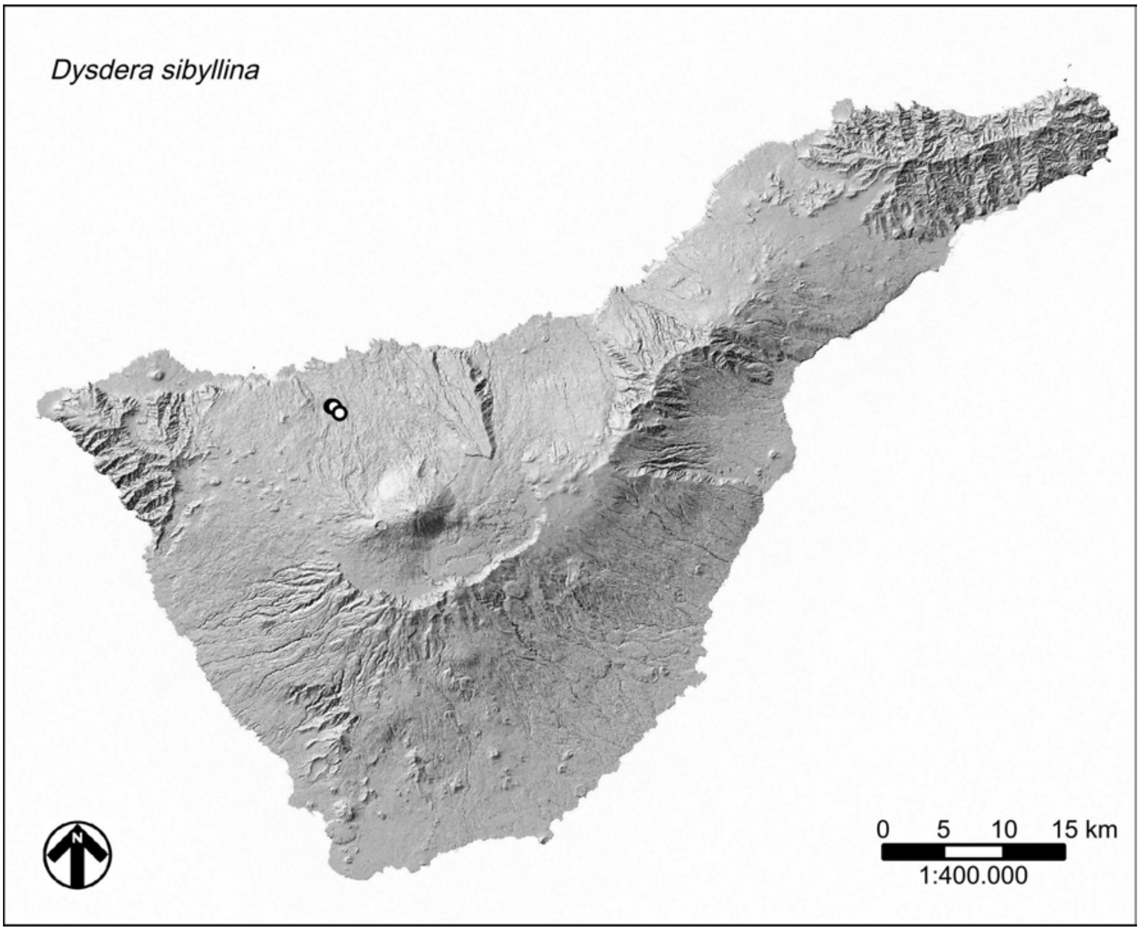
Dysdera sibyllina
N
0 5 10 15 km
1:400.000

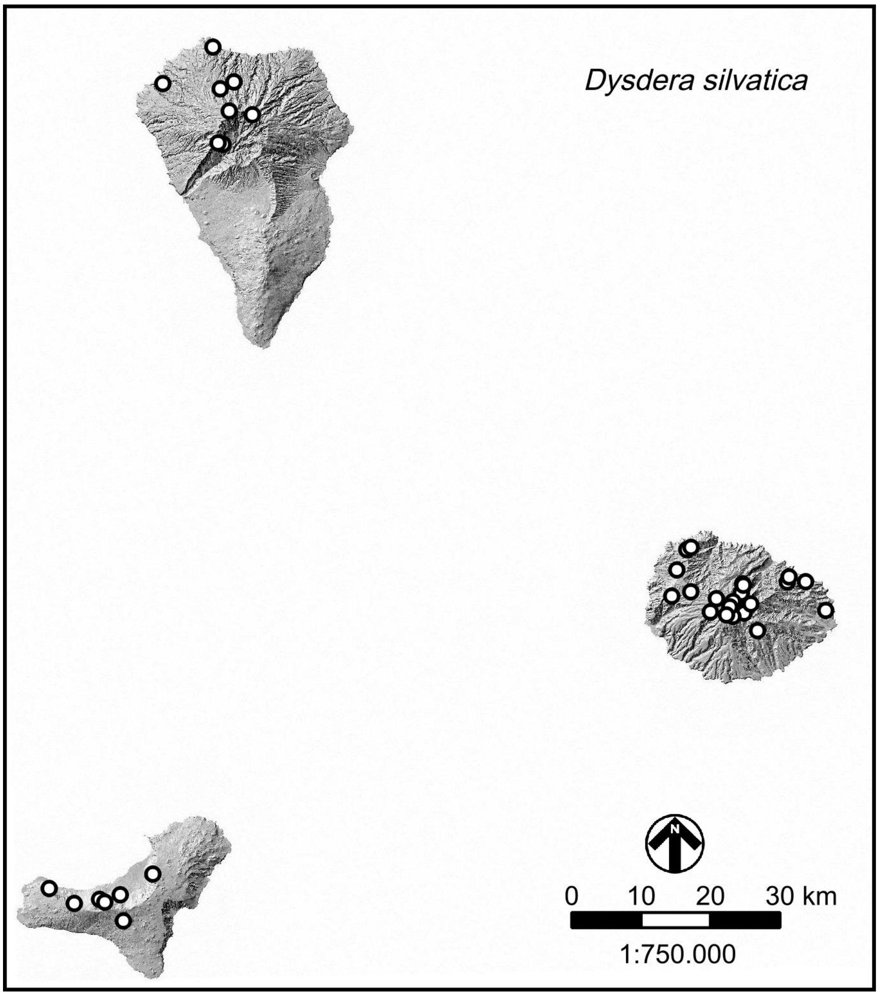
Dysdera silvatica
N
0 10 20 30 km
1:750.000

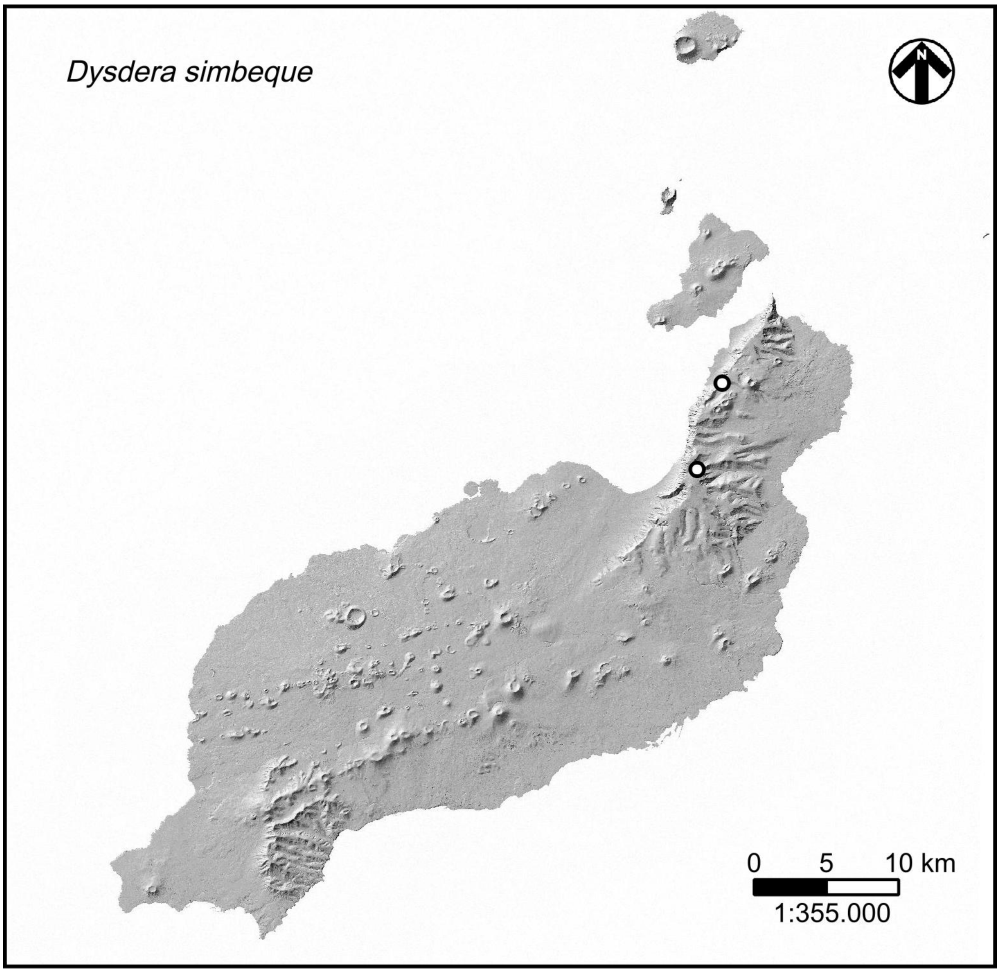
Dysdera simbeque
N
0 5 10 km
1:355.000

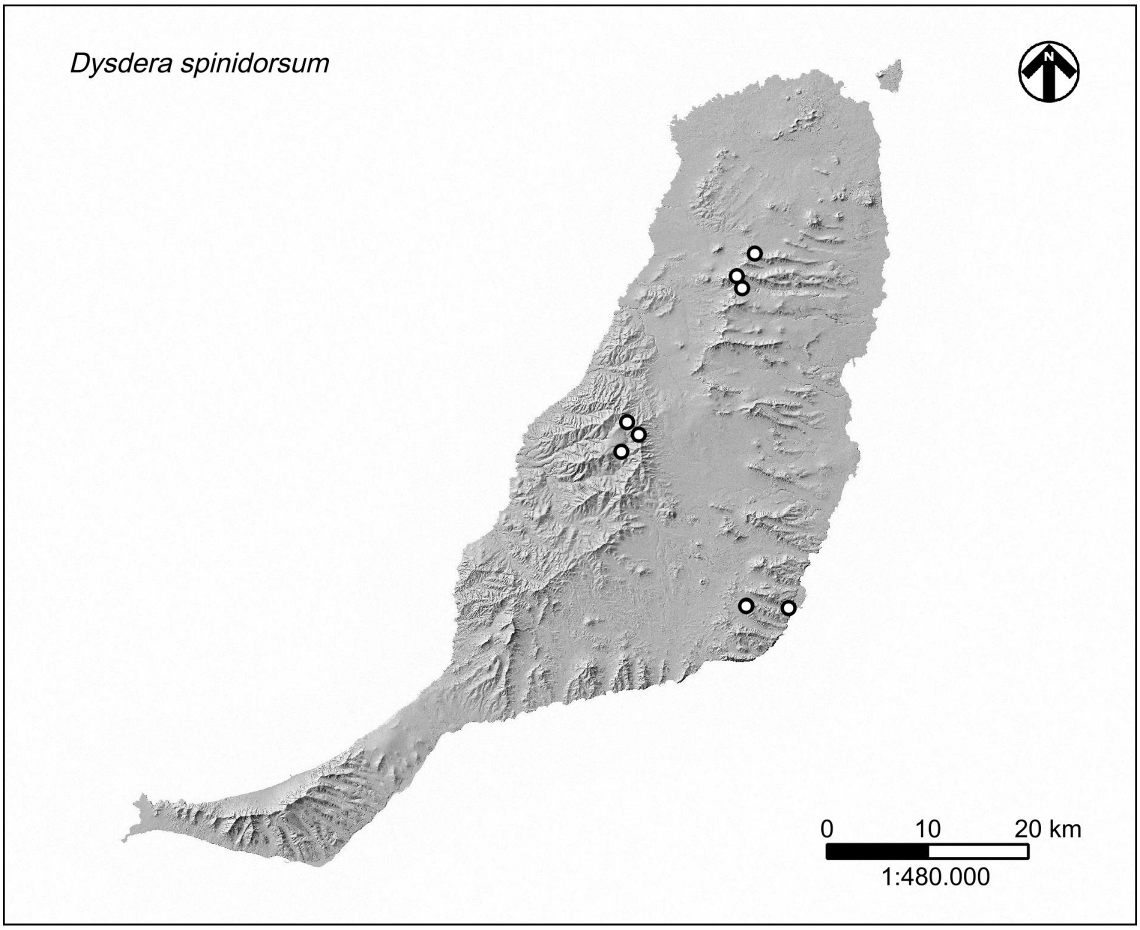
Dysdera spinidorsum
0
10
20 km
1:480.000

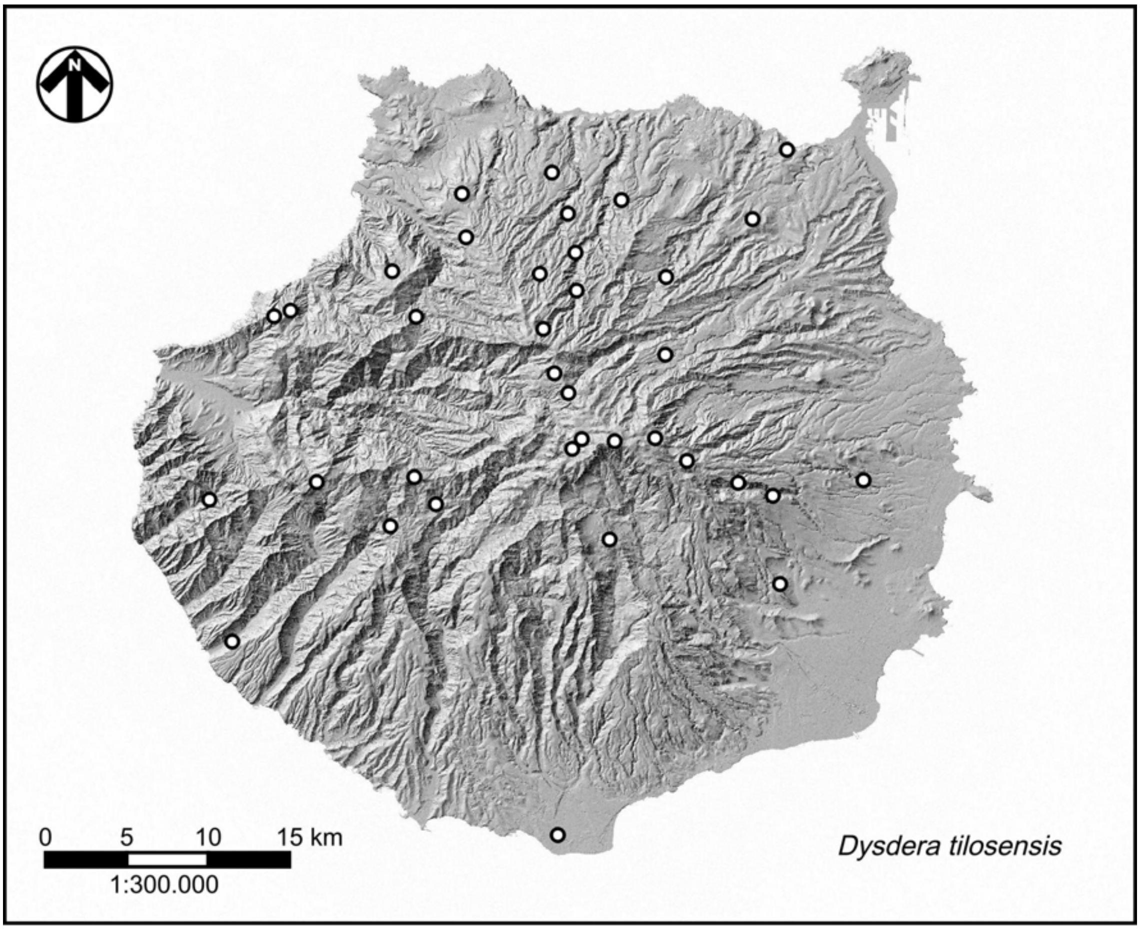
0
5
10
15 km
1:300.000
Dysdera tilosensis

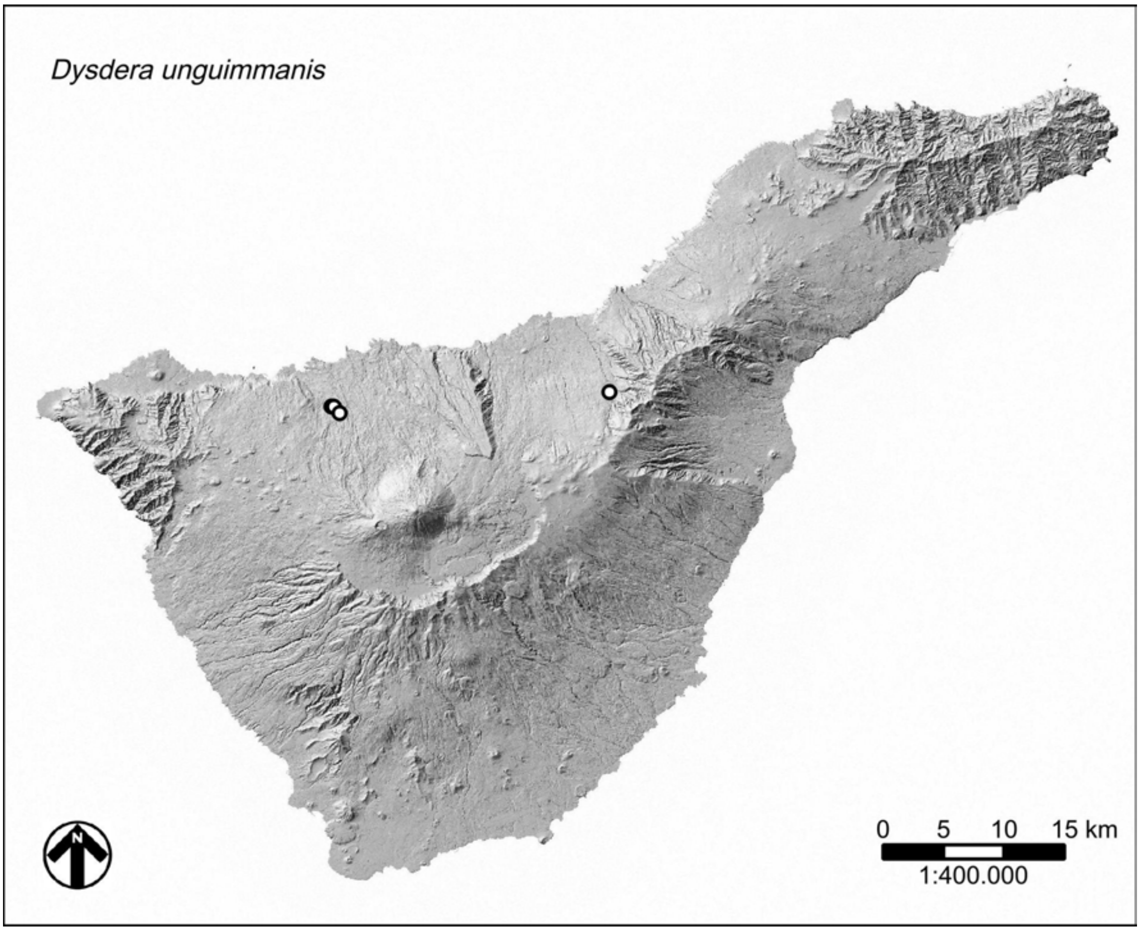

Dysdera unguimmanis
0 5 10 15 km
1:400.000

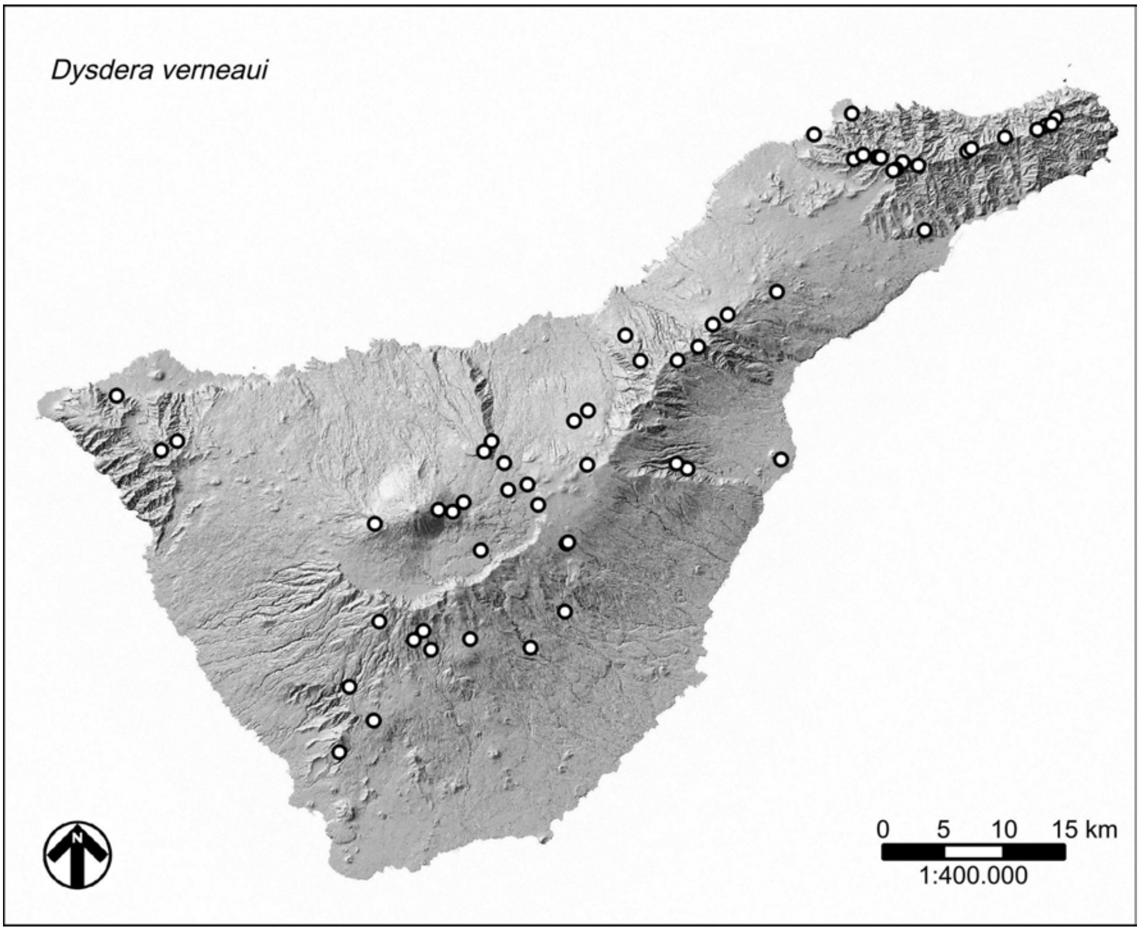

Dysdera verneaui
0 5 10 15 km
1:400.000

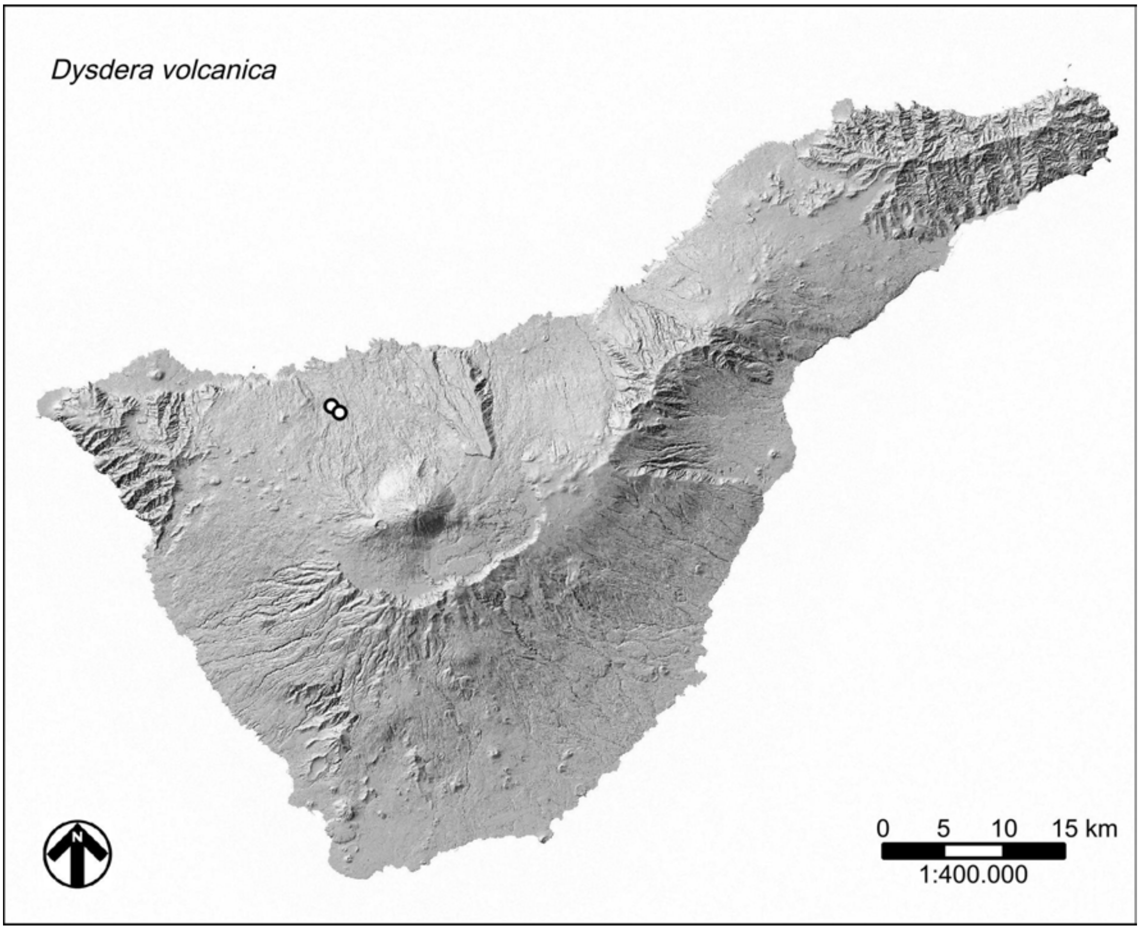
Dysdera volcanica
N
0 5 10 15 km
1:400.000

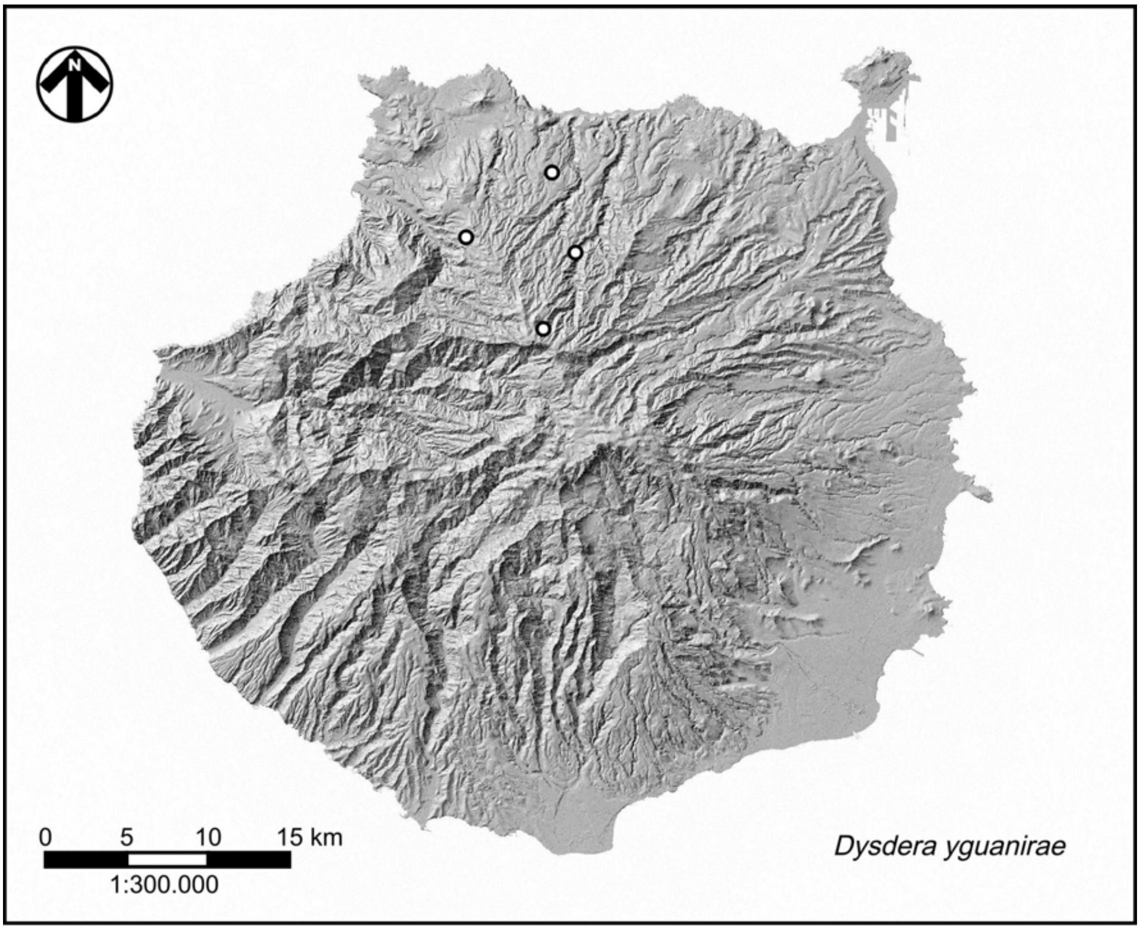
N
0 5 10 15 km
1:300.000
Dysdera yguanirae
